# Vinyl Ether/Tetrazine Pair for the Traceless Release of Alcohols in Cells

**DOI:** 10.1002/anie.201609607

**Published:** 2016-12-08

**Authors:** Ester Jiménez‐Moreno, Zijian Guo, Bruno L. Oliveira, Inês S. Albuquerque, Annabel Kitowski, Ana Guerreiro, Omar Boutureira, Tiago Rodrigues, Gonzalo Jiménez‐Osés, Gonçalo J. L. Bernardes

**Affiliations:** ^1^ Department of Chemistry University of Cambridge Lensfield Road CB2 1EW Cambridge UK; ^2^ Instituto de Medicina Molecular, Faculdade de Medicina Universidade de Lisboa Avenida Professor Egas Moniz 1649-028 Lisboa Portugal; ^3^ Departamento de Química Universidad de La Rioja, Centro de Investigación en Síntesis Química 26006 Logroño Spain; ^4^ Institute of Biocomputation and Physics of Complex Systems (BIFI) University of Zaragoza, BIFI-IQFR (CSIC) Zaragoza Spain

**Keywords:** caged compounds, ethers, heterocycles, drug delivery, fluorescent probes

## Abstract

The cleavage of a protecting group from a protein or drug under bioorthogonal conditions enables accurate spatiotemporal control over protein or drug activity. Disclosed herein is that vinyl ethers serve as protecting groups for alcohol‐containing molecules and as reagents for bioorthogonal bond‐cleavage reactions. A vinyl ether moiety was installed in a range of molecules, including amino acids, a monosaccharide, a fluorophore, and an analogue of the cytotoxic drug duocarmycin. Tetrazine‐mediated decaging proceeded under biocompatible conditions with good yields and reasonable kinetics. Importantly, the nontoxic, vinyl ether duocarmycin double prodrug was successfully decaged in live cells to reinstate cytotoxicity. This bioorthogonal reaction presents broad applicability and may be suitable for in vivo applications.

Bioorthogonal chemistry for covalently conjugating synthetic molecules at a predefined protein residue has been a major focus of research in the past two decades.^1^ Very recently, focus has been placed on reactions which can instead cleave specific bonds under bioorthogonal conditions.^2^ This strategy holds great potential for the precise spatiotemporal control of protein function in vivo.1c, 2 For example, photodeprotection of a genetically encoded caged cysteine could be used to reveal the active native protein in live cells.^3^ Similarly, palladium‐mediated depropargylation,^4^ phosphine‐mediated Staudinger reduction,^5^ and tetrazine‐triggered inverse electron‐demand Diels–Alder (IEDDA) elimination reactions^6^ were successfully employed to restore the activity of proteins bearing a caged lysine residue in the active site. Bond‐cleavage reactions are also attractive for drug‐delivery applications. Palladium‐catalyzed deprotection of a 5‐fluoroacil prodrug was shown as a method for controlled drug release in vivo.^7^ The IEDDA reaction between a tetrazine and a caged doxorubicin derivative efficiently releases the cytotoxic drug.^8^ Strategies based on IEDDA elimination reactions with tetrazines are particularly attractive for decaging relevant molecules in cells and interrogating biology, because of the favorable kinetics and the abiotic nature of tetrazines when compared to photo‐ and metal‐catalyzed reactions. One limitation, however, has been the breadth of protecting groups available for stable, yet conditionally reversible linkages. Typically, IEDDA elimination reactions have been used with strained alkene protecting groups connected through a carbamate, thus resulting in a cascade release of a primary amine (Figure 1 a).^2^, ^9^ Furthermore, the reduced metabolic stability of strained alkenes constitutes a major caveat for its utility. For instance, *cis*‐cyclooctene easily isomerizes to the non‐reactive *trans*‐cyclooctene, thus limiting the efficiency of the decaging process in cells.^10^

**Figure 1:**
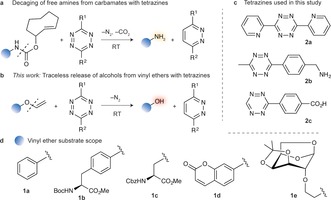
a) Tetrazine‐mediated decaging of amines from strained alkenes connected through a carbamate linker. b) Decaging of alcohols from vinyl ethers triggered by c) tetrazines. d) Vinyl‐ether‐caged alcohols studied.

Herein, we report the development of a vinyl ether/tetrazine system as IEDDA reaction partners for the traceless decaging of alcohol‐containing molecules. We demonstrate the broad applicability of this reaction on several chemotypes, including the protected amino acids serine and tyrosine, an 1,6‐anhydro sugar, a fluorophore, and a drug. Importantly, the reaction proceeds under physiological conditions (aqueous buffer pH 7.4 and 37 °C) and was applied to activate a potent toxic derivative of the drug duocarmycin in cancer cells.

To harness the current chemical biology toolbox and develop a broadly applicable technology for the controlled release of alcohol‐containing chemotypes, we set out to develop bioorthogonal bond‐cleavage reactions. In particular, we envisaged that vinyl ethers could efficiently mask both aliphatic and aromatic hydroxy groups, and be used for traceless release of alcohols through a tetrazine IEDDA bond‐cleavage reaction. In fact, the reactivity of the vinyl group with tetrazines has been detailed in organic synthesis^11^ and such a reactive pair was very recently employed to visualize and detect RNA under bioorthogonal conditions.^12^ We first used commercial phenyl vinyl ether **1 a** as a model compound, and tetrazine **2 a** to challenge our decaging hypothesis (Figure 1 b–d). After reaction with **2 a**, phenol (**3 a**) and 3,6‐di(pyridin‐2‐yl)pyridazine (**4 a**) were obtained in 49 % and 61 % yield, respectively (Table 1). The reaction was performed in dichloromethane to ensure that all the reagents were soluble and at room temperature with only two molar equivalents of tetrazine. Increasing the amount of tetrazine showed no significant improvement in reaction yield. To assess the scope of the reaction we then synthetized vinyl ether derivatives **1 b**–**e**.^13^ Tyrosine and serine residues play a paramount role in the building of binding pocket architecture and controlling catalytic cycles, for example, as in tyrosine kinases and serine proteases. Indeed, bioorthogonal decaging of catalytically crucial residues is emerging as a disruptive technology in chemical biology.^2^ Furthermore chromone‐based fluorophores and sugars can efficiently be used to interrogate biological systems.^14^ Remarkably, decaging vinyl ether derivatives of such molecules **1 b**–**e** with **2 a** gave the corresponding free hydroxy derivatives in good yields (50–68 % yields after purification by column chromatography; Table 1). Of note, the vinyl ether reagents were generally stable under biocompatible conditions (PBS pH 7.4 at 37 °C) over 8 hours, as assessed by HPLC/UV (Table 1; see the Supporting Information). While some degree of instability was observed for **1 c**, for instance, the free hydroxy‐containing molecule was not detected.

**Table 1:** Stability of vinyl ethers **1 a**‐**e** and their decaging with tetrazine **2 a**.

Vinyl	Yield^[c]^		Conv.	Stability^[d]^
ethers^[a]^	Alcohol	Pyridazine	[%]	[%]
**1 a** ^[b]^	61	49	100	100^[e]^
**1 b**	68	65	73	100
**1 c**	57	72	100	77
**1 d**	50	47	56	100
**1 e**	65	50	100	n.d.^[f]^

[a] The reactions were performed in dichloromethane at room temperature with 1 equiv of vinyl ether (100 mm) **1 a**–**e** and 2 equiv of tetrazine **2 a** (200 mm) for 72 hours. [b] The reaction was complete after 40 hours. [c] Yield of isolated product. [d] The stability (as % of remaining starting compound) was assessed by HPLC in PBS pH 7.4 at 37 °C with a concentration of vinyl ether of 200 μm using acetophenone as an internal standard. [e] 10 % H_2_O in DMF. [f] **1 e** does not absorb in the UV. n.d.=not determined.

After confirming the stability of the vinyl ether molecules in pH 7.4 buffer and their successful tetrazine‐mediated decaging, we proceeded to study the reaction mechanism in detail through quantum mechanics at the M06‐2X/6‐31+G(d,p) level of theory (Figure 2 a; see the Supporting Information). Unlike tetrazine reactions with highly reactive strained alkenes,^8^ our data indicate that the first step, that is, the IEDDA cycloaddition, is the rate‐limiting step of the reaction (**TS1**, Δ*G* ^≠^≈25 kcal mol^−1^; reaction time ca. 3 days) followed by very fast retro‐Diels Alder (**TS2** *anti*, Δ*G* ^≠^≈7 kcal mol^−1^) and phenoxy group cleavage (**TS3**, Δ*G* ^≠^≈11 kcal mol^−1^). Once the 4‐phenoxy‐4,5‐dihydropyridazine (**int2**) is obtained, it readily tautomerizes into 4‐phenoxy‐1,4‐dihydropyridazine (**int4**), which can swiftly decage through an elimination reaction. Of note, other dihydropyridazine tautomers previously reported to undergo decaging (**int3**),^6^, ^8^ are unreactive in this reaction. To support the theoretical data, we mixed **1 a** and **2 a** in 10 % D_2_O in CD_3_CN, and recorded the ^1^H NMR spectra at selected times to gain insight into the reaction mechanism (Figure 2 b). Peaks in the aromatic region assigned to the final products could be identified after 24 hours. Conversely, no peaks in the *δ*=2.5–6 ppm region could be attributed to **int2**, and the intermediate species **int3** and **int4** were observed over the course of the experiment. Given that no intermediate species were identified through ^1^H NMR analysis, unlike amines decaging from carbamates,^8^ our data clearly supports that the Diels–Alder cycloaddition is rate‐limiting in this case. A fast and irreversible decaging step after IEDDA cycloaddition is thus responsible for the experimental observations, and fully in line with the predicted reaction coordinate diagram (see Figure S4 in the Supporting Information). Furthermore, our spectral data also confirm that no reaction intermediates are trapped, and that all of them evolve to a common tautomer before the decaging step (Figure 2 b).

**Figure 2:**
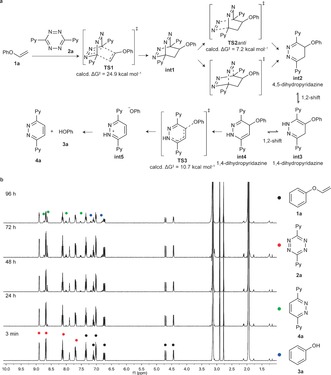
a) Proposed mechanism based on quantum mechanics for the IEDDA cycloaddition of **1 a** and **2 a**, followed by in situ alcohol release. Only the relevant activation free energies (Δ*G* ^≠^) are shown. The initial cycloaddition is the rate‐limiting step. After very fast nitrogen cleavage, different dihidropyridazine tautomers **int2**–**int4** equilibrate before irreversibly decaging to the experimentally obtained products (**3 a** and **4 a**). See Figure S4 in the Supporting Information for the whole calculated minimum energy pathway. b) ^1^H NMR release studies of **1 a** upon reaction with **2 a**. The reaction was performed at 3 mm of **1 a** and **2 a** in 10 % D_2_O/CD_3_CN. The reaction was monitored for 96 h. While the reaction was not always complete at 96 h, the results obtained were consistent with the mechanism supported by the theoretical calculations. Ph=phenyl, Py=pyridine.

With a detailed assessment of the reaction mechanism, we then performed kinetic studies by following the decrease of the tetrazine absorbance, that is, the rate‐limiting step, in the visible region. We used **1 a** and screened tetrazines **2 a**–**c**, which included different substituents (Figure 1 and Table 2). Stability studies of **1 a** in the system solvent used (10 % H_2_O in DMF) showed no significant degradation after 8 hours. As expected, tetrazines bearing electron‐withdrawing groups (**2 a**) led to faster reactions compared to the one bearing electron‐donating ones (**2 b**). Finally, **2 c** proved to have the fastest kinetics, probably because of the reduced steric hindrance brought about by hydrogen‐substituted tetrazines.^15^ We also compared the kinetics of the vinyl ether/tetrazine decaging with a very reactive, strained alkene as reference, 5‐norbornen‐2‐ol. The second‐order rate constant determined (*k*_2_=0.189 m ^−1^ s^−1^) in 10 % H_2_O in DMF, although lower, compares well with literature values for the same reaction (*k*_2_=1.3 m ^−1^ s^−1^ in H_2_O at 20 °C).^16^ The differences observed are attributed to the faster IEDDA reactions in polar protic solvents.

**Table 2:** Kinetics of the reaction of **1 a** with the tetrazines **2 a**–**c**.

**2** ^[a]^	Dienophile	*k*_2_×10^−4^ m ^−1^ s^−1^
**2 a**	**1 a**	3.92±0.11
**2 b**	**1 a**	0.063±0.013
**2 c**	**1 a**	5.37±0.13
**2 a**	5‐norbornen‐2‐ol	1890±40

[a] The reactions were performed in 10 % H_2_O in DMF and were followed by UV through the decay of UV absorption of the tetrazines. An excess of 150–350 fold of **1 a** was used. In the case of 5‐norbornen‐2‐ol the kinetic rate was determined using the same solvent system with **2 a** with a 20‐ to 100‐fold excess of 5‐norbornen‐2‐ol.

To show the potential bioorthogonality of this bond‐cleavage reaction, we next studied its use for the decaging of a vinyl ether fluorogenic probe in live cells (Figure 3 a). To this end, we used a vinyl ether nonfluorescent coumarin derivative (**1 d**) and **2 c**, as both were shown to be nontoxic to HepG2 cells at the concentrations used (Figure 3 b; see the Supporting Information for A549 cells). In short, after incubation with 25 μm of **1 d** for 5 hours, **2 c** (10 μm) was added to the cells for 4 hours. At this time, cells were imaged using confocal microscopy and the turn‐on fluorescence of the released coumarin was recorded as a result of the successful tetrazine decaging of the vinyl ether protecting group installed in **1 d** (Figure 3 c). Importantly, this study highlights the biocompatibility of this approach for turn‐on live cell imaging applications.

**Figure 3:**
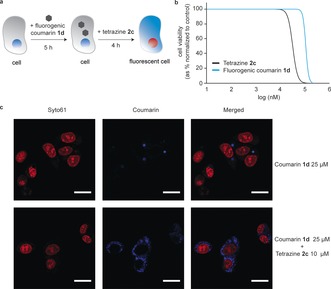
a) General protocol for **2 c**‐mediated intracellular decaging of fluorogenic coumarin **1 d**. b) Cytotoxicity dose‐response curves of **2 c** and **1 d** in HepG2 cells, obtained after 48 hours of exposure. c) Detection of fluorescent coumarin (blue) upon tetrazine decaging inside HepG2 cells by confocal microscopy. Cells were incubated for 5 hours with 25 μm **1 d** and then for 4 hours with 10 μm of **2 c** (bottom panel) or equivalent vehicle control (top panel). Before image acquisition, nuclei were stained with Syto61 (red). Scale bar represents 20 μm.

The targeted delivery of drugs to diseased tissues remains a topic of intensive study, and an unsolved issue in modern drug discovery. Currently, alcohol‐containing drugs account for approximately 50 % of all small FDA‐approved chemical entities (cf. DrugBank v5.0; see the Supporting Information), thus providing ample opportunity for the design of innovative drug delivery constructs. As a proof‐of‐concept for our technology we assessed the spatiotemporal delivery of a duocarmycin‐like natural product (**5**; Figure 4 a). Duorcamycins are isolated from *Streptomyces* spp. bacteria^17^ and have attracted considerable attention as payloads in antibody–drug conjugates, given their potent cytotoxic activity.^18^ Interestingly, halogen‐bearing duorcamycin cytotoxics undergo a Winstein spirocyclization reaction to afford the bioactive cyclopropanyl, a DNA‐alkylating species. This feature has been explored in antibody‐directed enzyme prodrug therapy, where a nontoxic glycosydic derivative of duocarmycin is activated by a conjugate of an enzyme and a tumor‐specific antibody.^19^ To demonstrate that our tetrazine‐mediated IEDDA cleavage of vinyl ethers could be applied for the traceless release of an alcohol‐containing drug, we synthetized the *N*‐Ac‐double prodrug **5** in three steps from the *N*‐Boc‐protected 1,2,9,9a‐tetrahydrocyclopropa[1,2‐*c*]benz[1,2‐*e*]‐indol‐4‐one (CBI) starting material (see the Supporting Information for details).^20^ We chose this simple duocarmycin analogue featuring only an acetyl group attached to the DNA‐alkylating CBI core because it has been shown to be very toxic to rapidly replicating cells.^21^ We envisioned that upon tetrazine IEDDA deprotection of the vinyl ether, the halogen prodrug **6** would be readily formed and undergo a rapid Winstein spirocyclization reaction to afford active drug **7**. This tetrazine‐triggered cascade formation of an active drug through an intermediate prodrug is known as the double prodrug concept.^22^

**Figure 4:**
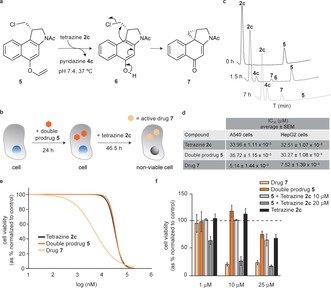
a) The N‐Ac CBI double prodrug **5** reacting with **2 c** leading to the formation of the intermediate **6** which undergoes a Winstein spirocyclization to afford the bioactive cyclopropanyl **7**. b) General protocol for **2 c**‐mediated intracellular decaging of **5**. c) HPLC time‐course of the reaction between **5** and **2 c**. d) Half maximal inhibitory concentration (IC_50_) of **2 c**, **5**, and **7** in A549 and HepG2 cells. e) Cytotoxicity fitted dose‐response curves of **2 c**, **5**, and **7** in A549 cells, obtained after 46 hours 30 min of exposure. f) Cytotoxic effects of intracellular activation of the prodrug **5** by **2 c** inside A549 cells. For data on HepG2 cells, see the Supporting Information.

Next we studied the stability of the double prodrug **5** in PBS pH 7.4 at 37 °C using HPLC. While species **6** and active drug **7** were not formed, we detected some degree of degradation of **5** over time (of note, formation of neither **6** or **7** was observed). Importantly, **5** was found to be less toxic when compared with the active drug **7** in both HepG2 and A549 cells (Figure 4 d,e; see the Supporting Information). Having a suitable masked vinyl ether double prodrug in hand, we performed a decaging reaction under physiological conditions (PBS pH 7.4 at 37 °C) with **2 c**. Remarkably, close to complete formation of **7** was achieved after 7 hours at 37 °C in PBS pH 7.4, with the short‐lived species **6** as an intermediate (Figure 4 c). After successful demonstration of decaging of **5** under physiologically relevant conditions, we next proceeded to evaluate the feasibility of this approach for the tetrazine‐mediated drug‐delivery. A549 cells were first incubated with **5** for 24 hours, after which time **2 c** was added for an additional 46.5 hour period (twice the doubling time of these cells and comparable to our cytotoxicity studies; Figure 4 b,e). Satisfyingly, we observed that at 10 μm, the product formed upon tetrazine‐decaging of **5** is as toxic as **7** alone (Figure 4 f; see the Supporting Information for identical study on HepG2 cells), thus suggesting complete drug activation in cells. Hence, this data advocates that tetrazine‐mediated bond cleavage of vinyl ethers may be used for the traceless release of alcohol‐containing drugs.

In summary, we described a vinyl ether/tetrazine pair as IEDDA reaction partners for the efficient traceless decaging of alcohol‐containing molecules in live cells. Considering the wealth of hydroxy groups in chemical probes and drugs, coupled to the need of circumventing adverse drug reactions, the spatiotemporal delivery method disclosed herein may find broad applicability in chemical biology and molecular medicine by unraveling new biology and leveraging the controlled modulation of (patho)physiological events. Additionally, and in combination with strategies for the genetic encoding of vinyl‐ether‐protected tyrosine and serine derivatives, this tetrazine IEDDA decaging reaction is likely to find use for precise control of protein function in vivo.

## Experimental Section

Decaging of vinyl ether duocarmycin prodrug in vitro: The *N*‐Ac CBI prodrug **5** was diluted in PBS pH 7.4 to a final concentration of 100 μm from a 10 mm stock in acetonitrile. Then the benzoic acid tetrazine **2 c** was added to a final concentration of 500 μm from a 50 mm stock in DMSO. The reaction was performed at 37 °C and was monitored by HPLC/UV at different times until completion.

Decaging of vinyl ether duocarmycin double prodrug **5** in cells: Cells were incubated with increasing concentrations of **5** or equivalent vehicle controls for 24 h. The culture medium was then exchanged to complete medium supplemented with increasing concentrations of tetrazine **2 c**, drug or equivalent vehicle controls. Cells were incubated for another 46.5 h until proceeding with the CellTiter‐Blue Cell Viability Assay (Promega). Relative fluorescence units (R.L.U.) were normalized to the values obtained for the appropriate vehicle controls. Bars represent the average of 3 independent experiments and error bars represent standard error of the mean (SEM).

## Conflict of interest

The authors declare no conflict of interest.

## Supporting information

As a service to our authors and readers, this journal provides supporting information supplied by the authors. Such materials are peer reviewed and may be re‐organized for online delivery, but are not copy‐edited or typeset. Technical support issues arising from supporting information (other than missing files) should be addressed to the authors.

Supplementary
