# Supplementary material for: Vinyl Ether/Tetrazine Pair for the Traceless Release of Alcohols in Cells
Source: Angew Chem Int Ed Engl. 2016 Dec 8;56(1):243–7. doi: 10.1002/anie.201609607 (PMC6485351; doi:10.1002/anie.201609607)
Supplement: Supplementary file 1 — Supplementary [file ANIE-56-243-s001.pdf]

Angewandte Chemie
Eine Zeitschrift der Gesellschaft Deutscher Chemiker

Supporting Information

Vinyl Ether/Tetrazine Pair for the Traceless Release of Alcohols in Cells

*Ester Jiménez-Moreno, Zijian Guo, Bruno L. Oliveira, Inês S. Albuquerque, Annabel Kitowski, Ana Guerreiro, Omar Boutureira, Tiago Rodrigues, Gonzalo Jiménez-Osés, and Gonçalo J. L. Bernardes**

anie_201609607_sm_miscellaneous_information.pdf

TABLE OF CONTENTS

1. ORGANIC SYNTHESIS 2

General information 2

General procedures 2

Synthesis of vinyl ethers 3

Decaging reactions 6

Synthesis of tetrazine 2c 9

2. DETERMINATION OF KINETIC RATE CONSTANTS: 10

3. STABILITY IN PBS 11

4. RELEASE STUDIES BY ^{1}H-NMR 14

5. RELEASE STUDIES OF COMPOUND 5 BY HPLC: 14

6. TABLE S1: Examples of FDA approved alcohol-containing drugs (cf DrugBank v5.0). 20

7. IN VIVO DECAGING METHODS 21

8. COMPUTATIONAL DETAILS. 24

9. NMR SPECTRA 51

10. REFERENCES 62

1. ORGANIC SYNTHESIS

General information

Chemicals were purchased and used without further purification. All solvents were purified by distillation over drying agents. Unless stated otherwise, reactions were carried out under a dry argon atmosphere in vacuum-flame dried glassware. Detection was by examination under UV light (254 nm) and by charring with potassium permanganate. Flash column chromatography was performed using silica gel [Merck, 230−400 mesh (40−63 μm)]. ^{1}H NMR and ^{13}C NMR spectra were measured in the solvent stated at 500 or 400 and 126 or 101 MHz respectively. Chemical shifts are quoted in parts per million from residual solvent peak ($CDCl_3$: ^{1}H - 7.26 ppm and ^{13}C - 77.16 ppm) and coupling constants (*J*) given in Hertz. Multiplicities are abbreviated as: b (broad), s (singlet), d (doublet), t (triplet), q (quartet), m (multiplet) or combinations thereof. High-resolution mass spectrometry (HRMS) spectra were recorded on a Waters LCT Premier mass spectrometer. Calculated and exact m/z values are reported in Daltons.

General procedures

General procedure A[1]: To a solution of the corresponding phenol (1.0 mmol) in CH_3CN (3 mL), anhydrous $Cu(OAc)_2$ (1.2 mmol) was added. The mixture was purged under vacuum and O_2 was introduced in the flask. Tetravinyltin (1.2 mmol) was then added and the reaction was allowed to stir at room temperature overnight. After completion, the mixture was poured into an aqueous 25 % NH_4OAc solution (25 mL) and stirred for 10 mins. Then, the aqueous phase was extracted with EtOAc three times and the combined organic layers were washed by brine, dried over anhydrous $MgSO_4$ and concentrated. Finally, the residue was purified by flash chromatography.

General procedure B: A solution of the corresponding vinyl ether (1.0 mmol) and 3,6-di-2-pyridyl-1,2,4,5-tetrazine (2.0 mmol) in CH_2Cl_2 (10 mL) was stirred at room temperature. After completion, the reaction mixture was concentrated and the crude product was purified by flash chromatography.

Synthesis of vinyl ethers

Synthesis of compound 1b:

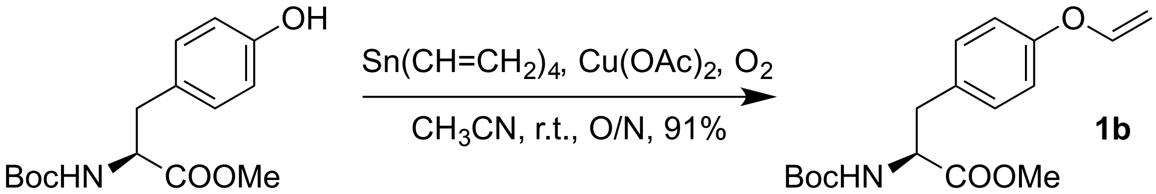

Following the general procedure A, compound 1b (292 mg, 0.91 mmol, 91%) was obtained from N-(tert-butoxycarbonyl)-L-tyrosine methyl ester (321 mg, 1 mmol), $Cu(OAc)_2$ (218 mg, 1.2 mmol) and tetravinyltin (218 uL, 1.2 mmol). The residue was purified by flash chromathography (petroleum ether/ethyl acetate 1:0 to 4:1) ^{1}H NMR (400 MHz, $CDCl_3$) δ 7.10 – 7.04 (m, 2H), 6.97 – 6.90 (m, 2H), 6.61 (dd, *J* = 13.7, 6.1 Hz, 1H), 4.97 (d, *J* = 8.3 Hz, 1H), 4.74 (dd, *J* = 13.7, 1.6 Hz, 1H), 4.64 – 4.49 (m, 1H), 4.42 (dd, *J* = 6.1, 1.7 Hz, 1H), 3.71 (s, 3H), 3.08 (dd, *J* = 13.8, 5.8 Hz, 1H), 3.00 (dd, *J* = 13.8, 6.1 Hz, 1H) 1.41 (s, 9H). ^{13}C NMR (126 MHz, $CDCl_3$) δ 172.4, 156.0, 155.2, 148.2, 130.8, 130.6, 117.2, 95.2, 80.1, 54.6, 52.4, 37.7, 28.4. HRMS (ESI) m/z calcd for $C_{14}H_{17}O_5N$ [M+H$^+$]: 280.1185, found: 280.1190

Synthesis of compound 1c[2]:

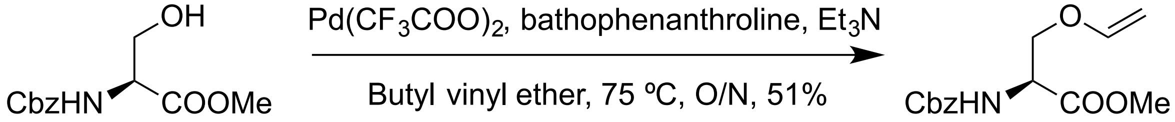

1c

To a solution of palladium (II) trifluoroacetate (1.6 mg, 0.005mmol) and bathophenanthroline (1.6 mg, 0.005 mmol) in n-butyl vinyl ether (2.6 mL) *N*-carbobenzoxy-L-serine methyl ester (253 mg, 1 mmol) and triethylamine (10 μL, 0.075 mmol) were added and the reaction was stirred at 75 ºC overnight. Then, the solvent was removed and the residue purified by column chromatography (petroleum ether/ethyl acetate 1:0 to 4:1) to afford compound 1c (141 mg, 0.51 mmol, 51%).^{1}H NMR (500 MHz, $CDCl_3$) δ 7.42 – 7.27 (m, 5H), 6.41 (dd, *J* = 14.3, 6.8 Hz, 1H), 5.62 (d, *J* = 8.8 Hz, 1H), 5.13 (s, 2H), 4.62 (dt, *J* = 8.7, 3.2 Hz, 1H), 4.20 (dd, *J* = 14.3, 2.6 Hz, 1H), 4.12 (dd, *J* = 9.9, 3.1 Hz, 1H), 4.06 (dd, *J* = 6.8, 2.5 Hz, 1H), 3.95 (dd, *J* = 9.9, 3.2 Hz, 1H), 3.78 (s, 3H). ^{13}C NMR (126 MHz, $CDCl_3$) δ 170.3, 156.0, 151.2, 136.2, 128.7, 128.4, 128.2, 87.8, 67.8,

67.3, 53.8, 52.9. HRMS (ESI) m/z calcd for $C_{14}H_{17}O_5N$ [M+H$^+$]: 280.1185, found: 280.1190.

Synthesis of compound 1d:

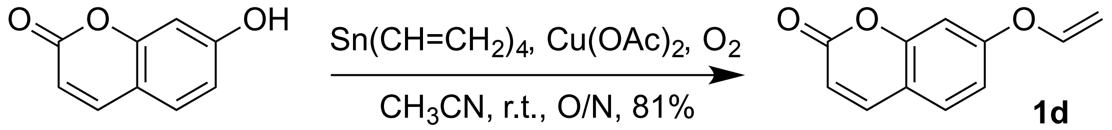

Following the general procedure A, compound 1d (153 mg, 0.814 mmol, 81%) was obtained from umbelliferone (162 mg, 1 mmol), $Cu(OAc)_2$ (218 mg, 1.2 mmol) and tetravinyltin (218 uL, 1.2 mmol). The residue was purified by flash chromathography (pethroleum ether/ethyl acetate 1:0 to 4:1). ^{1}H NMR (400 MHz, $CDCl_3$) δ 7.65 (d, J = 9.5 Hz, 1H), 7.42 (d, J = 8.9 Hz, 1H), 6.96 – 6.88 (m, 2H), 6.65 (dd, J = 13.6, 6.0 Hz, 1H), 6.29 (d, J = 9.5 Hz, 1H), 4.93 (dd, J = 13.6, 1.9 Hz, 1H), 4.63 (dd, J = 6.0, 1.9 Hz, 1H). ^{13}C NMR (101 MHz, $CDCl_3$) δ 160.8, 159.7, 155.6, 146.4, 143.2, 129.2, 114.4, 114.3, 113.8, 104.2, 98.4. HRMS (ESI) m/z calcd for $C_{11}H_9O_3$ [M+H$^+$]: 189.0546, found: 189.0541

Synthesis of compound 1e:

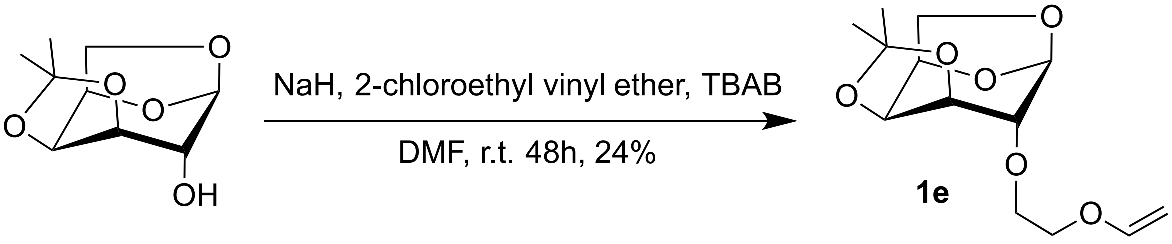

To a solution of 1,6-anhydro-3,4-di-O-isopropylidene-β-D-galactopyranose (100 mg, 0.495 mmol) in anhydrous DMF (5 mL) at 0 °C under inert atmosphere, sodium hydride (60% in mineral oil, 82.8 mg, 2.48 mmol) was added. After 30 min, 2-chloroethyl-vinylether (252 µL, 2.48 mmol) and tetrabutylammonium bromide (40.5 mg, 0.084 mmol) were added and the reaction was stirred for two days at room temperature. Then, 4 mL of water were added carefully and the mixture was concentrated under reduced pressure. The residue was dissolved in CH_2Cl_2, washed with brine three times, dried over anhydrous $MgSO_4$ and concentrated under vacuum. The residue was purified by column chromatography (petroleum ether/ethyl acetate 2:1 to 0:1) to afford compound 1e (32 mg, 0.118 mmol, 24%). ^{1}H-NMR (500 MHz, $CDCl_3$): δ = 6.48 (dd, J = 14.3, 6.8 Hz, 1H, C*H*=CH_2), 5.45 (s, 1H, H1), 4.50 (t, J= 5.7 Hz, 1H, H5), 4.45 (t, J= 6.6 Hz, 1H, H4), 4.22 –

4.19 (m, 2H, H3, CH=CH_2), 4.08 (dd, *J* = 7.6, 1.0 Hz, 1H, H6a), 4.03 (dd, *J* = 6.8, 2.2 Hz, 1H, CH=CH_2), 3.89 – 3.81 (m, 4H, CH_2-CH_2), 3.58 (dd, J = 7.4, 5.4 Hz, 1H, H6b), 3.55 – 3.54 (m, 1H, H2), 1.543 (s, 3H, CH_3), 1.35 (s, 3H, CH_3) ppm. ^{13}C-NMR (126 MHz, $CDCl_3$): δ = 151.8 (*C*H=CH_2), 108.7 (*C*(CH_3)$_2$), 99.8 (C1), 87.0 (CH=*C*H_2), 78.9 (*C2*), 74.2 (C3), 72.2 (C5), 69.4 (C4), 69.1 (*C*H_2-CH_2), 67.3 (*C*H_2-CH_2), 63.2 (C6), 25.9 (CH_3), 24.49 (CH_3) ppm. HRMS (ESI) m/z calcd for $C_{13}H_{20}O_3Na$ [M+Na$^+$]: 295.1152, found: 295.1149.

Synthesis of compound 5:

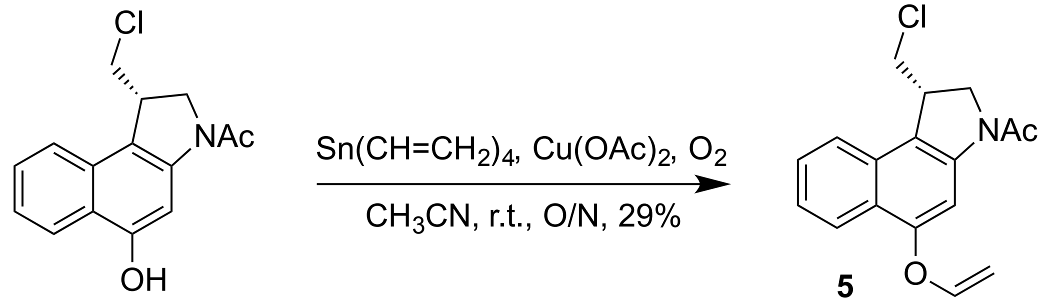

Following the general procedure A, compound 5 (33 mg, 0.120 mmol, 29%) was obtained from (S)-1-(1-(chloromethyl)-5-hydroxy-1,2-dihydro-3H-benzo[e]indol-3-yl)ethan-1-one[3] (114 mg, 0.415 mmol), $Cu(OAc)_2$ (90 mg, 0.497 mmol) and tetravinyltin (90 uL, 0.497 mmol). The residue was purified by flash chromathography (petroleum ether/ethyl acetate 7:3). ^{1}H NMR (400 MHz, $CDCl_3$) δ 8.22 (d, *J* = 8.4 Hz, 1H), 8.16 (s, 1H), 7.67 (d, *J* = 8.3 Hz, 1H), 7.54 (t, *J* = 7.4 Hz, 1H), 7.41 (t, *J* = 7.5 Hz, 1H), 6.84 (dd, *J* = 13.6, 6.0 Hz, 1H), 4.94 (dd, *J* = 13.6, 1.7 Hz, 1H), 4.59 (dd, *J* = 5.9, 1.7 Hz, 1H), 4.32 – 4.20 (m, 2H), 4.05 (ddt, *J* = 10.6, 7.3, 3.5 Hz, 1H), 3.95 (dd, *J* = 11.3, 3.3 Hz, 1H), 3.45 (t, *J* = 10.8 Hz, 1H), 2.31 (s, 3H). ^{13}C NMR (101 MHz, $CDCl_3$) δ 169.2, 154.2, 148.0, 141.5, 130.0, 128.0, 124.3, 123.5, 123.2, 122.2, 117.7, 102.4, 96.7, 54.1, 46.3, 42.5, 24.5. HRMS (ESI) m/z calcd for $C_{17}H_{17}ClNO_2$ [M+H$^+$]: 302.0942, found: 302.0946

Decaging reactions

Decaging of compound 1a:

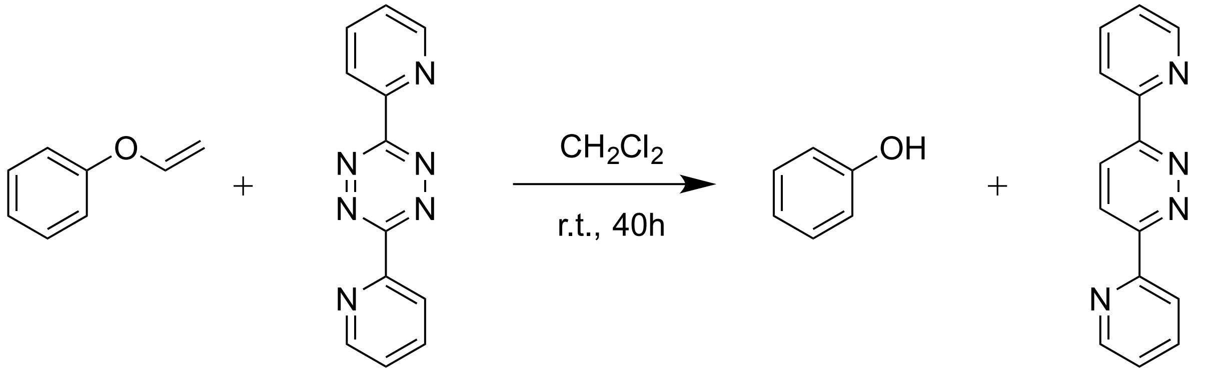

1a **2a** **3a** 61% **4a** 49%

Following the general procedure B, phenyl vinyl ether 1a (20 μL, 0.166 mmol) and 3,6-di-2-pyridyl-1,2,4,5-tetrazine 2a (78 mg, 0.499 mmol) were stirred in CH_2Cl_2 (1.6 mL) for 40 h. The residue was purified by flash chromatography (petroleum ether/ethyl acetate 9:1) to afford phenol 3a (9.5 mg, 0.101 mmol, 61%) and 3,6-di(pyridin-2-yl)pyridazine 4a (19 mg, 0.081 mmol, 49%). Spectral data were in agreement with the previously reported in the literature.[4] ^{1}H NMR for 4a (500 MHz, $CDCl_3$) δ 8.81 – 8.71 (m, 4H), 8.69 (s, 2H), 7.91 (td, J = 7.8, 1.8 Hz, 2H), 7.41 (ddd, J = 7.5, 4.8, 1.2 Hz, 2H). ^{13}C NMR (126 MHz, $CDCl_3$) δ 158.3, 153.6, 149.6, 137.4, 125.3, 125.0, 121.9. HRMS (ESI) m/z calcd for $C_{14}H_{11}N_4$ [M+H^{+}]: 235.0984, found 235.0984

Decaging of compound 1b:

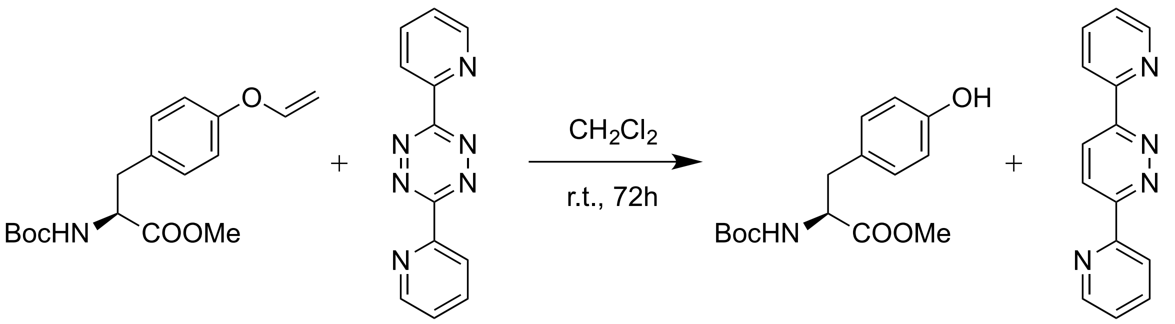

1b **2a** **3b** 68% **4a** 65%

Following the general procedure B, O-vinyl-*N*-(*tert*-butoxycarbonyl)-L-tyrosine methyl

ester 1b (40 mg, 0.125 mmol) and 3,6-di-2-pyridyl-1,2,4,5-tetrazine 2a (59 mg, 0.25 mmol) were stirred in CH_2Cl_2 (1.25 mL) for 72 h. The residue was purified by flash chromatography (petroleum ether/ethyl acetate 9:1 to 8:2) to afford starting material O-vinyl- *N*-(*tert*-butoxycarbonyl)-L-tyrosine methyl ester 1b (11 mg, 0.034 mmol, 27%), 3,6-di(pyridin-2-yl)pyridazine 4a (19 mg, 0.081 mmol, 65%) and *N*-(*tert*-butoxycarbonyl)-L-tyrosine methyl ester 3b (25 mg, 0.085 mg, 68%). 1H NMR for 3b (400 MHz, $CDCl_3$) δ 6.97 (d, *J* = 8.4 Hz, 2H), 6.73 (d, J = 8.4 Hz, 2H), 4.99 (d, J = 8.4 Hz, 1H), 4.54 (m, 1H), 3.71 (s, 3H), 3.03 (dd, *J* = 13.9, 5.7 Hz, 1H), 2.96 (dd, *J* = 14.1, 6.1 Hz, 1H) 1.42 (s, 9H). ^{13}C NMR (101 MHz, $CDCl_3$) δ 172.7, 155.4, 155.0, 130.5, 127.9, 115.6, 80.3, 54.7, 52.4, 37.7, 28.4. HRMS (ESI) m/z calcd for $C_{15}H_{20}NO_5$ [M-H$^-$]: 294.1347, found: 294.1346.

Decaging of compound 1c:

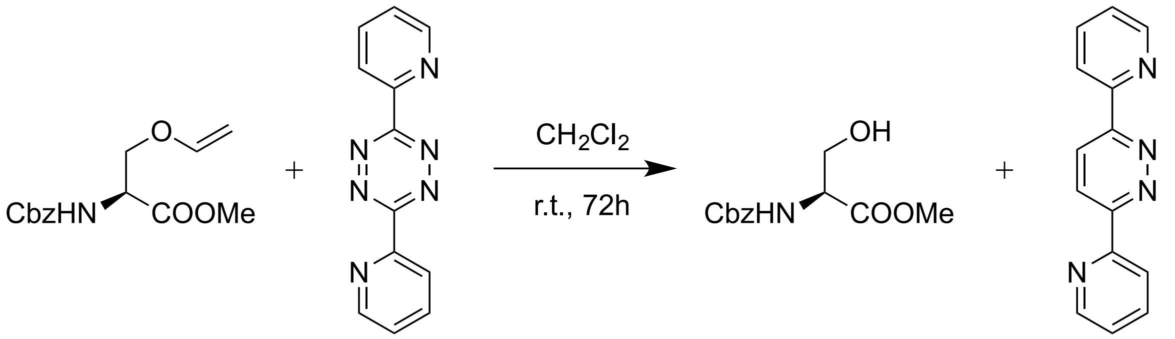

1c **2a** **3c** 57% **4a** 72%

Following the general procedure B, O-vinyl-*N*-carbobenzoxy-L-serine methyl ester 1c (25 mg, 0.089 mmol) and 3,6-di-2-pyridyl-1,2,4,5-tetrazine 2a (42 mg, 0.179 mmol) were stirred in CH_2Cl_2 (0.9 mL) for 72 h. The residue was purified by flash chromatography (petroleum ether/ethyl acetate 9:1 to 8:2) to afford *N*-carbobenzoxy-L-serine methyl ester 3c (13 mg, 0.051 mmol, 57%) and 3,6-di(pyridin-2-yl)pyridazine 4a (15 mg, 0.064 mmol, 72%). ^1H NMR for 3c (400 MHz, $CDCl_3$) δ 7.41 – 7.29 (m, 5H), 5.71 (d, *J* = 7.7 Hz, 1H), 5.13 (s, 2H), 4.50 – 4.40 (m, 1H), 4.01 (dd, *J* = 11.2, 3.7 Hz, 1H), 3.93 (dd, *J* = 11.3, 3.5 Hz, 1H), 3.79 (s, 3H). ^{13}C NMR (101 MHz, $CDCl_3$) δ 171.0, 156.3, 136.2, 128.7, 128.4, 128.3, 67.4, 63.5, 56.2, 52.9. HRMS (ESI) m/z calcd for $C_{12}H_{15}NO_5Na$ [M+Na$^+$]: 276.0848, found: 276.0851

Decaging of compound 1d:

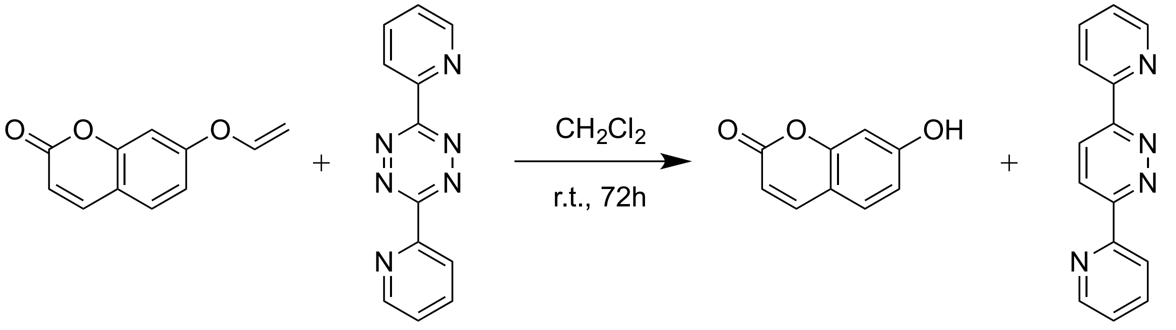

1d **2a** **3d** 47% **4a** 50%

Following the general procedure B, 7-(vinyloxy)-2*H*-chromen-2-one 1d (30 mg, 0.160 mmol) and 3,6-di-2-pyridyl-1,2,4,5-tetrazine (75 mg, 0.319 mmol) were stirred in CH_2Cl_2 (1.6 mL) for 72 h. The residue was purified by flash chromatography (petroleum ether/ethyl acetate 9:1 to 8:2) to afford starting material 7-(vinyloxy)-2*H*-chromen-2-one 1d (13 mg, 0.07 mmol, 44%), 3,6-di(pyridin-2-yl)pyridazine 4a (17 mg, 0.075 mmol, 47%) and . 7-hydroxy-2H-chromen-2-one 3d (13 mg, 0.080 mg, 50%). 1H NMR for 3d (500 MHz, MeOD) δ 7.84 (d, J = 9.4 Hz, 1H), 7.44 (d, J = 8.5 Hz, 1H), 6.79 (dd, J = 8.5, 2.3 Hz, 1H), 6.70 (d, J = 2.2 Hz, 1H), 6.18 (d, J = 9.5 Hz, 1H). ^{13}C NMR (126 MHz, MeOD) δ 163.7, 163.1, 157.2, 146.1, 130.7, 114.5, 113.1, 112.3, 103.4. HRMS (ESI) m/z calcd for $C_9H_6O_3Na$ [M+Na$^+$]: 185.0209, found: 185.0201.

Decaging of compound 1e:

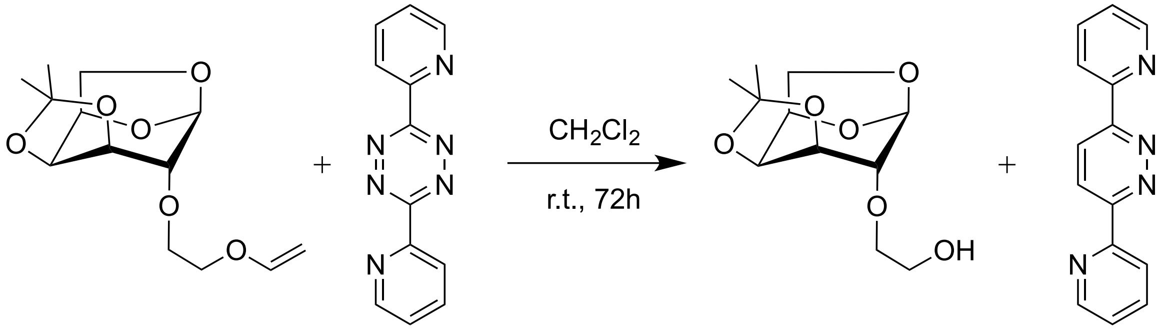

1e **2a** **3e** 65% **4a** 50%

Following the general procedure B, compound 1e (13 mg, 0.048 mmol) and 3,6-di-2-pyridyl-1,2,4,5-tetrazine 2a (23 mg, 0.096 mmol) were stirred in CH_2Cl_2 (1 mL) for 72 h. The residue was purified by flash chromatography (petroleum ether/ethyl acetate 1:2) to afford compound 3e (7.7 mg, 0.031 mmol, 65%) and 3,6-di(pyridin-2-yl)pyridazine 4a (5.6

mg, 0.024 mmol, 50%).^{1}H-NMR (400 MHz, $CDCl_3$) δ = 5.45 (s, 1H, H1), 4.52 (t, J = 5.7 Hz, 1H, H5), 4.45 (t, J= 6.7 Hz, 1H, H4), 4.20 (d, J = 7.3 Hz, 1H, H3), 4.09 (d, J = 7.6 Hz, 1H, H6a), 3.82 – 3.67 (m, 4H, CH_2-CH_2), 3.59 (dd, J = 7.6, 5.3 Hz, 1H, H6b), 3.52 (s, 1H, H2), 1.53 (s, 3H, CH_3), 1.36 (s, 3H, CH_3) ppm. ^{13}C-NMR (101 MHz, $CDCl_3$) δ = 108.8 ($C(CH_3)_2$), 99.7 (C1), 78.7 (C2), 74.0 (C3), 72.2 (C5), 71.6 (CH_2-CH_2), 69.4 (C4), 63.2 (C6), 61.9 (CH_2-CH_2), 25.9 (CH_3), 24.5 (CH_3) ppm. HRMS (ESI) m/z calcd for $C_{11}H_{18}O_6Na$ [M+Na$^+$]: 269.1001, found: 269.1160

Synthesis of tetrazine 2c

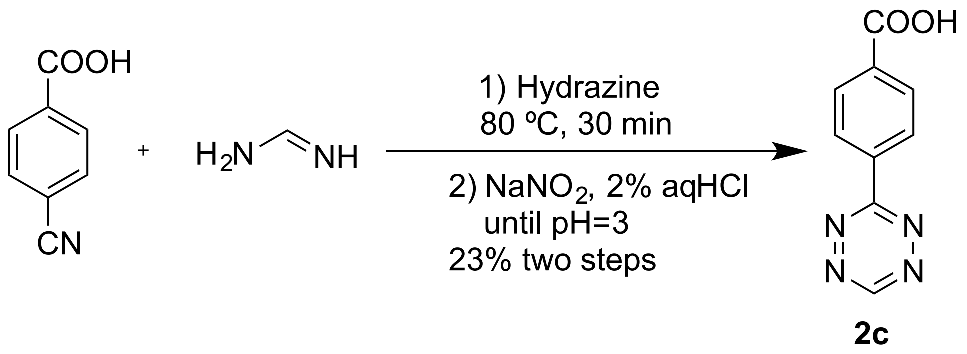

Hydrazine hydrate (4 mL) was added under inert atmosphere dropwise to a mixture of 4-cianobenzoic acid (600 mg, 4 mmol) and formamidine acetate (2122 mg, 20.4 mmol). The reaction was then stirred at 80 C for 30 min and allowed to cool to room temperature. Sodium nitrite (1408 mg, 20.4 mmol) in water (4 mL) was added to the reaction mixture followed by the dropwise addition of of 2% aqueous HCl until the pH of the solution was approximately 3 and a pink precipate was formed.The precipitate was then filtered and washed with 2% aqueous HCl. The crude was finally purified by flash chromatography (CH_2Cl_2/CH_3OH 2% to 10%) to afford compound 2c (186 mg, 0.92 mmol, 23%) ^{1}H NMR (400 MHz, DMSO-d_6) δ 10.65 (s, 1H), 8.66 – 8.55 (m, 2H), 8.27 – 8.17 (m, 2H). ^{13}C NMR (101 MHz, DMSO) δ 166.8, 165.1, 158.3, 135.6, 134.7, 130.2, 128.0. HRMS (ESI) m/z calcd for $C_9H_7N_4O_2$ [M+H$^+$]: 203.0569, found: 203.0567

2. DETERMINATION OF KINETIC RATE CONSTANTS:

Rate constants were determined under pseudo-first order conditions. Stocks solutions of 10% H_2O in DMF were prepared for each tetrazine (2 mM), compound **1a** (300 to 700 mM) and 5-norbonen-2-ol (40 to 200 mM). In a similar way, stock solutions in 40 or 60% H_2O/DMF were prepared for **1a** (150 mM to 350 mM) and tetrazine **2c** (2 mM). Mixing equal volumes of the stocks solutions resulted in final concentrations of 1 mM of the tetrazine and 150 to 350 mM of the phenyl vinyl ether 1a in 10% H_2O/DMF, 75 to 175 mM in 40 or 60% H_2O/DMF or 20 to 100 mM of the 5-norbonen-2-ol. The decay of the UV absorption of the tetrazine at the corresponding wavelength was followed over time at 37ºC with a Cari 100 instrument from Varian and the data were fit to a single exponential decay equation. Each measurement was carried out three different times. The resulted values for k_{obs} were then plotted against the coumarin vinyl ether concentrations and fitted to a linear equation to obtain the second order constant k from the slop. The results obtained in 60% H_2O/DMF were not fully reliable due to the poor solubility of the compounds in this solvent system.

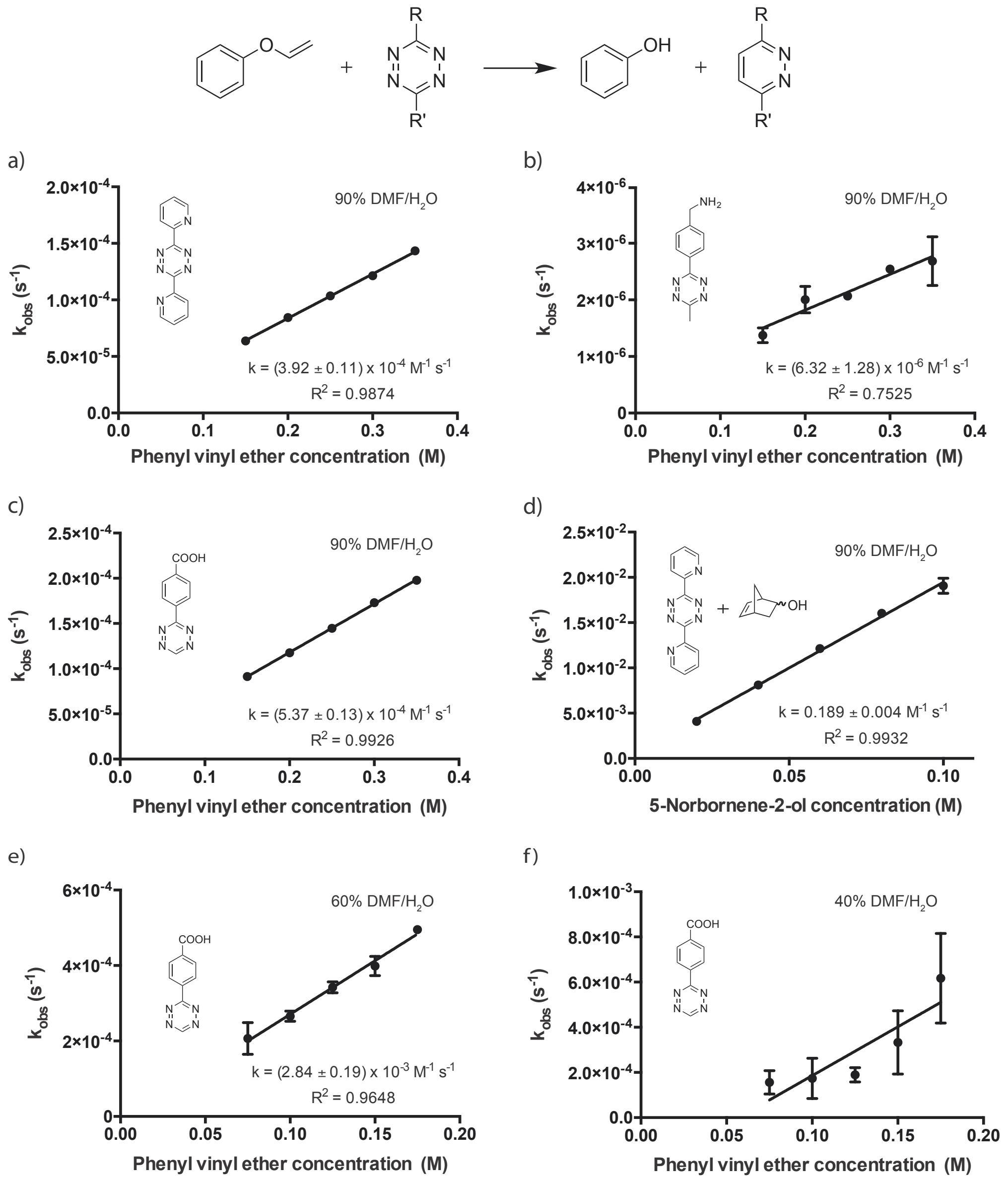

Figure S1: Rate constants k obtained from plots of observed rate k_{obs} versus compound **1a** or 5-norbornene-2-ol concentration for various tetrazines (**2a**–**2c**).

3. STABILITY STUDIES

The assesment of the stability of the compounds **1b**–**d** and 5 in PBS was performed at 37ºC. Stock solutions in acetonitrile (20 mM for compounds **1b**–**d** and 10 mM for drug) were diluted in PBS to a final concentration of 200 μM for compounds **1b**–**d** and 100 μM for compound **5**. Acetophenone was used as internal stardard. Stock solution of

acetophenone in acetonitrile (20 mM) was added to the samples to a final concentration of 200 μM. The solutions were kept at 37 ºC and analyzed at different times by HPLC/UV analysis. The assesment of the stability of compound **1a** was performed with a solution 20 mM in 10% H_2O/DMF at 37ºC. This solution was diluted to 200 μM with H_2O at different times and analyzed by HPLC/UV. The stability of the compound **5** was also assessed with solutions 200 μM in 50% DMF/H_2O and DMF. These solutions were analyzed at different times by HPLC/UV. The stability of compound **1e** could not be performed due to its lack of UV absorbance. The HPLC/UV analysis was performed on a Agilent 1100 series instrument with μBondapak C18 column (125Å, 10 μm, 3.9 mm X 150 mm). The mobile phase consisted of acetonitrile and water. The samples were eluted with a linear gradient (13 minutes 0-50% ACN and 1.5 min 50-100% ACN, 0.1% TFA, 0.75 mL/mmol). Retention times: **acetophenone**: 5.137-5.576; **1a**: 5.809-5.879; **1b**: 9.863-10.219; **1c**: 6.904-7.206; **1d**: 7.041-7.220; **5**: 9.501-10.293.

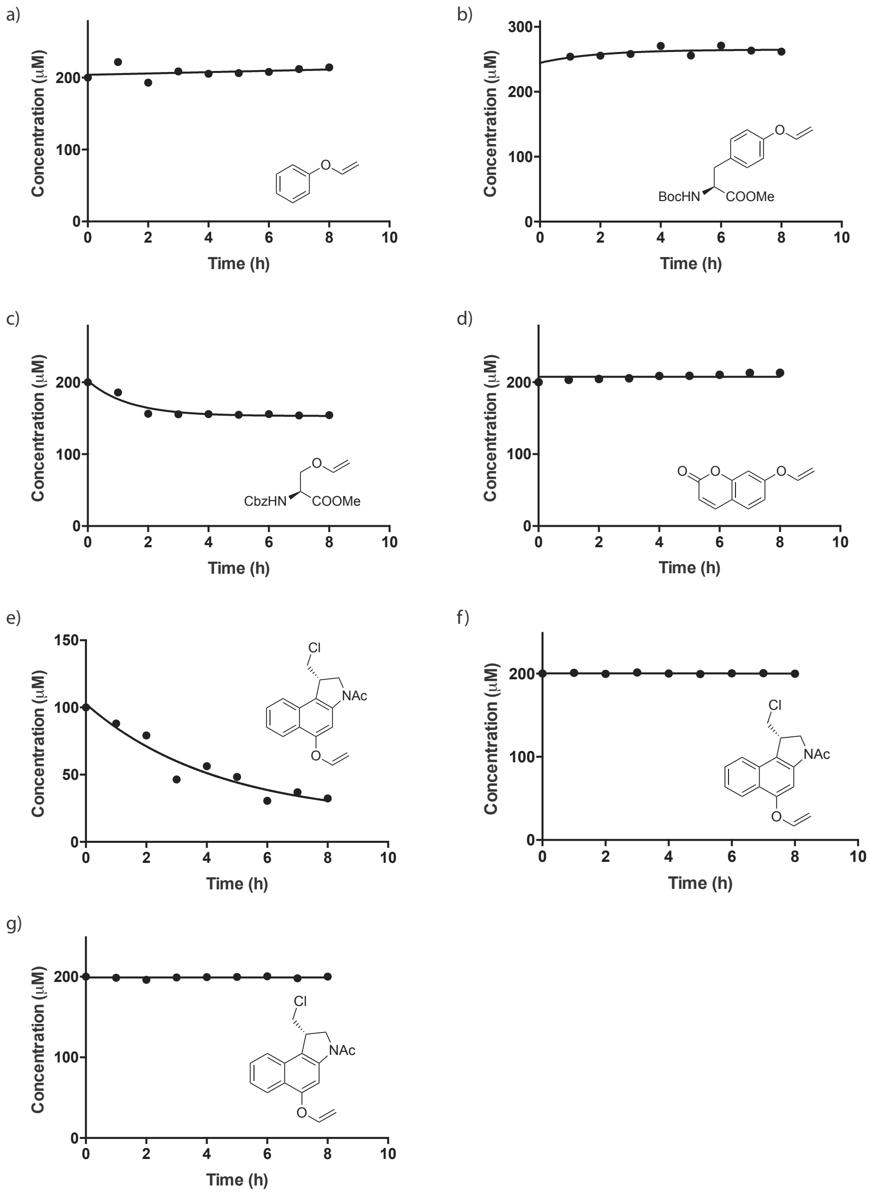

Figure S2: Stability studies performed at 37 ºC and analyzed at different times by HPLC/UV; a) the stability of compound **1a** was assessed with a solution 20 mM in 10% H_2O/DMF; b-d) the stability of compounds **1b–d** was assessed with solutions 200 μM in PBS; e) the stability of compound **5** was assessed with a solution 100 μM in PBS; f) the stability of compound **5** was assessed with a solution 200 μM in 50% H_2O/DMF; g) the stability of compound **5** was assessed with a solution 200 μM in DMF.

4. RELEASE STUDIES BY ^{1}H-NMR

Stock solutions of 1a (81.4 mM, CD_3CN), tetrazine 2a (7.3 mM CD_3CN) and dimethylformamide (129 mM CD_3CN, internal reference) were diluted in a mixture 10% D_2O in CD_3CN to final concentrations of 3 mM for 1a and 2a and 2 mM for DMF. The reaction was allowed to proceed at room temperature and was monitorized by ^{1}H NMR at different times.

5. RELEASE STUDIES OF COMPOUND 5 BY HPLC:

Compound **5** was diluted in PBS pH=7.4 to a final concentration of 100 μM from 10 mM stock in acetonitrile. Then tetrazine 4c was added to a final concentration of 500 μM from a 50 mM stock in DMSO. The reaction was performed at 37 C and monitorized by HPLC/UV detection at different times. The HPLC/UV analysis was performed on a Agilent 1100 series instrument with μBondapak C18 column (125Å, 10 μm, 3.9 mm X 150 mm). The mobile phase consisted of acetonitrile and water. The samples were eluted with a linear gradient (13 minutes 0-50% ACN and 1.5 min 50-100% ACN, 0.1% TFA, 0.75 mL/mmol). Retention times: **2c**: 4.455-4.692; **4c**: 5.275-5.375; **5**: 9.963-10.230; **6**: 7.851-8.064; **7**: 7.263-7.329.

HPLC at 0h:

Print of window 38: Current Chromatogram(s)

Current Chromatogram(s)

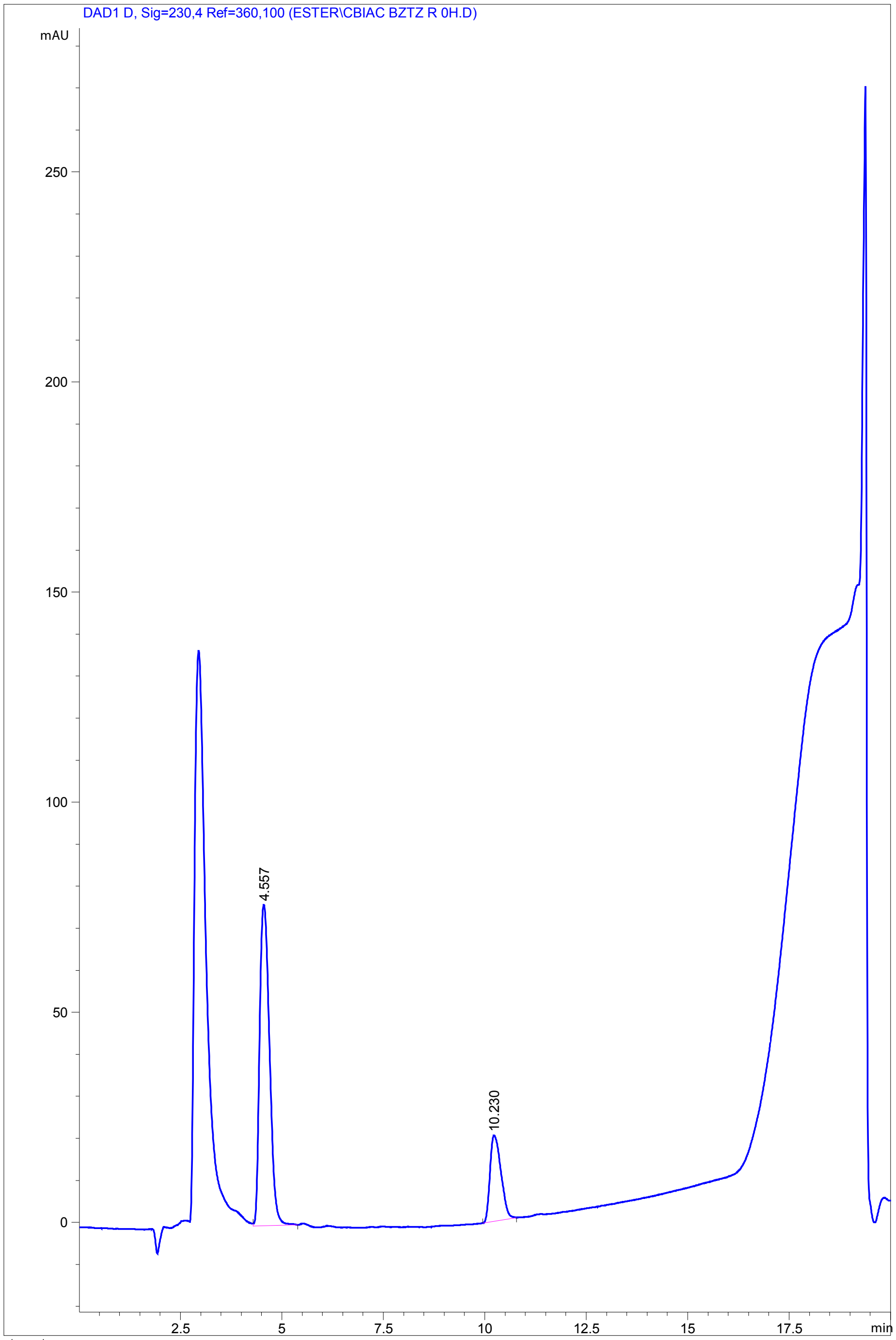

HPLC 30/08/2016 18:44:06 SYSTEM Page 1 of 1

HPLC at 1.5h:

Print of window 38: Current Chromatogram(s)

Current Chromatogram(s)

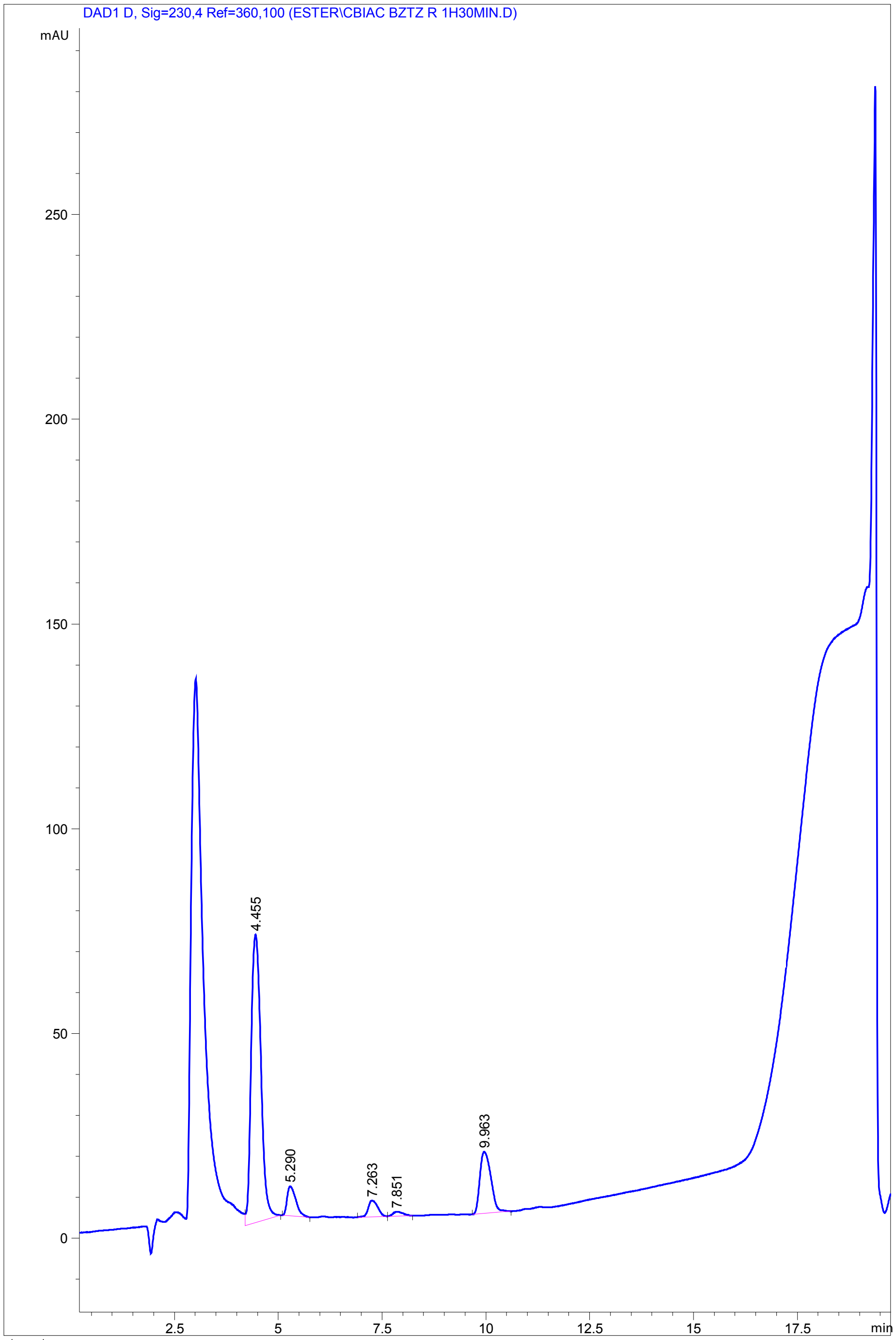

HPLC 30/08/2016 17:07:48 SYSTEM Page 1 of 1

HPLC at 3h:

Print of window 38: Current Chromatogram(s)

Current Chromatogram(s)

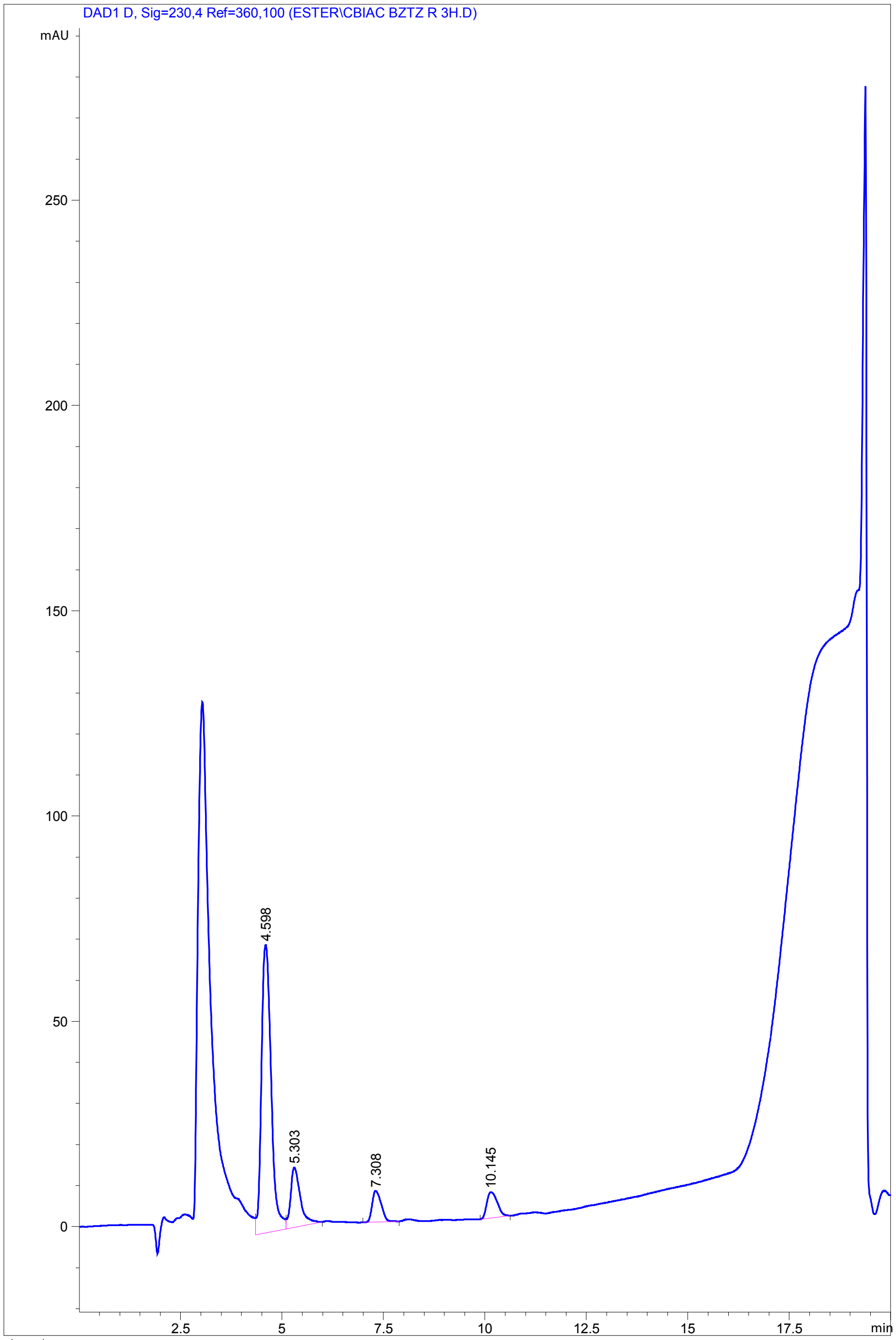

HPLC 30/08/2016 17:11:33 SYSTEM Page 1 of 1

*LC/MS for Compound **4c**:* m/z for C11H7N4O2 [M-H$^-$] calc: 199.1, found: 199.3

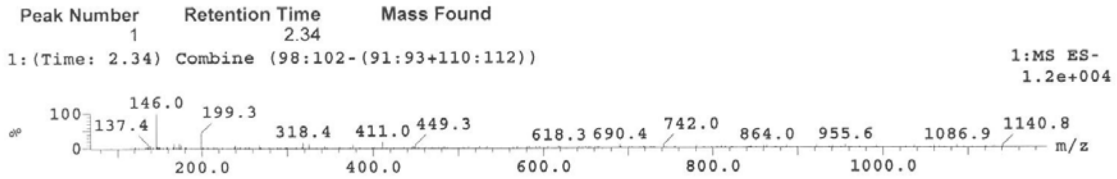

*LC/MS for Compound **2c**:* m/z for C9H5N4O2 [M-H$^-$] calc: 201.0, found: 201.3

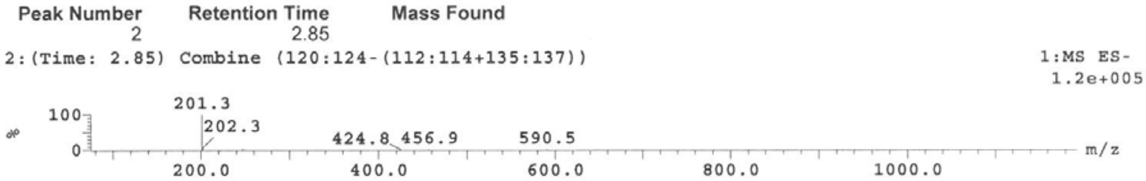

LC/Ms for compound 7: m/z for C15H14NO2 [M+H+]: calc: 240.1, found: 240.2

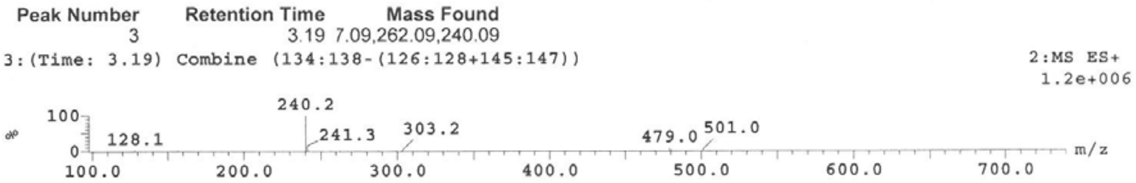

HPLC at 7h:

Print of window 38: Current Chromatogram(s)

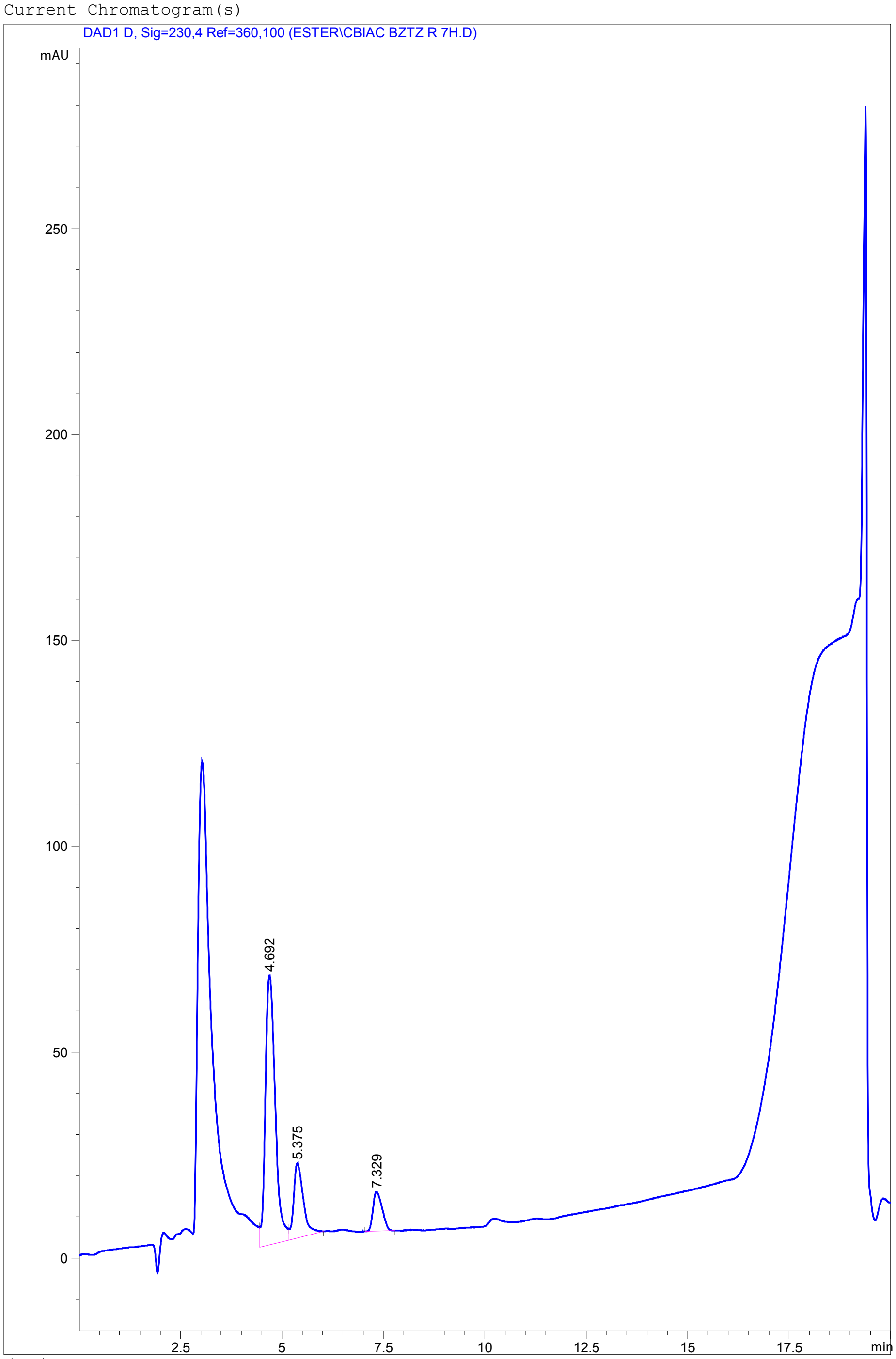

HPLC 30/08/2016 18:24:13 SYSTEM Page 1 of 1

6. **TABLE S1:** Examples of FDA approved alcohol-containing drugs (cf DrugBank v5.0).

Structure	Name
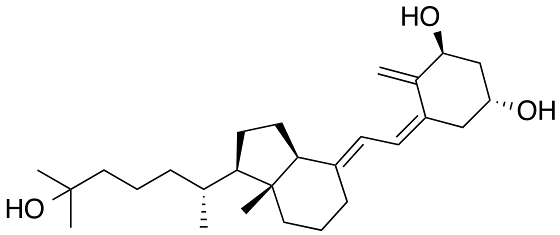	Calcitriol
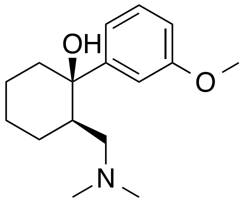	Tramadol
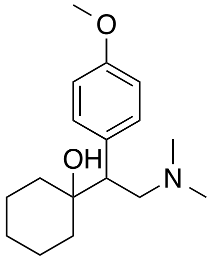	Venlafaxine
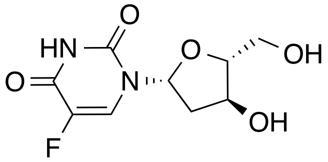	Floxuridine
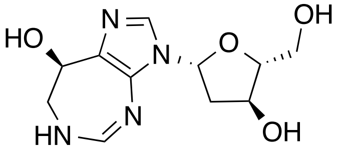	Pentostatin
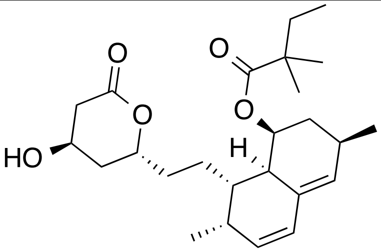	Simvastatin

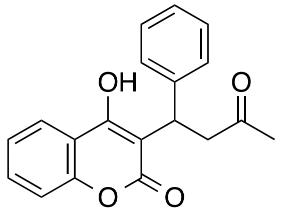	Warfarin
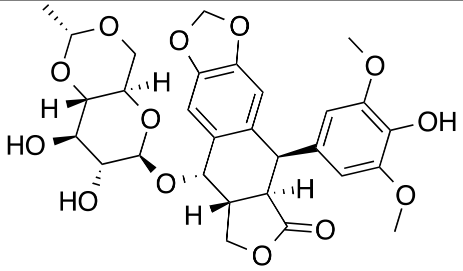	Etoposide
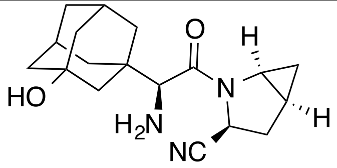	Saxagliptin
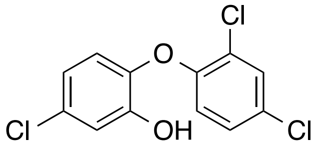	Triclosan

7. IN VIVO DECAGING METHODS

Cell culture

A549 (ATCC® CCL-185™) and HepG2 (ATCC® HB-8065™) cells were routinely grown in a humidified incubator at 37 ºC under 5% CO_2, and split before reaching confluence using TrypLE™ Express. Both cell lines were grown on DMEM medium supplemented with 10% heat-inactivated FBS, 2 mM GlutaMAX™, 10 mM HEPES, 1% NEAA, 1 mM sodium pyruvate, 100 units/mL penicillin and 100 µg/mL streptomycin. All reagents were bought from Gibco, Life Technologies (USA), unless otherwise stated.

Cytotoxicity and estimation of IC_{50}s in HepG2 and A549 cells

Cytotoxicity of coumarin **1d**, tetrazine **2c**, double prodrug **5** and drug **7** was assessed using a CellTiter-Blue® Cell Viability Assay (Promega, USA), a fluorescent dye approach based on the ability of metabolically active cells to covert the dye resazurin to the fluorescent resorufin product. Briefly, cells were seeded at a concentration of 10 000 cells/well (100

μL) in flat-bottom 96 well-plates and allowed to adhere and adapt to the plates for 24 h. At this point, culture medium was exchanged to complete medium supplemented with increasing concentrations of each compound in technical triplicates (for coumarin **1d**: 1, 10, 25, 50, 100, 150 and 200 μM; for other compounds: 0.01, 0.1, 0.5, 1, 10, 25 and 50 μM). Plates were incubated for 46 h 30 min, at which time cell viability was assessed by exchanging the culture medium to medium supplemented with CellTiter-Blue Reagent (dilution 1:20 from commercial stock) and incubated for another 1 h 30 min, before analysis of fluorescence on an Infinite M200 (Tecan, USA) plate-reader (λ_{exc}=530, λ_{em}=590). Relative fluorescence units (R.L.U.) were normalized to the values obtained for the appropriate vehicle controls. Results are shown as average of 3 independent experiments. A sigmoidal curve (variable slope) was fitted to each dataset, using GraphPad Prism v5 software, and used to calculate the half maximal inhibitory concentration (IC_{50}) for each compound on both cell-lines.

Detection of decaging of coumarin by tetrazine 2c inside HepG2 cells by confocal microscopy

Cells were seeded at a concentration of 30 000 cells/well (300 μl) in 8 well IbidiTreat microslides (Ibidi, USA) and allowed to adhere and adapt to the plates for 24 h. At this point, culture medium was exchanged to complete medium supplemented with coumarin **1d** at 25 μM, and cells were incubated for 5 h. Medium was then exchanged to medium supplemented with tetrazine **2c** 10 μM, and cells were incubated for another 4 h. Cells were washed with PBS pH 7.4 and incubated at RT for 15 min with 2.5 μM Syto61 (LifeTechnologies, USA), before being washed again with PBS pH 7.4, and the medium was replaced with complete medium without phenol-red. Cells were observed using a LSM710 confocal microscope (Zeiss, Germany), equipped with a Diode 405/30 nm (decaged, fluorescent coumarin) and a HeNe633 nm (Syto61) laser units. Fluorescence was detected at 409-565 nm (decaged, fluorescent coumarin) and at 638–797 nm (Syto61) and representative pictures were taken within 1 h after removal of Syto61. Images were then processed using ImageJ 1.49v software, to remove background noise (display range set to 30–255; 8 bit/pixel images) and overlay of the scale bar.

Detection of decaging of the prodrug by tetrazine 2c inside HepG2 and A549 cells by assessment of cytotoxicity

Cells were seeded as described above to assess cytotoxicity and estimate IC_{50}s in HepG2 and A549 cells and allowed to adapt to the plates for 24 h. Cells were incubated with increasing concentrations of double prodrug **5** or equivalent vehicle controls for another 24 h. The culture medium was then exchanged to complete medium supplemented with increasing concentrations of tetrazine **2c**, drug **7** or equivalent vehicle controls. Cells were incubated for another 46 h 30 min until proceeding with the CellTiter-Blue Cell Viability Assay (Promega, USA), as described above. Relative fluorescence units (R.L.U.) were normalized to the values obtained for the appropriate vehicle controls. Bars represent the average of 3 independent experiments and error bars represent SEM.

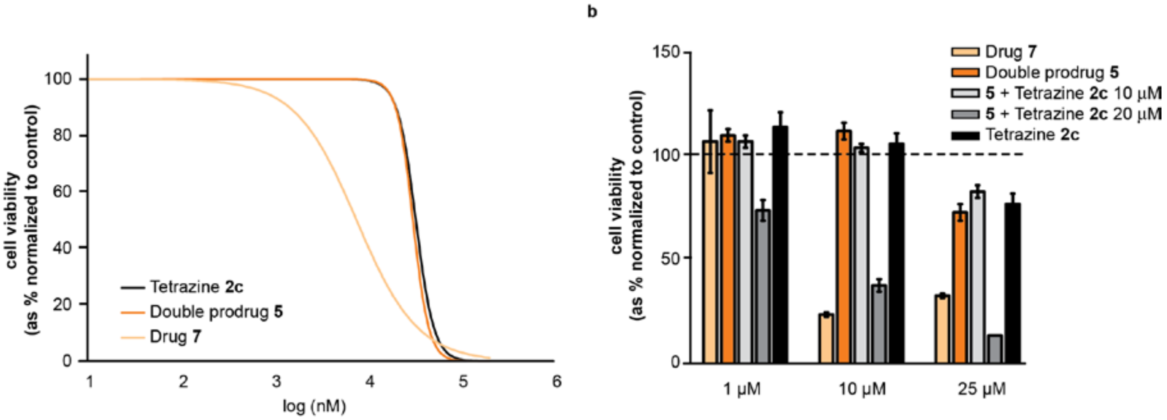

c

	IC_{50} (mM) average ± SEM	
Compound	A549 cells	HepG2 cells
Coumarin **1d**	$117.49 \pm 1.11 \times 10^{-3}$	$110.92 \pm 1.07 \times 10^{-3}$

Figure S3: a) Cytotoxicity dose-response curves of tetrazine **2c**, double prodrug **5** and drug **7** in HepG2 cells, obtained after 46.5 h of exposure. b) Cytotoxic effects of activation of the double prodrug **5** by tetrazine **2c** inside HepG2 cells. c) IC_{50} values of coumarin in A549 and HepG2 cells.

8. COMPUTATIONAL DETAILS.

Full geometry optimizations were carried out with Gaussian 09[5] using the M06-2X hybrid functional[6] and 6-31+G(d,p) basis set. Bulk solvent effects in water were considered implicitly through the IEF-PCM polarizable continuum model.[7] The possibility of different conformations was taken into account for all structures. Frequency analyses were carried out at the same level used in the geometry optimizations, and the nature of the stationary points was determined in each case according to the appropriate number of negative eigenvalues of the Hessian matrix. . The quasiharmonic approximation reported by Trular et al. was used to replace the harmonic oscillator approximation for the calculation of the vibrational contribution to enthalpy and entropy.[8] Scaled frequencies were not considered. Scaled frequencies were not considered. Mass-weighted intrinsic reaction coordinate (IRC) calculations were carried out by using the Gonzalez and Schlegel scheme[9] in order to ensure that the TSs indeed connected the appropriate reactants and products. Gibbs free energies (ΔG) were used for the discussion on the relative stabilities of the considered structures. Free energies calculated using the gas phase standard state concentration (1 atm = 1/24.5 M) were converted to reproduce the standard state concentration in solution (1 M) by adding or subtracting 1.89 kcal mol^{-1} for bimolecular additions and decompositions, respectively. Cartesian coordinates, electronic energies, entropies, enthalpies, Gibbs free energies, and lowest frequencies of the calculated structures are available below.

Figure S4: Minimum energy pathway (MEP) calculated with PCM(H_2O)/M06-2X/6-31+G(d,p)

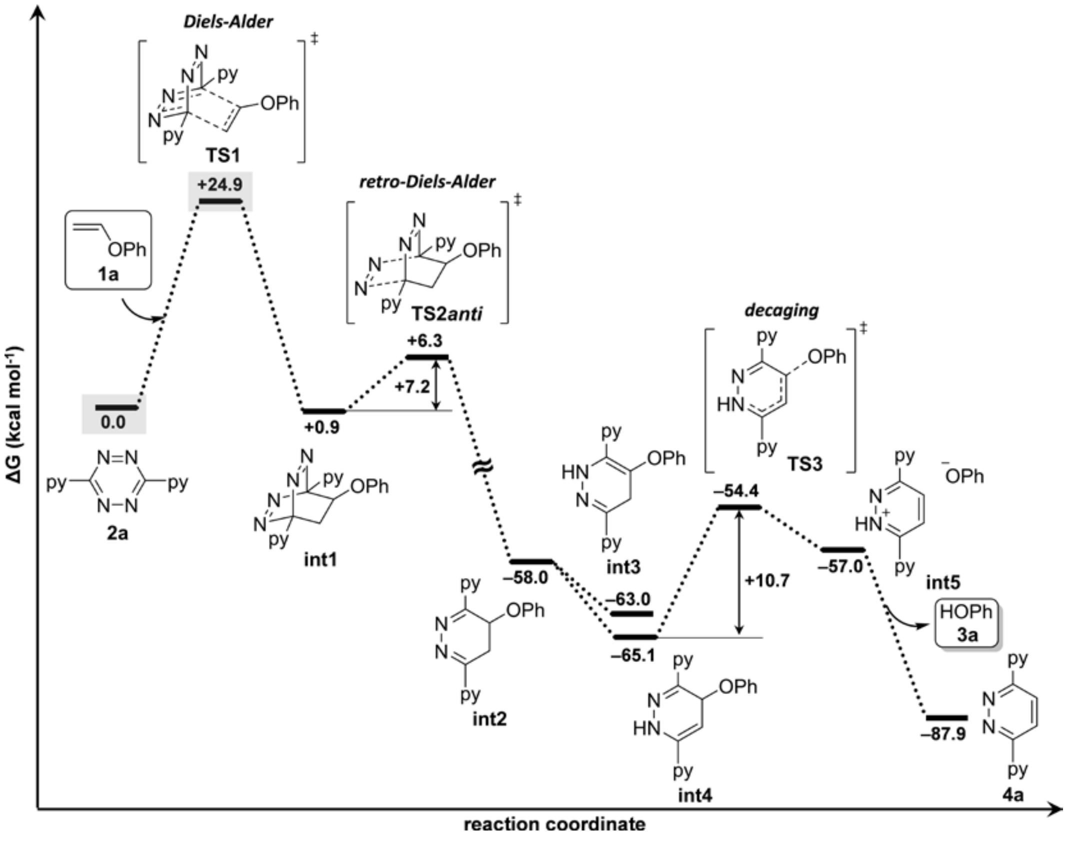

Figure S5: Guide to compound numbering of calculated structures (only the lowest energy conformers are shown)

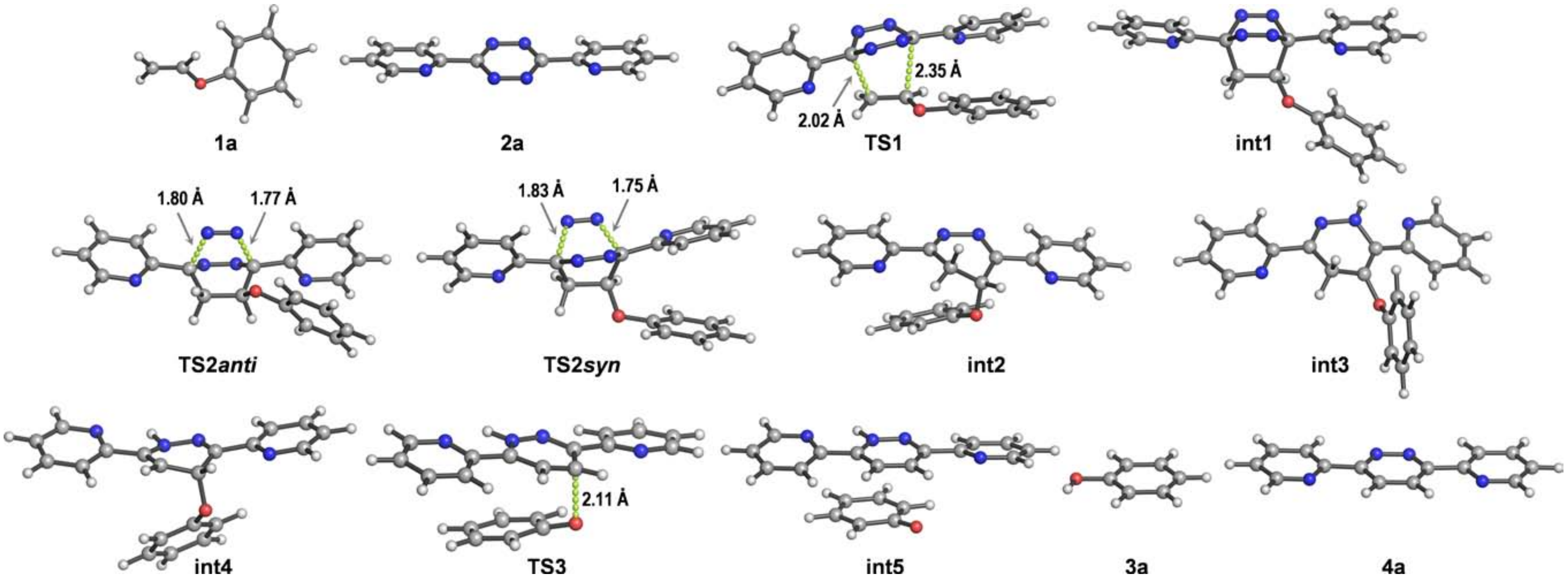

Table S2. Energies, entropies, and lowest frequencies of all the calculated structures.[a]

Structure	E_{elec} (Hartree)	E_{elec} + ZPE (Hartree)	H (Hartree)	S (cal mol^{-1} K^{-1})	G (Hartree)	Lowest freq. (cm^{-1})	# of imag freq.
**1a_a**	–384.711414	–384.572855	–384.564217	87.2		63.1	0
**1a_b**	–384.710651	–384.572122	–384.563476	87.9		35.8	0
**N2**	–109.492420	–109.486625	–109.483321	45.8		2543.5	0
**2a_a**	–790.249311	–790.057937	–790.043873	119.9		18.6	0
**2a_b**	–790.249460	–790.057978	–790.043955	119.1		25.1	0
**TS1_a**	–1174.951657	–1174.619213	–1174.597818	150.6		–426.4	1
**TS1_b**	–1174.951398	–1174.619003	–1174.597614	150.2		–427.1	1
**TS1_c**	–1174.947912	–1174.615650	–1174.594175	151.8		–445.9	1
**TS1_d**	–1174.947981	–1174.615597	–1174.594182	150.8		–443.3	1
**int1_a**	–1174.994082	–1174.658059	–1174.637098	149.9		25.7	0
**int1_b**	–1174.995935	–1174.660008	–1174.638943	153.7		8.2	0
**int1_c**	–1174.996010	–1174.660097	–1174.639104	151.1		17.8	0
**int1_d**	–1174.993982	–1174.658005	–1174.637009	151.3		16.1	0
**TS2*anti*_a**	–1174.982516	–1174.649189	–1174.628047	150.6		–580.2	1
**TS2*anti*_b**	–1174.979988	–1174.646420	–1174.625363	148.6		–584.7	1
**TS2*anti*_c**	–1174.977782	–1174.644482	–1174.623147	152.7		–577.3	1
**TS2*anti*_d**	–1174.975103	–1174.641727	–1174.620425	151.7		–582.1	1
**TS2*syn*_a**	–1174.980439	–1174.647052	–1174.625734	152.6		–568.1	1
**TS2*syn*_b**	–1174.980207	–1174.646840	–1174.625578	151.3		–568.8	1
**int2_a**	–1065.569642	–1065.244633	–1065.224397	149.2		22.9	0
**int2_b**	–1065.569318	–1065.244019	–1065.223675	151.7		12.5	0
**int2_c**	–1065.565378	–1065.240239	–1065.220088	147.6		22.2	0
**int2_d**	–1065.564751	–1065.239515	–1065.219186	151.1		12.3	0

**int2_e**	–1065.564159	–1065.239249	–1065.218814	153.7		8.8	0
**int2_f**	–1065.564094	–1065.238746	–1065.218402	151.2		15.5	0
**int2_g**	–1065.564009	–1065.238836	–1065.218621	148.5		27.9	0
**int2_h**	–1065.562183	–1065.236986	–1065.216717	149.6		21.0	0
**int2_i**	–1065.561234	–1065.235944	–1065.215740	148.8		20.5	0
**int2_j**	–1065.560526	–1065.235529	–1065.215158	151.3		21.1	0
**int2_k**	–1065.559069	–1065.234144	–1065.213772	150.2		20.2	0
**int2_l**	–1065.559357	–1065.233980	–1065.213679	150.0		15.2	0
**int2_m**	–1065.559592	–1065.234337	–1065.214216	146.6		24.9	0
**int2_n**	–1065.557079	–1065.231697	–1065.211482	148.6		20.9	0
**int2_o**	–1065.556087	–1065.230720	–1065.210529	148.1		24.6	0
**int2_p**	–1065.555220	–1065.230252	–1065.209868	150.9		22.2	0
**int3_a**	–1065.578106	–1065.252200	–1065.231829	150.3		22.9	0
**int3_b**	–1065.577174	–1065.251453	–1065.231032	152.4		14.1	0
**int3_c**	–1065.573848	–1065.247832	–1065.227510	149.7		23.3	0
**int3_d**	–1065.571460	–1065.245989	–1065.225491	151.3		15.7	0
**int3_e**	–1065.570209	–1065.244852	–1065.224265	152.9		13.8	0
**int3_f**	–1065.567203	–1065.241513	–1065.221109	150.0		18.9	0
**int3_g**	–1065.567203	–1065.241513	–1065.221109	150.0		18.9	0
**int3_h**	–1065.565966	–1065.240374	–1065.219868	151.8		14.4	0
**int4_a**	–1065.581927	–1065.255780	–1065.235570	149.1		23.9	0
**int4_b**	–1065.581527	–1065.255178	–1065.234883	150.7		13.6	0
**int4_c**	–1065.579109	–1065.252838	–1065.232719	147.3		25.4	0
**int4_d**	–1065.578099	–1065.251696	–1065.231435	150.2		14.0	0
**int4_e**	–1065.577218	–1065.251252	–1065.230955	150.6		16.0	0
**int4_f**	–1065.576430	–1065.250189	–1065.229830	150.8		14.2	0
**int4_g**	–1065.574399	–1065.248276	–1065.228093	147.8		24.3	0

**int4_h**	–1065.573062	–1065.246781	–1065.226465	150.0		14.1	0
**TS3_a**	–1065.562872	–1065.238869	–1065.218779	146.8		–252.8	1
**TS3_b**	–1065.560238	–1065.236022	–1065.216025	145.5		–249.5	1
**TS3_c**	–1065.557922	–1065.233882	–1065.213824	145.7		–246.7	1
**TS3_d**	–1065.555286	–1065.231203	–1065.211154	145.7		–236.6	1
**int5_a**	–1065.566010	–1065.241947	–1065.221088	149.4		33.8	0
**int5_b**	–1065.562909	–1065.238887	–1065.218018	149.7		33.2	0
**int5_c**	–1065.560363	–1065.236616	–1065.215616	150.6		31.2	0
**int5_d**	–1065.557156	–1065.233063	–1065.212216	149.0		32.7	0
**3a**	–307.361012	–307.255693	–307.249223	74.6		228.4	0
**4a_a**	–758.226686	–758.010469	–757.996258	121.7		7.5	0
**4a_b**	–758.222161	–758.005761	–757.991651	118.4		22.8	0
**4a_c**	–758.217480	–758.001062	–757.987003	117.0		37.3	0

[a]Energy values calculated at the PCM(H_2O)/M06-2X/6-31+G(d,p) level. 1 Hartree = 627.51 kcal mol^{-1}. Thermal corrections at 298.15 K.

Cartesian coordinates of all structures calculated with PCM(H_2O)/M06-2X/6-31+G(d,p)

Structure **N2**

```
 N     0.000000   0.000000   0.549080
 N     0.000000   0.000000  -0.549080
```

Structure **1a_a**

```
 C    -2.669131   0.366987  -0.021213
 C    -2.265203  -0.969945  -0.060510
 C    -0.913900  -1.299908  -0.023756
 C     0.037343  -0.282145   0.057962
 C    -0.348902   1.056747   0.113380
 C    -1.708286   1.372127   0.066193
 H    -3.723402   0.620313  -0.054122
 H    -3.005456  -1.761020  -0.125916
 H    -0.580464  -2.331820  -0.060082
 H     0.393666   1.841744   0.209872
 H    -2.011111   2.413566   0.108642
 O     1.353152  -0.676056   0.124857
 C     2.311368   0.183979  -0.347334
 H     1.986143   0.834465  -1.156400
 C     3.553033   0.155768   0.124939
 H     3.831477  -0.506747   0.937183
 H     4.306000   0.796286  -0.316004
```

Structure **1a_b**

```
 C    -2.456739  -0.000718   0.370485
 C    -1.794488  -1.208809   0.147251
 C    -0.471807  -1.213639  -0.295084
 C     0.171783   0.000710  -0.510473
 C    -0.472568   1.214342  -0.293453
 C    -1.795265   1.208084   0.148876
 H    -3.486252  -0.001329   0.713119
 H    -2.307364  -2.150417   0.314887
 H     0.063515  -2.140663  -0.474613
 H     0.062054   2.142016  -0.471685
 H    -2.308651   2.149191   0.317751
 O     1.471446   0.001513  -0.994804
 C     2.482217   0.000040  -0.070367
 H     3.442142   0.000331  -0.575233
 C     2.355951  -0.001278   1.255960
 H     1.393989  -0.001446   1.755423
 H     3.254492  -0.002174   1.859616
```

Structure **2a_a**

```
 N     0.652897  -1.177485   0.289514
 C     1.284941  -0.021880   0.037238
 C    -1.284941  -0.021878   0.037248
 N    -0.652896  -1.177485   0.289516
 N     0.651781   1.141148  -0.172820
 N    -0.651779   1.141149  -0.172816
 C     2.771898  -0.007279   0.005025
 C     3.458010   1.207131   0.079771
 N     3.382375  -1.193855  -0.098260
 C     4.849288   1.180768   0.049438
 H     2.911815   2.138611   0.163793
 C     4.714831  -1.202160  -0.129967
 C     5.494537  -0.046163  -0.057768
 H     5.415949   2.103765   0.110429
 H     5.185623  -2.177375  -0.219458
 H     6.575936  -0.116166  -0.086261
 C    -2.771898  -0.007278   0.005028
 C    -3.458010   1.207136   0.079713
 N    -3.382376  -1.193859  -0.098201
 C    -4.849288   1.180772   0.049379
 H    -2.911816   2.138620   0.163690
 C    -4.714832  -1.202165  -0.129909
 C    -5.494537  -0.046164  -0.057768
 H    -5.415949   2.103772   0.110324
 H    -5.185623  -2.177384  -0.219352
 H    -6.575937  -0.116168  -0.086258
```

Structure **2a_b**

```
 N    -0.643557  -1.191805  -0.057297
 C    -1.284873  -0.014518  -0.031342
 C     1.284886   0.014452  -0.031359
 N     0.661045  -1.172382  -0.048178
 N    -0.661018   1.172319  -0.048573
 N     0.643571   1.191743  -0.057702
 C    -2.771677  -0.006789  -0.002958
 C    -3.468577   1.160160  -0.324894
 N    -3.371297  -1.153073   0.340377
 C    -4.859667   1.127271  -0.290466
 H    -2.930332   2.059538  -0.597910
 C    -4.703699  -1.167341   0.372594
 C    -5.493780  -0.057927   0.065233
 H    -5.434713   2.012807  -0.539225
 H    -5.165758  -2.108079   0.659304
 H    -6.574534  -0.130980   0.107061
 C     2.771679   0.006770  -0.002950
 C     3.468628  -1.159939  -0.325644
 N     3.371226   1.152863   0.341142
 C     4.859715  -1.127005  -0.291192
 H     2.930410  -2.059154  -0.599257
 C     4.703631   1.167167   0.373389
 C     5.493762   0.057993   0.065300
 H     5.434811  -2.012342  -0.540542
 H     5.165646   2.107731   0.660739
 H     6.574512   0.131070   0.107187
```

Structure **TS1_a**

```
 C     1.434059  -0.371361   1.447581
 C     0.056706  -0.434724   1.322398
 H     1.843553   0.354492   2.140322
 H    -0.632099   0.241135   1.822475
 N     0.234204   0.208901  -1.332032
 C    -0.210626   1.119652  -0.418100
 C     2.186864   0.469046  -0.231771
 N     1.468129  -0.124102  -1.263325
 N     0.654870   2.105946  -0.016639
 N     1.892967   1.800459   0.022592
 O    -0.433311  -1.559026   0.768598
 C    -1.789498  -1.679645   0.495540
 C    -2.771574  -1.226916   1.371894
 C    -2.109576  -2.325094  -0.694064
 C    -4.109144  -1.402952   1.019788
 H    -2.510097  -0.750277   2.311048
 C    -3.450496  -2.502155  -1.026024
 H    -1.309133  -2.660047  -1.345226
 C    -4.452787  -2.035429  -0.174993
```

H	-4.884024	-1.044164	1.689621
H	-3.710254	-2.997657	-1.955887
H	-5.496898	-2.165935	-0.439372
H	1.983744	-1.298017	1.312006
C	-1.663339	1.419207	-0.343778
C	-2.538364	1.026316	-1.358964
N	-2.058105	2.061485	0.761870
C	-3.888874	1.321763	-1.212602
H	-2.160434	0.497837	-2.226385
C	-3.357913	2.342796	0.885329
C	-4.311555	1.996474	-0.069869
H	-4.599922	1.027047	-1.977643
H	-3.651239	2.861394	1.794297
H	-5.355636	2.242309	0.088060
C	3.631326	0.082259	-0.140743
C	4.619728	1.044305	0.066753
N	3.890834	-1.223209	-0.267772
C	5.945348	0.621425	0.132841
H	4.351157	2.088826	0.164096
C	5.165109	-1.613528	-0.206962
C	6.228827	-0.732488	-0.009473
H	6.741543	1.341527	0.290244
H	5.345271	-2.679878	-0.313422
H	7.245333	-1.106433	0.035435

Structure **TS1_b**

C	1.445781	-0.303470	1.431815
C	0.066505	-0.363234	1.323503
H	1.882844	0.461323	2.062064
H	-0.611425	0.353571	1.779654
N	0.227541	0.114274	-1.369476
C	-0.211090	1.076284	-0.507382
C	2.187379	0.436586	-0.298244
N	1.464118	-0.208743	-1.286608
N	0.657840	2.084500	-0.170234
N	1.896060	1.786374	-0.116127
O	-0.437626	-1.521757	0.859519
C	-1.797299	-1.651673	0.609397
C	-2.766528	-1.131575	1.462292
C	-2.134711	-2.377498	-0.527941
C	-4.109007	-1.322975	1.138300
H	-2.491099	-0.590837	2.361929
C	-3.480443	-2.568636	-0.831667
H	-1.343907	-2.763580	-1.162390
C	-4.470083	-2.036327	-0.004665
H	-4.874026	-0.912579	1.789573
H	-3.753756	-3.126967	-1.721166
H	-5.517904	-2.178448	-0.247489
H	1.979557	-1.246795	1.365472
C	-1.663027	1.381625	-0.440462
C	-2.547178	0.926412	-1.420975
N	-2.047468	2.093389	0.625576
C	-3.896042	1.232985	-1.282072
H	-2.177431	0.343158	-2.256272
C	-3.345745	2.384412	0.742094
C	-4.307939	1.979952	-0.181158
H	-4.614100	0.891450	-2.020635
H	-3.630605	2.959663	1.619071
H	-5.350345	2.237087	-0.030310
C	3.632341	0.063824	-0.176235
C	4.223032	-0.828703	-1.071176
N	4.289433	0.629523	0.841556
C	5.570796	-1.134873	-0.900655
H	3.639445	-1.258275	-1.875948
C	5.581083	0.328841	0.990743
C	6.268357	-0.544906	0.148246
H	6.065814	-1.821398	-1.579591
H	6.089980	0.803315	1.825512
H	7.318217	-0.754159	0.319019

Structure **TS1_c**

C	1.433756	-0.520230	1.296092
C	0.051463	-0.507552	1.194043
H	1.896537	0.119064	2.038070
H	-0.580887	0.176092	1.750944
N	0.313715	0.293516	-1.447664
C	-0.151844	1.154074	-0.493375
C	2.232077	0.447546	-0.263389
N	1.541662	-0.052036	-1.356765
N	0.715509	2.088018	0.009630
N	1.948469	1.766594	0.080396
O	-0.519889	-1.568539	0.603212
C	-1.899885	-1.678166	0.485879
C	-2.786575	-1.150504	1.419499
C	-2.344697	-2.403534	-0.614497
C	-4.154846	-1.332361	1.214723
H	-2.438366	-0.616634	2.296813
C	-3.712323	-2.585082	-0.797946
H	-1.617284	-2.798584	-1.315714
C	-4.622475	-2.044821	0.111697
H	-4.854562	-0.915279	1.931898
H	-4.066027	-3.144055	-1.658220
H	-5.688610	-2.179862	-0.036748
H	1.923785	-1.468675	1.096391
C	-1.601768	1.476406	-0.431086
C	-2.068876	2.325025	0.577025
N	-2.405206	0.894446	-1.328808
C	-3.435918	2.570303	0.657197
H	-1.372454	2.771694	1.278587
C	-3.713047	1.135966	-1.236651
C	-4.280715	1.960593	-0.264384
H	-3.832218	3.219973	1.430667
H	-4.342553	0.644154	-1.974126
H	-5.354075	2.110936	-0.235487
C	3.670498	0.045109	-0.144835
C	4.288367	-0.716327	-1.137038
N	4.292996	0.451807	0.966241
C	5.627065	-1.059017	-0.964038
H	3.732374	-1.019642	-2.015433
C	5.576406	0.118458	1.117028
C	6.288932	-0.633527	0.182963
H	6.142597	-1.646916	-1.716299
H	6.056908	0.462699	2.028900
H	7.330387	-0.877223	0.359427

Structure **TS1_d**

C	1.429732	-0.498583	1.350262
C	0.048834	-0.512367	1.235613
H	1.857771	0.147886	2.107561
H	-0.604163	0.148400	1.796382
N	0.297635	0.300468	-1.395350
C	-0.166901	1.163517	-0.442328
C	2.219743	0.462916	-0.217528
N	1.524497	-0.047924	-1.310835
N	0.699624	2.100180	0.055812
N	1.933561	1.777207	0.118051

O	-0.493653	-1.573509	0.619041
C	-1.868518	-1.687077	0.452380
C	-2.787286	-1.193517	1.373737
C	-2.273503	-2.378523	-0.684183
C	-4.147117	-1.374428	1.119581
H	-2.469167	-0.686468	2.278109
C	-3.633778	-2.560258	-0.917050
H	-1.521715	-2.747104	-1.374003
C	-4.575137	-2.053339	-0.020349
H	-4.871882	-0.983976	1.826793
H	-3.956911	-3.092766	-1.805685
H	-5.635257	-2.187974	-0.207369
H	1.948454	-1.429418	1.140642
C	-1.616572	1.487018	-0.377815
C	-2.085589	2.302706	0.656271
N	-2.416888	0.943326	-1.301964
C	-3.451548	2.553982	0.736080
H	-1.391301	2.719711	1.378060
C	-3.723699	1.190724	-1.210312
C	-4.293330	1.983286	-0.212758
H	-3.849227	3.178129	1.529615
H	-4.351008	0.731215	-1.970133
H	-5.365861	2.139842	-0.186403
C	3.660191	0.058134	-0.120902
C	4.650536	0.994505	0.175248
N	3.913049	-1.237595	-0.331789
C	5.971111	0.555895	0.242123
H	4.387653	2.032097	0.339022
C	5.182530	-1.642926	-0.269909
C	6.247877	-0.787281	0.012225
H	6.768747	1.256019	0.467605
H	5.357017	-2.700911	-0.446289
H	7.260284	-1.172528	0.053971

Structure **int1_a**

C	-1.382969	-0.977254	-0.515804
C	0.068905	-0.513992	-0.539284
H	-1.754337	-1.244917	-1.504392
H	0.445991	-0.445556	-1.561546
N	-0.553628	0.851800	1.399694
C	0.088806	0.943653	0.072520
C	-2.250315	0.186779	0.024510
N	-1.732579	0.484816	1.370077
N	-0.787210	1.732855	-0.814231
N	-1.963305	1.359435	-0.843575
O	0.840604	-1.392414	0.241057
C	2.169729	-1.616553	-0.031960
C	2.876380	-1.077307	-1.106751
C	2.806345	-2.471718	0.872005
C	4.227338	-1.399529	-1.261498
H	2.418252	-0.396716	-1.813615
C	4.150790	-2.782288	0.704336
H	2.231898	-2.875461	1.699370
C	4.872902	-2.246363	-0.365060
H	4.773417	-0.969656	-2.095555
H	4.636163	-3.444965	1.413959
H	5.923123	-2.484818	-0.494116
H	-1.489826	-1.830655	0.156783
C	1.474435	1.535517	0.161362
C	1.979727	2.319284	-0.876855
N	2.188434	1.187668	1.234806
C	3.302228	2.746426	-0.795815
H	1.355298	2.576306	-1.724425
C	3.455126	1.604958	1.299787
C	4.061030	2.378671	0.310910
H	3.731284	3.352586	-1.586920
H	4.013612	1.302622	2.182013
H	5.096722	2.682457	0.413357
C	-3.729411	-0.112484	0.028307
C	-4.503845	-0.023824	1.183125
N	-4.224146	-0.458287	-1.164911
C	-5.864265	-0.311208	1.085343
H	-4.052164	0.259253	2.125592
C	-5.528823	-0.727879	-1.245003
C	-6.391178	-0.669869	-0.150221
H	-6.499340	-0.253383	1.963230
H	-5.902264	-1.004551	-2.227283
H	-7.443224	-0.901362	-0.271655

Structure **int1_b**

C	-1.425953	-0.843924	-0.798268
C	0.042294	-0.448866	-0.731540
H	-1.819507	-0.843434	-1.814039
H	0.463064	-0.240068	-1.716858
N	-0.468973	0.561802	1.414793
C	0.115315	0.886174	0.104995
C	-2.238685	0.191352	0.023398
N	-1.654475	0.212822	1.373447
N	-0.780470	1.849901	-0.577778
N	-1.964953	1.509029	-0.611264
O	0.735943	-1.478977	-0.060066
C	2.102390	-1.620272	-0.169067
C	2.871791	-1.122227	-1.219906
C	2.696697	-2.361563	0.854789
C	4.247728	-1.360524	-1.224074
H	2.433454	-0.538793	-2.020494
C	4.067539	-2.596172	0.833122
H	2.071180	-2.735730	1.658793
C	4.853727	-2.092302	-0.205455
H	4.845145	-0.961636	-2.038191
H	4.522766	-3.169380	1.634646
H	5.924071	-2.267925	-0.219030
H	-1.565093	-1.831337	-0.354558
C	1.515339	1.436892	0.208774
C	2.262288	1.336535	1.380471
N	1.991896	1.949593	-0.929222
C	3.580147	1.788198	1.358310
H	1.826140	0.903326	2.272463
C	3.252440	2.387388	-0.934231
C	4.089011	2.324402	0.179960
H	4.197750	1.717871	2.247778
H	3.614337	2.798636	-1.872896
H	5.109676	2.684431	0.116530
C	-3.722240	-0.084452	0.041099
C	-4.442864	-0.206625	1.227407
N	-4.276970	-0.191762	-1.170934
C	-5.811916	-0.453925	1.142007
H	-3.944466	-0.112598	2.184027
C	-5.589246	-0.425835	-1.238237
C	-6.400693	-0.566064	-0.112512
H	-6.405976	-0.555520	2.044312
H	-6.011509	-0.506814	-2.236246
H	-7.461627	-0.758056	-0.225229

Structure **int1_c**

C	1.442832	0.675387	-1.018402

C	-0.036628	0.348789	-0.879181
H	1.812577	0.504676	-2.030152
H	-0.490664	0.041247	-1.823407
N	0.448851	-0.386658	1.374346
C	-0.138336	-0.873519	0.114164
C	2.231657	-0.252703	-0.057060
N	1.637627	-0.065608	1.295006
N	0.739204	-1.936075	-0.426221
N	1.933244	-1.626031	-0.492363
O	-0.668813	1.485153	-0.326405
C	-2.042186	1.610819	-0.326994
C	-2.879941	1.057190	-1.294309
C	-2.566368	2.392766	0.703512
C	-4.255493	1.274692	-1.203377
H	-2.488192	0.446481	-2.099337
C	-3.939168	2.609717	0.775543
H	-1.887327	2.810066	1.440079
C	-4.793090	2.046054	-0.174439
H	-4.907431	0.831211	-1.949731
H	-4.342063	3.214521	1.581866
H	-5.864196	2.206641	-0.113186
H	1.631969	1.709968	-0.731908
C	-1.545049	-1.391967	0.280452
C	-2.275432	-1.197870	1.450616
N	-2.038318	-1.990852	-0.807242
C	-3.596200	-1.640213	1.479252
H	-1.825797	-0.700082	2.301167
C	-3.301716	-2.418170	-0.763407
C	-4.123974	-2.261109	0.351872
H	-4.201728	-1.497215	2.368351
H	-3.677423	-2.899900	-1.662274
H	-5.147969	-2.616524	0.328745
C	3.713137	0.031219	-0.019614
C	4.665006	-0.963591	-0.234042
N	4.026435	1.302957	0.249774
C	6.011658	-0.610831	-0.165017
H	4.357662	-1.979205	-0.449614
C	5.319675	1.626504	0.316200
C	6.350091	0.708179	0.115557
H	6.781275	-1.358429	-0.326779
H	5.543215	2.666668	0.537656
H	7.384445	1.026470	0.178701

Structure **int1_d**

C	-1.380456	-0.876734	-0.709633
C	0.074928	-0.428993	-0.642697
H	-1.718570	-1.021103	-1.736257
H	0.479934	-0.253141	-1.640993
N	-0.573256	0.705238	1.427337
C	0.089944	0.951953	0.126385
C	-2.252446	0.223993	-0.055860
N	-1.749651	0.348904	1.341084
N	-0.768661	1.843332	-0.670222
N	-1.947977	1.480393	-0.757055
O	0.819516	-1.395047	0.056713
C	2.154183	-1.600625	-0.204443
C	2.891380	-0.958285	-1.199128
C	2.762541	-2.550145	0.621402
C	4.244171	-1.273447	-1.352202
H	2.455632	-0.204842	-1.843415
C	4.109288	-2.852190	0.456525
H	2.164732	-3.032929	1.387595
C	4.861817	-2.213670	-0.532534
H	4.813967	-0.763585	-2.122954
H	4.572624	-3.588584	1.105618
H	5.913840	-2.445568	-0.658866
H	-1.521163	-1.801289	-0.148924
C	1.476446	1.522310	0.302520
C	2.004897	2.401697	-0.643546
N	2.168889	1.064885	1.348624
C	3.327671	2.809820	-0.497151
H	1.397204	2.744867	-1.472670
C	3.436375	1.464941	1.477002
C	4.064250	2.328915	0.580849
H	3.774265	3.488129	-1.216748
H	3.977065	1.072696	2.334520
H	5.099489	2.613674	0.731227
C	-3.728166	-0.089619	-0.052539
C	-4.679280	0.808603	-0.532531
N	-4.038589	-1.284142	0.461976
C	-6.021822	0.438709	-0.473030
H	-4.374616	1.764227	-0.940337
C	-5.328041	-1.624471	0.515819
C	-6.357252	-0.800349	0.061002
H	-6.790750	1.111862	-0.837693
H	-5.549297	-2.600305	0.939774
H	-7.388335	-1.128499	0.126953

Structure **TS2*anti*_a**

C	-1.471712	-0.554996	-1.212916
C	0.029625	-0.351301	-1.072182
H	-1.880255	-0.117191	-2.123759
H	0.505489	-0.001841	-1.991949
N	-0.278667	0.311392	1.258978
C	0.294961	0.670146	0.062096
C	-2.167575	0.044307	0.004743
N	-1.522026	-0.022690	1.224550
N	-0.725547	2.006157	-0.477874
N	-1.861558	1.757155	-0.461439
O	0.546392	-1.622415	-0.673889
C	1.888067	-1.718476	-0.357809
C	2.887904	-1.297098	-1.233974
C	2.206451	-2.287163	0.872683
C	4.221053	-1.415866	-0.846998
H	2.628904	-0.857751	-2.192160
C	3.544578	-2.416951	1.242251
H	1.401929	-2.603029	1.529240
C	4.554520	-1.972413	0.389475
H	5.002335	-1.072375	-1.518009
H	3.795088	-2.854526	2.203525
H	5.595363	-2.060364	0.683365
H	-1.678534	-1.627171	-1.232133
C	1.676106	1.249211	0.138264
C	2.439153	1.179803	1.303145
N	2.112916	1.801012	-0.998320
C	3.732751	1.691667	1.270284
H	2.030486	0.714637	2.192191
C	3.350689	2.300078	-1.011800
C	4.202477	2.263582	0.091154
H	4.364716	1.639594	2.151027
H	3.679410	2.743676	-1.947969
H	5.204482	2.671513	0.020347
C	-3.651563	-0.105424	0.087956
C	-4.342912	0.330126	1.221096
N	-4.256440	-0.628470	-0.983767
C	-5.726870	0.197210	1.242520

H	-3.799408	0.757638	2.055949
C	-5.587012	-0.749070	-0.950796
C	-6.365951	-0.355599	0.135317
H	-6.296875	0.522694	2.106499
H	-6.050723	-1.180717	-1.833664
H	-7.442726	-0.477985	0.106659

Structure **TS2*anti*_b**

C	-1.453975	-0.637207	-1.209375
C	0.045997	-0.405675	-1.073286
H	-1.856333	-0.248442	-2.147240
H	0.516196	-0.076240	-2.003214
N	-0.291576	0.326870	1.230361
C	0.294391	0.644969	0.032364
C	-2.169976	0.007351	-0.027386
N	-1.535957	-0.012516	1.199916
N	-0.746552	1.970094	-0.571719
N	-1.876376	1.704681	-0.555368
O	0.577409	-1.660916	-0.642693
C	1.916543	-1.728942	-0.307319
C	2.920735	-1.326942	-1.187308
C	2.227231	-2.248196	0.946675
C	4.250725	-1.414274	-0.781434
H	2.666503	-0.925950	-2.163482
C	3.562551	-2.347900	1.335308
H	1.419076	-2.549970	1.605398
C	4.576574	-1.921825	0.477959
H	5.035495	-1.085335	-1.455637
H	3.807419	-2.747161	2.314534
H	5.614976	-1.986071	0.786251
H	-1.622320	-1.716443	-1.178536
C	1.665256	1.246480	0.099752
C	2.415804	1.234085	1.274887
N	2.106941	1.761650	-1.052275
C	3.701515	1.764954	1.236811
H	2.004025	0.796899	2.176641
C	3.336591	2.280097	-1.070866
C	4.175914	2.299443	0.042048
H	4.323979	1.756304	2.125781
H	3.669310	2.693070	-2.019573
H	5.172053	2.720738	-0.033087
C	-3.657063	-0.120649	0.059728
C	-4.371929	-0.981198	-0.773059
N	-4.243666	0.665397	0.972120
C	-5.758136	-1.038318	-0.638160
H	-3.868284	-1.601352	-1.505397
C	-5.569742	0.601282	1.091349
C	-6.373191	-0.234643	0.313477
H	-6.341840	-1.701524	-1.267794
H	-6.016020	1.251692	1.839041
H	-7.448093	-0.243471	0.453630

Structure **TS2*anti*_c**

C	-1.389494	-0.965933	-0.649130
C	0.069531	-0.517441	-0.614385
H	-1.764233	-1.091156	-1.664502
H	0.480297	-0.429254	-1.622831
N	-0.540990	0.941366	1.290249
C	0.155950	0.849150	0.109440
C	-2.258293	0.029355	0.102858
N	-1.766771	0.540488	1.279575
N	-0.842146	1.769037	-1.015076
N	-1.955821	1.439764	-1.026801
O	0.791552	-1.468037	0.150538
C	2.146172	-1.643406	-0.012174
C	2.897306	-1.159365	-1.083255
C	2.757472	-2.400732	0.991036
C	4.267018	-1.430532	-1.130686
H	2.454126	-0.565716	-1.873060
C	4.120582	-2.666742	0.927060
H	2.148780	-2.764445	1.812490
C	4.887335	-2.179140	-0.134084
H	4.847273	-1.042956	-1.962474
H	4.585839	-3.253234	1.713113
H	5.952193	-2.380160	-0.181152
H	-1.472970	-1.928919	-0.138625
C	1.522213	1.478082	0.134273
C	2.029459	2.140868	-0.984697
N	2.228183	1.281177	1.252096
C	3.338897	2.610166	-0.938319
H	1.419676	2.279431	-1.870160
C	3.484798	1.731937	1.280342
C	4.088544	2.396761	0.213796
H	3.765315	3.127944	-1.791153
H	4.036844	1.549687	2.198758
H	5.115443	2.735383	0.292379
C	-3.734175	-0.194418	0.091176
C	-4.576473	0.627313	0.844327
N	-4.181406	-1.178387	-0.696067
C	-5.946914	0.400437	0.784423
H	-4.156647	1.417602	1.456235
C	-5.500795	-1.385544	-0.744640
C	-6.422801	-0.628523	-0.024838
H	-6.631709	1.017893	1.356331
H	-5.835174	-2.194045	-1.389017
H	-7.482799	-0.841632	-0.103604

Structure **TS2*anti*_d**

C	1.375431	0.987547	-0.675203
C	-0.080520	0.524040	-0.634506
H	1.736250	1.103060	-1.699021
H	-0.486211	0.406217	-1.641965
N	0.549899	-0.880788	1.300518
C	-0.155798	-0.818464	0.127883
C	2.258614	0.010966	0.085606
N	1.776794	-0.476560	1.276085
N	0.862035	-1.766817	-0.996113
N	1.968282	-1.420803	-1.013976
O	-0.809788	1.492450	0.101107
C	-2.169157	1.640036	-0.053902
C	-2.916814	1.129181	-1.114691
C	-2.787782	2.398902	0.943310
C	-4.291601	1.374049	-1.157036
H	-2.466429	0.534611	-1.899824
C	-4.155888	2.639510	0.883930
H	-2.181061	2.783472	1.756669
C	-4.919496	2.124219	-0.166246
H	-4.869621	0.965498	-1.980264
H	-4.627378	3.227416	1.665179
H	-5.988155	2.304943	-0.209349
H	1.431329	1.958292	-0.174590
C	-1.513094	-1.463987	0.167487
C	-2.015261	-2.148993	-0.940416
N	-2.217135	-1.263250	1.285993
C	-3.316718	-2.638414	-0.881771
H	-1.407268	-2.289172	-1.826870

C	-3.466329	-1.733461	1.325928
C	-4.064228	-2.422023	0.271194
H	-3.738767	-3.173999	-1.725730
H	-4.017198	-1.547595	2.244326
H	-5.085188	-2.775916	0.359268
C	3.737678	0.220774	0.064840
C	4.305821	1.395912	-0.426524
N	4.464763	-0.800892	0.535232
C	5.694178	1.518801	-0.413600
H	3.688014	2.203747	-0.801653
C	5.791487	-0.671534	0.540537
C	6.455809	0.466841	0.080003
H	6.166781	2.422647	-0.783257
H	6.354353	-1.517799	0.925526
H	7.538390	0.515339	0.107589

Structure **TS2*syn*_a**

C	-1.368598	-0.917747	-0.706872
C	0.084535	-0.450958	-0.646847
H	-1.650934	-1.197421	-1.723700
H	0.512684	-0.398168	-1.650589
N	-0.564425	0.646144	1.495024
C	0.127275	0.986202	-0.079625
C	-2.292959	0.210003	-0.267880
N	-1.672387	0.302920	1.456011
N	-0.774894	1.868998	-0.623743
N	-2.002797	1.477237	-0.703521
O	0.812520	-1.344127	0.172256
C	2.144161	-1.603991	-0.052354
C	2.905551	-1.074253	-1.094666
C	2.725205	-2.478168	0.871287
C	4.252916	-1.429803	-1.199801
H	2.491874	-0.374837	-1.810881
C	4.067449	-2.819949	0.753726
H	2.109767	-2.872832	1.673240
C	4.842675	-2.296873	-0.284399
H	4.841282	-1.008674	-2.009235
H	4.509207	-3.497498	1.477632
H	5.890925	-2.560594	-0.374469
H	-1.512133	-1.789461	-0.066654
C	1.508954	1.545671	0.118308
C	2.226245	1.349583	1.298168
N	2.016517	2.156859	-0.957753
C	3.537545	1.814515	1.357047
H	1.776284	0.836372	2.139268
C	3.274433	2.599705	-0.886006
C	4.077149	2.451077	0.244389
H	4.127054	1.674683	2.257241
H	3.660325	3.090262	-1.775634
H	5.095105	2.824044	0.243366
C	-3.748923	-0.098364	-0.157472
C	-4.667196	0.918717	0.115918
N	-4.096728	-1.382136	-0.295940
C	-6.010962	0.581474	0.233140
H	-4.324639	1.940889	0.229563
C	-5.391328	-1.692713	-0.178824
C	-6.385123	-0.751656	0.081526
H	-6.752666	1.345028	0.443279
H	-5.644678	-2.742501	-0.299413
H	-7.420636	-1.061269	0.165815

Structure **TS2*syn*_b**

C	-1.358945	-0.970796	-0.617861
C	0.091857	-0.490990	-0.601057
H	-1.641711	-1.332369	-1.608201
H	0.488315	-0.459111	-1.619013
N	-0.581589	0.714958	1.492308
C	0.131263	0.968475	-0.079537
C	-2.287630	0.181068	-0.261756
N	-1.690959	0.373651	1.454473
N	-0.764640	1.814758	-0.694749
N	-1.989886	1.419143	-0.775082
O	0.843603	-1.371737	0.203305
C	2.167651	-1.623687	-0.060036
C	2.903171	-1.067232	-1.107264
C	2.773023	-2.520640	0.825049
C	4.247884	-1.417240	-1.255473
H	2.471559	-0.351203	-1.796074
C	4.112205	-2.857274	0.665443
H	2.178283	-2.936681	1.631771
C	4.861277	-2.306623	-0.377619
H	4.815372	-0.974778	-2.068386
H	4.572318	-3.552750	1.360425
H	5.907173	-2.566374	-0.500646
H	-1.492467	-1.790968	0.089585
C	1.495067	1.562927	0.125572
C	2.008346	2.450291	-0.822267
N	2.184667	1.143180	1.190287
C	3.313846	2.904963	-0.661142
H	1.398007	2.758807	-1.663300
C	3.433429	1.592787	1.335472
C	4.046801	2.465061	0.437096
H	3.750170	3.587279	-1.383258
H	3.971507	1.234887	2.209551
H	5.068455	2.788428	0.601597
C	-3.745181	-0.119193	-0.153472
C	-4.664677	0.912133	0.054729
N	-4.093825	-1.408090	-0.227570
C	-6.010462	0.583528	0.173266
H	-4.321456	1.938440	0.118733
C	-5.390352	-1.710217	-0.109328
C	-6.385373	-0.755313	0.088457
H	-6.753134	1.358196	0.333434
H	-5.644377	-2.764613	-0.176614
H	-7.422411	-1.058671	0.176663

Structure **int2_a**

C	0.903536	-0.738603	-1.206417
C	1.588969	-0.732594	0.146543
C	-1.090813	-1.144734	0.206661
C	-0.403037	-1.503661	-1.076161
H	1.562363	-1.235371	-1.917037
H	-1.059333	-1.334334	-1.930082
H	-0.170249	-2.577022	-1.053445
N	-0.439347	-0.846000	1.274587
N	0.956775	-0.868688	1.258340
O	0.762128	0.587813	-1.734823
C	-0.035885	1.451788	-1.005122
C	-1.385455	1.577199	-1.334165
C	0.521031	2.205356	0.027448
C	-2.194912	2.434415	-0.590294
H	-1.791826	0.992560	-2.153989
C	-0.295969	3.060798	0.765542
H	1.580653	2.111325	0.247177
C	-1.655064	3.170184	0.465429
H	-3.249046	2.522682	-0.835417

H	0.131355	3.644136	1.575003
H	-2.287648	3.834246	1.045407
C	3.067873	-0.579875	0.202916
C	3.708712	-0.248870	1.402393
N	3.728696	-0.756996	-0.949662
C	5.092317	-0.118798	1.402382
H	3.120595	-0.094905	2.299188
C	5.057218	-0.626452	-0.935930
C	5.786120	-0.312684	0.210136
H	5.619965	0.137166	2.315346
H	5.563531	-0.779318	-1.885268
H	6.864985	-0.218239	0.161312
C	-2.577975	-1.093664	0.272802
C	-3.234645	-0.797980	1.472732
N	-3.229389	-1.324834	-0.874205
C	-4.621964	-0.726374	1.469789
H	-2.656057	-0.628278	2.372569
C	-4.563033	-1.258147	-0.862390
C	-5.305840	-0.958197	0.277978
H	-5.161819	-0.493962	2.381940
H	-5.060942	-1.450645	-1.808996
H	-6.387804	-0.911556	0.227816

Structure **int2_b**

C	0.674115	0.063453	-0.571610
C	1.403650	-1.154340	-0.046780
C	-1.288343	-1.371573	-0.245441
C	-0.631668	-0.407443	-1.187603
H	1.312403	0.563360	-1.301900
H	-1.309322	0.420247	-1.400079
H	-0.422597	-0.913098	-2.140154
N	-0.612486	-2.152336	0.521479
N	0.778500	-2.182745	0.399749
O	0.463901	0.903286	0.560912
C	0.113556	2.211280	0.357423
C	0.018502	2.822290	-0.894753
C	-0.144858	2.945908	1.520327
C	-0.331228	4.173859	-0.968190
H	0.206122	2.274142	-1.810401
C	-0.491288	4.288407	1.429585
H	-0.064873	2.446320	2.480403
C	-0.587029	4.913080	0.182611
H	-0.403805	4.643086	-1.944385
H	-0.687679	4.849968	2.337567
H	-0.859075	5.960770	0.112673
C	2.890522	-1.172467	-0.051559
C	3.601889	-2.265547	0.456424
N	3.489504	-0.084386	-0.553661
C	4.990043	-2.223490	0.427722
H	3.064544	-3.114623	0.861245
C	4.824320	-0.053909	-0.568582
C	5.619664	-1.095330	-0.094573
H	5.572486	-3.055268	0.810161
H	5.278753	0.843220	-0.980186
H	6.700236	-1.018220	-0.134781
C	-2.772563	-1.427523	-0.146672
C	-3.407070	-2.472509	0.535189
N	-3.446234	-0.437178	-0.747247
C	-4.794596	-2.473927	0.604993
H	-2.812267	-3.256945	0.986958
C	-4.779459	-0.451997	-0.674524
C	-5.500621	-1.442527	-0.010440
H	-5.317303	-3.269428	1.125877
H	-5.295334	0.366218	-1.169732
H	-6.583708	-1.403696	0.015933

Structure **int2_c**

C	0.899002	-0.495125	-1.307483
C	1.593562	-0.731665	0.026432
C	-1.089192	-1.153661	0.012387
C	-0.399810	-1.284232	-1.311599
H	1.543646	-0.861531	-2.108113
H	-1.055500	-0.972931	-2.125066
H	-0.160019	-2.343763	-1.474344
N	-0.439227	-1.029267	1.114928
N	0.958071	-1.047319	1.097634
O	0.739426	0.898544	-1.605629
C	-0.054143	1.624041	-0.732274
C	-1.406980	1.795944	-1.022880
C	0.513123	2.195275	0.405625
C	-2.210100	2.513493	-0.137688
H	-1.820414	1.354745	-1.924778
C	-0.297954	2.911507	1.284944
H	1.575282	2.069842	0.595020
C	-1.660378	3.063856	1.020890
H	-3.266902	2.637788	-0.353691
H	0.136531	3.352487	2.176431
H	-2.288301	3.618950	1.710129
C	3.079298	-0.617691	0.090368
C	3.749341	0.175554	-0.847220
N	3.716676	-1.277488	1.068760
C	5.134389	0.292284	-0.758893
H	3.195908	0.705298	-1.615389
C	5.042540	-1.164038	1.134766
C	5.799912	-0.392329	0.250632
H	5.678578	0.908063	-1.467262
H	5.530730	-1.718470	1.932274
H	6.877225	-0.338145	0.358633
C	-2.576605	-1.133872	0.084002
C	-3.236418	-1.078825	1.316859
N	-3.225391	-1.152923	-1.087517
C	-4.624593	-1.025077	1.324349
H	-2.660167	-1.076522	2.233998
C	-4.559659	-1.106249	-1.066247
C	-5.305711	-1.036080	0.108827
H	-5.167082	-0.976498	2.262845
H	-5.055373	-1.122852	-2.033208
H	-6.388204	-0.994500	0.066134

Structure **int2_d**

C	0.680925	0.056158	-0.570632
C	1.427792	-1.144509	-0.020698
C	-1.264418	-1.389693	-0.206432
C	-0.619708	-0.444095	-1.174550
H	1.296358	0.545375	-1.331249
H	-1.305662	0.371383	-1.406572
H	-0.405287	-0.970940	-2.114201
N	-0.579823	-2.138492	0.583486
N	0.813277	-2.162278	0.459938
O	0.469529	0.927533	0.535738
C	0.076200	2.220047	0.298257
C	-0.027409	2.796999	-0.968785
C	-0.207586	2.973322	1.442536
C	-0.414918	4.136109	-1.076296
H	0.179689	2.233139	-1.870650
C	-0.591055	4.302942	1.317728
H	-0.118570	2.498729	2.414353

C	-0.697433	4.894370	0.055511
H	-0.495409	4.579672	-2.063695
H	-0.808078	4.880413	2.210830
H	-0.998753	5.931892	-0.040678
C	2.916721	-1.142116	-0.037613
C	3.599894	0.077694	-0.013426
N	3.544023	-2.326731	-0.053599
C	4.992135	0.065012	0.003479
H	3.056381	1.016709	0.013960
C	4.876726	-2.323297	-0.048737
C	5.648593	-1.159683	-0.018390
H	5.548907	0.995642	0.033217
H	5.357508	-3.297880	-0.072720
H	6.730785	-1.222910	-0.013681
C	-2.747948	-1.459837	-0.108589
C	-3.371426	-2.498072	0.593121
N	-3.431238	-0.488637	-0.729159
C	-4.758971	-2.512739	0.662236
H	-2.768557	-3.267341	1.060069
C	-4.764251	-0.516076	-0.656845
C	-5.475252	-1.501063	0.026352
H	-5.273714	-3.303449	1.198114
H	-5.288451	0.286654	-1.168358
H	-6.558711	-1.473214	0.051326

Structure **int2_e**

C	-0.254159	0.027279	0.097067
C	-0.519491	-1.464805	0.113565
C	2.078429	-0.730703	-0.103780
C	1.067481	0.250658	-0.622507
H	-0.219165	0.383495	1.136216
H	0.915870	0.073108	-1.696758
H	1.436431	1.269332	-0.506535
N	1.754095	-1.905846	0.302384
N	0.423463	-2.332141	0.166716
O	-1.310611	0.650732	-0.608348
C	-1.675647	1.924488	-0.273788
C	-0.918907	2.776385	0.533107
C	-2.889012	2.361214	-0.816644
C	-1.393341	4.064156	0.797563
H	0.031917	2.465376	0.950680
C	-3.343846	3.646882	-0.548825
H	-3.457720	1.677130	-1.438316
C	-2.599599	4.507528	0.263197
H	-0.800788	4.722166	1.425468
H	-4.287480	3.976581	-0.972198
H	-2.956989	5.509885	0.473247
C	-1.919702	-1.967142	0.102171
C	-2.266167	-3.104284	-0.629006
N	-2.787848	-1.281904	0.858754
C	-3.584244	-3.547391	-0.575217
H	-1.515252	-3.614333	-1.221650
C	-4.044848	-1.727422	0.911767
C	-4.494478	-2.847254	0.211093
H	-3.893976	-4.422090	-1.137630
H	-4.727665	-1.162222	1.540661
H	-5.531703	-3.154223	0.283900
C	3.518699	-0.354193	-0.047767
C	4.496681	-1.303349	0.271376
N	3.809202	0.923275	-0.326248
C	5.824843	-0.898551	0.314431
H	4.205170	-2.327023	0.472194
C	5.090299	1.298112	-0.286250
C	6.133430	0.430261	0.030765
H	6.607480	-1.608872	0.559848
H	5.292007	2.340654	-0.517063
H	7.155844	0.790274	0.049565

Structure **int2_f**

C	0.648185	0.028781	-0.571414
C	1.327939	-1.216465	-0.048264
C	-1.378147	-1.321307	-0.218300
C	-0.687973	-0.381575	-1.169914
H	1.298077	0.490766	-1.316161
H	-1.306025	0.498009	-1.361191
H	-0.512884	-0.880178	-2.132815
N	-0.725795	-2.137620	0.528731
N	0.664634	-2.220573	0.396584
O	0.494013	0.888024	0.553767
C	0.248663	2.218806	0.340350
C	0.177465	2.821485	-0.917302
C	0.073716	2.984164	1.498822
C	-0.063414	4.196145	-1.000895
H	0.301307	2.250970	-1.830246
C	-0.165187	4.349114	1.397870
H	0.132871	2.490228	2.463274
C	-0.235232	4.965955	0.145265
H	-0.117633	4.658871	-1.981337
H	-0.297017	4.934569	2.302401
H	-0.423032	6.031408	0.067608
C	2.813112	-1.288472	-0.052751
C	3.483998	-2.402960	0.463224
N	3.450709	-0.226020	-0.562070
C	4.872817	-2.411542	0.434232
H	2.915988	-3.228673	0.874433
C	4.785713	-0.243893	-0.576664
C	5.542707	-1.310472	-0.095609
H	5.424715	-3.261197	0.822556
H	5.272389	0.633508	-0.993860
H	6.625369	-1.272809	-0.135973
C	-2.866389	-1.313931	-0.111892
C	-3.636068	-0.868444	-1.190498
N	-3.410779	-1.762641	1.028871
C	-5.024462	-0.901469	-1.084466
H	-3.165500	-0.517382	-2.102324
C	-4.739543	-1.776027	1.122037
C	-5.592863	-1.360996	0.096931
H	-5.645188	-0.571814	-1.910919
H	-5.149386	-2.134705	2.062816
H	-6.667765	-1.399172	0.232214

Structure **int2_g**

C	0.880903	-0.801536	-1.169864
C	1.558882	-0.751473	0.184045
C	-1.135271	-1.105305	0.253527
C	-0.447413	-1.526440	-1.016037
H	1.528579	-1.349604	-1.852511
H	-1.070797	-1.350112	-1.894568
H	-0.244852	-2.604864	-0.967741
N	-0.478486	-0.786039	1.310674
N	0.919791	-0.833383	1.296245
O	0.774150	0.498064	-1.763683
C	0.022951	1.432697	-1.071858
C	-1.316860	1.618320	-1.412634
C	0.621229	2.205824	-0.077777
C	-2.073035	2.566605	-0.725338

H	-1.752967	1.022319	-2.208929
C	-0.144217	3.149080	0.606737
H	1.673683	2.064289	0.150375
C	-1.491823	3.327115	0.289753
H	-3.118146	2.707578	-0.982939
H	0.316098	3.749681	1.384836
H	-2.083354	4.062478	0.825269
C	3.039878	-0.619939	0.239609
C	3.682049	-0.249698	1.426638
N	3.700581	-0.854552	-0.902602
C	5.067563	-0.140604	1.425678
H	3.093678	-0.049910	2.314210
C	5.030821	-0.743563	-0.890072
C	5.761274	-0.393743	0.244628
H	5.596709	0.144678	2.329007
H	5.537223	-0.943136	-1.830605
H	6.841534	-0.317415	0.195348
C	-2.625618	-1.051908	0.313210
C	-3.386564	-1.795782	-0.593133
N	-3.181032	-0.281420	1.260271
C	-4.776250	-1.738110	-0.511663
H	-2.909977	-2.420830	-1.339944
C	-4.509838	-0.226593	1.320553
C	-5.355026	-0.932784	0.460747
H	-5.389470	-2.312342	-1.198105
H	-4.927854	0.414507	2.092691
H	-6.431085	-0.847052	0.560482

Structure **int2_h**

C	0.002708	-0.051869	-1.108946
C	0.445907	-1.168755	-0.178939
C	-2.170543	-0.440845	-0.002879
C	-1.254243	0.612287	-0.559782
H	-0.228782	-0.516506	-2.075113
H	-1.020404	1.334125	0.231838
H	-1.764735	1.165992	-1.347373
N	-1.761033	-1.568426	0.456185
N	-0.382914	-1.833060	0.539842
O	1.010489	0.891503	-1.424007
C	1.677583	1.592671	-0.456668
C	2.608152	2.515068	-0.951349
C	1.513686	1.444094	0.923645
C	3.368306	3.279281	-0.075108
H	2.719394	2.611620	-2.026506
C	2.287277	2.219439	1.792446
H	0.805637	0.740623	1.345051
C	3.213188	3.136820	1.306819
H	4.086556	3.988899	-0.473673
H	2.152897	2.094099	2.862294
H	3.806371	3.733359	1.991456
C	1.876343	-1.576217	-0.117959
C	2.448706	-1.981068	1.090582
N	2.543966	-1.546779	-1.277708
C	3.784334	-2.366483	1.091821
H	1.853084	-1.977839	1.996627
C	3.823694	-1.930048	-1.262797
C	4.490049	-2.340082	-0.108819
H	4.266880	-2.677840	2.012438
H	4.341582	-1.906799	-2.217886
H	5.533942	-2.628778	-0.156819
C	-3.637423	-0.177341	0.035250
C	-4.524486	-1.111608	0.582039
N	-4.037663	0.997460	-0.467872
C	-5.880599	-0.810590	0.592725
H	-4.142805	-2.041462	0.985557
C	-5.343142	1.277633	-0.444565
C	-6.304144	0.409508	0.069759
H	-6.595675	-1.514291	1.006006
H	-5.634533	2.239395	-0.858004
H	-7.351927	0.687635	0.059380

Structure **int2_i**

C	-0.021764	-0.026833	-1.044108
C	0.428191	-1.209044	-0.200325
C	-2.172223	-0.452531	0.028670
C	-1.213784	0.635987	-0.367422
H	-0.350780	-0.423835	-2.013743
H	-0.921175	1.203201	0.524119
H	-1.702474	1.331214	-1.049440
N	-1.789213	-1.638455	0.342942
N	-0.418159	-1.919927	0.450262
O	1.006698	0.883112	-1.389507
C	1.685453	1.607617	-0.442161
C	2.486485	2.630332	-0.962017
C	1.656964	1.374290	0.935048
C	3.254359	3.415327	-0.110288
H	2.492590	2.789286	-2.035487
C	2.433169	2.173842	1.778753
H	1.056595	0.584273	1.370805
C	3.231804	3.193026	1.269095
H	3.871872	4.205166	-0.526262
H	2.405018	1.985216	2.847318
H	3.829288	3.806770	1.934551
C	1.864353	-1.607358	-0.140639
C	2.659672	-1.529517	-1.287015
N	2.320917	-2.052147	1.037779
C	3.987416	-1.940573	-1.203872
H	2.247919	-1.157463	-2.218327
C	3.597018	-2.432990	1.104418
C	4.471133	-2.398770	0.016436
H	4.629722	-1.899683	-2.077296
H	3.942891	-2.779578	2.074801
H	5.499501	-2.721506	0.132952
C	-3.635113	-0.170894	0.052720
C	-4.545047	-1.131123	0.509900
N	-4.010697	1.042232	-0.372624
C	-5.897960	-0.815904	0.509003
H	-4.182795	-2.091220	0.856586
C	-5.313554	1.334950	-0.361551
C	-6.295973	0.443277	0.064568
H	-6.630088	-1.538556	0.854102
H	-5.584893	2.327368	-0.711293
H	-7.340293	0.733851	0.048868

Structure **int2_j**

C	-0.346590	0.014923	0.183725
C	-0.825276	-1.423694	0.144818
C	1.828163	-0.987770	-0.151984
C	0.904683	0.075719	-0.679671
H	-0.099433	0.240278	1.233826
H	0.641457	-0.135459	-1.725650
H	1.397951	1.046194	-0.643196
N	1.384217	-2.093333	0.332715
N	0.020148	-2.385877	0.191604
O	-1.374860	0.865115	-0.268884
C	-1.291225	2.207888	-0.007396

C	-0.272262	2.812002	0.730458
C	-2.336143	2.976778	-0.530873
C	-0.314062	4.193374	0.941936
H	0.551620	2.239618	1.140476
C	-2.361648	4.348612	-0.312671
H	-3.114767	2.478911	-1.100038
C	-1.349342	4.967213	0.426917
H	0.480929	4.658912	1.515914
H	-3.176602	4.937678	-0.721406
H	-1.369439	6.038459	0.595714
C	-2.270984	-1.793356	0.061185
C	-3.079838	-1.315263	-0.974474
N	-2.712005	-2.638144	1.002406
C	-4.400616	-1.746984	-1.037706
H	-2.676661	-0.630247	-1.710459
C	-3.987482	-3.031172	0.936533
C	-4.869638	-2.620550	-0.060940
H	-5.050935	-1.404792	-1.836040
H	-4.319034	-3.708123	1.719403
H	-5.892981	-2.978472	-0.065595
C	3.299432	-0.762337	-0.135428
C	4.181462	-1.813623	0.139538
N	3.713043	0.483745	-0.401795
C	5.545340	-1.548846	0.152249
H	3.790789	-2.805944	0.329653
C	5.026586	0.723339	-0.390556
C	5.982084	-0.253624	-0.117024
H	6.256471	-2.340971	0.362264
H	5.329426	1.743790	-0.609121
H	7.036404	-0.001263	-0.120983

Structure **int2_k**

C	-0.150506	0.096544	-0.002998
C	-0.375567	-1.399837	0.093826
C	2.210654	-0.608229	-0.092600
C	1.185885	0.318291	-0.693864
H	-0.155893	0.516551	1.011303
H	1.066679	0.093568	-1.763202
H	1.493663	1.361486	-0.603809
N	1.905983	-1.772323	0.353841
N	0.586663	-2.238641	0.210306
O	-1.186584	0.661124	-0.787401
C	-1.836389	1.777318	-0.332135
C	-1.228785	2.749924	0.462802
C	-3.159621	1.930815	-0.752435
C	-1.964028	3.874014	0.845706
H	-0.193677	2.652675	0.773705
C	-3.876568	3.060734	-0.372235
H	-3.605961	1.153270	-1.363998
C	-3.284523	4.037093	0.433111
H	-1.489104	4.629114	1.464207
H	-4.905564	3.174177	-0.698944
H	-3.846987	4.914868	0.732973
C	-1.765286	-1.928095	0.081913
C	-2.073015	-3.132674	-0.552357
N	-2.667983	-1.186280	0.737636
C	-3.388539	-3.583928	-0.507235
H	-1.295361	-3.686205	-1.066287
C	-3.922828	-1.637833	0.782860
C	-4.335266	-2.823293	0.173310
H	-3.669245	-4.510923	-0.996388
H	-4.634401	-1.022510	1.327462
H	-5.372225	-3.133941	0.233363
C	3.639608	-0.184107	-0.020156
C	4.126819	0.768404	-0.919446
N	4.411792	-0.764071	0.910053
C	5.472891	1.121420	-0.859223
H	3.476556	1.214865	-1.663685
C	5.693659	-0.405893	0.966577
C	6.276487	0.526909	0.105503
H	5.880126	1.849001	-1.553287
H	6.290402	-0.884819	1.738674
H	7.327980	0.773578	0.198716

Structure **int2_l**

C	0.658552	0.025563	-0.567513
C	1.361748	-1.198649	-0.015669
C	-1.344944	-1.343584	-0.177524
C	-0.669294	-0.423147	-1.157413
H	1.284558	0.480129	-1.340592
H	-1.299595	0.441442	-1.375201
H	-0.483846	-0.946611	-2.104900
N	-0.680292	-2.122231	0.597572
N	0.712941	-2.193533	0.466059
O	0.494530	0.916425	0.529593
C	0.195366	2.232181	0.278747
C	0.120138	2.799138	-0.994658
C	-0.021685	3.017590	1.415858
C	-0.169573	4.161674	-1.115912
H	0.276157	2.211756	-1.891847
C	-0.308526	4.369934	1.277292
H	0.042699	2.549636	2.392786
C	-0.384280	4.952145	0.008542
H	-0.227624	4.597661	-2.108209
H	-0.473772	4.972552	2.164814
H	-0.609777	6.007709	-0.098130
C	2.849882	-1.246824	-0.033525
C	3.576022	-0.051949	-0.024466
N	3.434397	-2.453078	-0.035599
C	4.967063	-0.114468	-0.007578
H	3.067237	0.906642	-0.008715
C	4.766161	-2.497541	-0.031068
C	5.579343	-1.361968	-0.014555
H	5.556844	0.795880	0.010637
H	5.211507	-3.489025	-0.043441
H	6.658579	-1.463775	-0.008848
C	-2.833408	-1.357104	-0.074464
C	-3.607849	-0.965820	-1.170358
N	-3.372172	-1.771619	1.081619
C	-4.995715	-1.018785	-1.065341
H	-3.141344	-0.641804	-2.094234
C	-4.700718	-1.804029	1.173294
C	-5.558678	-1.442816	0.131830
H	-5.620198	-0.731658	-1.904706
H	-5.106367	-2.133643	2.126423
H	-6.633062	-1.494310	0.266750

Structure **int2_m**

C	-0.892413	-0.579173	1.263427
C	-1.581027	-0.735620	-0.083387
C	1.113421	-1.123255	-0.096456
C	0.420555	-1.345905	1.219319
H	-1.530277	-1.012154	2.035585
H	1.045247	-1.051699	2.064670
H	0.199365	-2.416059	1.329277
N	0.459645	-0.942450	-1.187389

N	-0.939694	-0.975681	-1.169596
O	-0.754024	0.789774	1.661452
C	0.021524	1.597840	0.845941
C	1.361776	1.804938	1.170952
C	-0.554119	2.225733	-0.257193
C	2.142827	2.625686	0.358746
H	1.778688	1.325038	2.051467
C	0.236346	3.041949	-1.065441
H	-1.607410	2.071790	-0.472906
C	1.585010	3.238165	-0.763944
H	3.188629	2.783026	0.603544
H	-0.205173	3.529245	-1.928910
H	2.195992	3.873918	-1.396362
C	-3.068338	-0.638272	-0.141527
C	-3.751792	0.072121	0.851120
N	-3.694129	-1.233817	-1.167498
C	-5.138711	0.171615	0.769478
H	-3.208204	0.552853	1.657588
C	-5.021514	-1.138137	-1.227055
C	-5.792208	-0.447448	-0.288868
H	-5.693478	0.723654	1.520892
H	-5.499853	-1.639705	-2.064518
H	-6.870277	-0.403893	-0.394058
C	2.604175	-1.107154	-0.163069
C	3.354777	-1.723802	0.841886
N	3.169629	-0.493869	-1.213395
C	4.745030	-1.703132	0.751951
H	2.870398	-2.224374	1.672768
C	4.498915	-0.471393	-1.281558
C	5.334548	-1.058681	-0.327910
H	5.350423	-2.181245	1.514779
H	4.925641	0.042578	-2.139289
H	6.411630	-1.006904	-0.439468

Structure **int2_n**

C	-0.015734	-0.073991	-1.071972
C	0.427983	-1.165250	-0.112752
C	-2.186384	-0.416043	0.045395
C	-1.247775	0.626141	-0.508475
H	-0.284587	-0.566178	-2.014756
H	-0.977878	1.336460	0.282116
H	-1.730623	1.193652	-1.305817
N	-1.782465	-1.531417	0.534280
N	-0.403496	-1.794667	0.633676
O	0.993588	0.843036	-1.446466
C	1.701768	1.566598	-0.525050
C	2.628893	2.456440	-1.081629
C	1.580015	1.468791	0.863838
C	3.427982	3.238781	-0.257601
H	2.706885	2.513293	-2.162502
C	2.392396	2.261914	1.679694
H	0.877005	0.790835	1.332846
C	3.315347	3.147156	1.132689
H	4.143272	3.922554	-0.703806
H	2.290761	2.175884	2.756972
H	3.939022	3.757854	1.776536
C	1.854027	-1.587602	-0.049974
C	2.428202	-1.969232	1.165319
N	2.514704	-1.596296	-1.213876
C	3.758949	-2.370773	1.168765
H	1.837840	-1.936566	2.074195
C	3.789760	-1.994942	-1.196446
C	4.457826	-2.383603	-0.036158
H	4.243004	-2.664882	2.094246
H	4.302016	-2.002814	-2.154787
H	5.497735	-2.686533	-0.082651
C	-3.655299	-0.148538	0.042991
C	-4.119714	1.169476	0.019801
N	-4.478642	-1.206221	0.082444
C	-5.493390	1.397817	0.054561
H	-3.427190	2.003430	-0.009358
C	-5.790136	-0.973857	0.100136
C	-6.350904	0.305617	0.092034
H	-5.880890	2.411115	0.049641
H	-6.431060	-1.851581	0.120248
H	-7.427603	0.430002	0.111944

Structure **int2_o**

C	-0.024350	-0.075812	-1.029436
C	0.425722	-1.212356	-0.126567
C	-2.177801	-0.442118	0.062379
C	-1.208735	0.627300	-0.377497
H	-0.363717	-0.521171	-1.974135
H	-0.900435	1.230733	0.484491
H	-1.671735	1.296060	-1.104855
N	-1.792741	-1.604245	0.448346
N	-0.419701	-1.878985	0.569759
O	1.002290	0.812015	-1.429216
C	1.693412	1.581101	-0.525958
C	2.491378	2.573496	-1.105386
C	1.678387	1.417674	0.861350
C	3.270389	3.397910	-0.302416
H	2.486537	2.678132	-2.185500
C	2.465817	2.256184	1.655461
H	1.080769	0.652865	1.343410
C	3.261828	3.245510	1.086580
H	3.885754	4.163543	-0.764221
H	2.448472	2.121669	2.732395
H	3.868261	3.889856	1.713908
C	1.859876	-1.616764	-0.058842
C	2.646074	-1.601950	-1.213907
N	2.322638	-2.006284	1.136567
C	3.971476	-2.018679	-1.120750
H	2.229538	-1.273940	-2.159525
C	3.596218	-2.393456	1.212026
C	4.461640	-2.419858	0.116782
H	4.606877	-2.025920	-2.000109
H	3.947335	-2.694490	2.195602
H	5.488414	-2.744740	0.241061
C	-3.643147	-0.157626	0.053053
C	-4.093160	1.162730	0.139192
N	-4.478258	-1.204595	-0.017692
C	-5.464814	1.403918	0.167933
H	-3.391405	1.987328	0.199104
C	-5.787231	-0.958517	-0.004702
C	-6.334280	0.323382	0.090769
H	-5.841301	2.418318	0.246599
H	-6.437643	-1.826630	-0.074871
H	-7.409798	0.458623	0.101142

Structure **int2_p**

C	-0.348989	0.014062	0.186037
C	-0.821148	-1.424522	0.121128
C	1.837784	-0.973496	-0.150122
C	0.907775	0.096020	-0.669972
H	-0.108879	0.223286	1.240691

H	0.646362	-0.087122	-1.721515
H	1.368755	1.081993	-0.599980
N	1.393360	-2.090499	0.302436
N	0.028427	-2.383096	0.150894
O	-1.374839	0.867763	-0.262755
C	-1.305066	2.206246	0.026872
C	-0.312567	2.799926	0.807589
C	-2.336769	2.980304	-0.514395
C	-0.367334	4.176849	1.043874
H	0.499836	2.223391	1.234340
C	-2.375791	4.347484	-0.270610
H	-3.094639	2.490166	-1.117220
C	-1.389816	4.955948	0.511704
H	0.406976	4.634429	1.651469
H	-3.180449	4.940866	-0.693193
H	-1.420353	6.023616	0.700312
C	-2.265605	-1.798386	0.033533
C	-3.078770	-1.306228	-0.991996
N	-2.700441	-2.661415	0.960989
C	-4.397672	-1.743249	-1.059861
H	-2.680685	-0.606876	-1.717125
C	-3.974145	-3.059166	0.891225
C	-4.860400	-2.635567	-0.097260
H	-5.051341	-1.390540	-1.850831
H	-4.300762	-3.750985	1.663081
H	-5.882071	-2.998166	-0.105881
C	3.311056	-0.738902	-0.127567
C	3.882528	0.166640	-1.025777
N	4.036656	-1.439431	0.755757
C	5.264525	0.340787	-1.014688
H	3.267590	0.713790	-1.731999
C	5.355376	-1.251362	0.766356
C	6.020713	-0.378428	-0.097709
H	5.735816	1.028110	-1.709272
H	5.914283	-1.824576	1.501651
H	7.098145	-0.272151	-0.043200

Structure **int3_a**

C	-0.355423	0.044551	-0.064390
C	-0.719106	-1.210017	0.252746
C	1.947521	-0.620657	0.333020
C	1.000351	0.557916	0.326726
H	1.340823	1.312830	-0.386067
H	0.996875	1.036014	1.318268
N	1.572164	-1.817095	0.617122
N	0.250971	-2.029872	0.851816
O	-1.150510	0.843785	-0.852352
C	-1.497978	2.081502	-0.348165
C	-1.610859	2.318658	1.020795
C	-1.790107	3.077000	-1.279388
C	-2.009931	3.582855	1.457061
H	-1.401184	1.524361	1.731289
C	-2.194087	4.330752	-0.828736
H	-1.695871	2.855930	-2.337479
C	-2.301526	4.590787	0.539523
H	-2.097345	3.772836	2.522063
H	-2.421502	5.108767	-1.550448
H	-2.612177	5.570713	0.885898
H	0.009733	-3.014015	0.842626
C	-2.029492	-1.861593	0.014199
C	-3.211198	-1.135079	-0.184640
N	-2.012619	-3.202891	0.049617
C	-4.395908	-1.834165	-0.381163
H	-3.202053	-0.052796	-0.176580
C	-3.159127	-3.861855	-0.136141
C	-4.376777	-3.227129	-0.363965
H	-5.324864	-1.295630	-0.537881
H	-3.098545	-4.946211	-0.099355
H	-5.279128	-3.809151	-0.511792
C	3.389401	-0.396229	0.060503
C	4.291355	-1.466586	-0.031976
N	3.771521	0.880650	-0.077967
C	5.629878	-1.191422	-0.270980
H	3.931971	-2.482279	0.081578
C	5.064801	1.130253	-0.311568
C	6.032907	0.136684	-0.415830
H	6.350337	-1.999472	-0.348101
H	5.336882	2.177171	-0.418769
H	7.067696	0.397749	-0.606343

Structure **int3_b**

C	-0.450792	-0.080450	-0.971422
C	-0.998413	-1.215562	-0.506127
C	1.733198	-1.027532	-0.450710
C	1.006971	-0.020651	-1.313455
H	1.177095	-0.239507	-2.378965
H	1.403445	0.982408	-1.134167
N	1.178402	-2.101314	-0.014216
N	-0.134586	-2.307076	-0.303202
O	-1.198975	1.047686	-1.223924
C	-1.050477	2.130527	-0.380820
C	-0.400736	2.053120	0.849599
C	-1.620788	3.326347	-0.818952
C	-0.320072	3.199041	1.643754
H	0.029766	1.115271	1.186648
C	-1.537326	4.456962	-0.012354
H	-2.120322	3.351096	-1.782016
C	-0.884256	4.400722	1.221634
H	0.187952	3.142174	2.601306
H	-1.982439	5.386955	-0.351543
H	-0.817659	5.285353	1.845807
H	-0.544154	-3.044016	0.258825
C	-2.415516	-1.469724	-0.141981
C	-3.467529	-0.612421	-0.493445
N	-2.621926	-2.605753	0.543529
C	-4.757502	-0.947065	-0.097010
H	-3.277605	0.285149	-1.065511
C	-3.865783	-2.915747	0.914359
C	-4.969837	-2.118380	0.625274
H	-5.588498	-0.298879	-0.355669
H	-3.982456	-3.844323	1.466698
H	-5.960346	-2.413723	0.951691
C	3.164796	-0.807772	-0.126477
C	3.855797	-1.656050	0.751184
N	3.749596	0.237573	-0.727453
C	5.197000	-1.405453	1.001998
H	3.336027	-2.484563	1.217192
C	5.042694	0.468081	-0.473918
C	5.811055	-0.318780	0.378019
H	5.757063	-2.044256	1.677404
H	5.482763	1.324224	-0.978783
H	6.855869	-0.084146	0.546972

Structure int3_c

C	-0.354287	0.047627	-0.071055
C	-0.713366	-1.201924	0.268386

C	1.955119	-0.605508	0.335923
C	0.998961	0.570936	0.318296
H	1.316184	1.320017	-0.414002
H	0.984932	1.064162	1.302932
N	1.580534	-1.794942	0.646518
N	0.259071	-2.002978	0.885452
O	-1.146930	0.834134	-0.873304
C	-1.513621	2.071265	-0.379927
C	-1.635405	2.317898	0.986500
C	-1.814465	3.055066	-1.320729
C	-2.052998	3.580364	1.410559
H	-1.419135	1.532268	1.704617
C	-2.236937	4.306966	-0.882117
H	-1.712888	2.826463	-2.376529
C	-2.353830	4.576718	0.483518
H	-2.147726	3.777760	2.473574
H	-2.471587	5.075928	-1.611165
H	-2.679018	5.555201	0.820480
H	0.020066	-2.987492	0.903839
C	-2.019709	-1.864715	0.037154
C	-3.202473	-1.149352	-0.192409
N	-1.997111	-3.204398	0.109017
C	-4.382249	-1.859232	-0.380622
H	-3.198505	-0.067296	-0.214539
C	-3.138702	-3.873647	-0.069193
C	-4.357057	-3.250995	-0.325295
H	-5.312002	-1.329575	-0.560923
H	-3.073448	-4.956289	-0.002582
H	-5.255353	-3.841041	-0.465779
C	3.398498	-0.378095	0.059326
C	3.939378	0.907085	0.186158
N	4.146010	-1.437257	-0.291693
C	5.297919	1.095928	-0.053515
H	3.317535	1.743635	0.484322
C	5.444745	-1.241635	-0.517234
C	6.073248	0.000734	-0.415223
H	5.738910	2.082508	0.044777
H	6.017182	-2.121143	-0.802077
H	7.134165	0.095930	-0.616557

Structure **int3_d**

C	-0.369494	-0.112225	-0.687628
C	-0.782857	-1.292953	-0.210776
C	1.921613	-0.843316	-0.216012
C	1.077651	0.188257	-0.930947
H	1.301734	0.190693	-2.008175
H	1.337969	1.189841	-0.569772
N	1.485071	-1.966808	0.229995
N	0.176194	-2.284748	0.066805
O	-1.276700	0.821634	-1.138404
C	-1.390823	2.002452	-0.442429
C	-0.895294	2.174628	0.849330
C	-2.062931	3.037297	-1.094438
C	-1.074395	3.403943	1.485727
H	-0.390320	1.356508	1.353537
C	-2.239992	4.254770	-0.443746
H	-2.437540	2.871871	-2.099426
C	-1.744227	4.446772	0.848150
H	-0.688265	3.539127	2.491291
H	-2.764884	5.058200	-0.951004
H	-1.880469	5.398260	1.351035
H	-0.107864	-3.015260	0.706698
C	-2.197178	-1.692526	-0.013970
C	-2.617295	-2.971803	-0.394459
N	-3.018845	-0.793286	0.546625
C	-3.946670	-3.327901	-0.187800
H	-1.919388	-3.661583	-0.857719
C	-4.292183	-1.148399	0.731717
C	-4.807028	-2.397936	0.386205
H	-4.302413	-4.311444	-0.476743
H	-4.934284	-0.398628	1.186903
H	-5.850981	-2.627359	0.567052
C	3.369608	-0.563091	-0.039907
C	4.207092	-1.434653	0.671715
N	3.818577	0.565357	-0.606130
C	5.551342	-1.115058	0.793344
H	3.795244	-2.334819	1.111815
C	5.117529	0.860307	-0.480213
C	6.024330	0.059181	0.205748
H	6.223114	-1.769560	1.339443
H	5.445409	1.782675	-0.952704
H	7.066775	0.348324	0.276259

Structure **int3_e**

C	-0.312834	0.006572	-0.345292
C	-0.669048	-1.226967	0.037143
C	1.999981	-0.623598	0.150009
C	1.070058	0.539619	-0.109294
H	1.412159	1.101267	-0.984992
H	1.102922	1.245349	0.734494
N	1.619411	-1.797225	0.510447
N	0.293762	-2.045305	0.655820
O	-1.202452	0.833303	-0.994667
C	-1.558226	1.999288	-0.349526
C	-1.550883	2.117295	1.039920
C	-1.983350	3.054360	-1.156725
C	-1.965429	3.316801	1.620292
H	-1.234513	1.281032	1.656265
C	-2.401602	4.241999	-0.562792
H	-1.979958	2.928911	-2.234590
C	-2.391241	4.381071	0.827039
H	-1.958030	3.412213	2.701553
H	-2.732918	5.063699	-1.189879
H	-2.713205	5.309849	1.285832
H	0.101447	-3.038741	0.656322
C	-2.030326	-1.809637	-0.062744
C	-2.588374	-2.437570	1.056790
N	-2.664902	-1.721259	-1.239806
C	-3.861721	-2.987772	0.949716
H	-2.037448	-2.475478	1.991003
C	-3.889133	-2.248849	-1.324048
C	-4.531396	-2.890962	-0.265954
H	-4.322531	-3.476162	1.802075
H	-4.379941	-2.160840	-2.289896
H	-5.525450	-3.302224	-0.399815
C	3.462009	-0.397234	0.017648
C	4.383872	-1.445577	0.153977
N	3.838187	0.864832	-0.229931
C	5.736277	-1.163660	0.028872
H	4.029156	-2.449985	0.351343
C	5.145831	1.121146	-0.349359
C	6.133504	0.149057	-0.229088
H	6.472636	-1.954796	0.128002
H	5.413063	2.155478	-0.550037
H	7.179215	0.414424	-0.335549

Structure **int3_f**

C	-0.395953	-0.120823	-0.730925
C	-0.834639	-1.290014	-0.249817
C	1.881213	-0.899626	-0.253250
C	1.055421	0.118032	-1.015463
H	1.250037	0.053904	-2.097584
H	1.334239	1.134308	-0.708991
N	1.418336	-2.011958	0.192606
N	0.105687	-2.305652	0.004930
O	-1.275127	0.852384	-1.149674
C	-1.320239	2.031643	-0.441635
C	-0.776691	2.173500	0.834274
C	-1.969826	3.097694	-1.065387
C	-0.884346	3.404475	1.483661
H	-0.290325	1.331587	1.317350
C	-2.075365	4.316541	-0.401831
H	-2.382843	2.955083	-2.058731
C	-1.530631	4.478447	0.874364
H	-0.460862	3.516224	2.476947
H	-2.582673	5.144597	-0.886852
H	-1.611199	5.431035	1.387050
H	-0.195540	-3.038983	0.633926
C	-2.254729	-1.649516	-0.020611
C	-2.719039	-2.915826	-0.392841
N	-3.037896	-0.727976	0.558176
C	-4.052868	-3.234697	-0.156084
H	-2.051979	-3.624460	-0.872910
C	-4.316192	-1.047304	0.771985
C	-4.873450	-2.281689	0.437947
H	-4.442512	-4.207516	-0.437429
H	-4.926498	-0.280158	1.241685
H	-5.919016	-2.482118	0.642397
C	3.329722	-0.624398	-0.047078
C	3.967526	0.361265	-0.809785
N	3.985259	-1.349615	0.874273
C	5.326599	0.595206	-0.613561
H	3.421677	0.928974	-1.554418
C	5.283882	-1.114307	1.051988
C	6.005648	-0.155167	0.337964
H	5.841032	1.350983	-1.198095
H	5.779286	-1.718058	1.808634
H	7.062231	-0.008690	0.531040

Structure **int3_g**

C	-0.395953	-0.120823	-0.730925
C	-0.834639	-1.290014	-0.249817
C	1.881213	-0.899626	-0.253250
C	1.055421	0.118032	-1.015463
H	1.250037	0.053904	-2.097584
H	1.334239	1.134308	-0.708991
N	1.418336	-2.011958	0.192606
N	0.105687	-2.305652	0.004930
O	-1.275127	0.852384	-1.149674
C	-1.320239	2.031643	-0.441635
C	-0.776691	2.173500	0.834274
C	-1.969826	3.097694	-1.065387
C	-0.884346	3.404475	1.483661
H	-0.290325	1.331587	1.317350
C	-2.075365	4.316541	-0.401831
H	-2.382843	2.955083	-2.058731
C	-1.530631	4.478447	0.874364
H	-0.460862	3.516224	2.476947
H	-2.582673	5.144597	-0.886852
H	-1.611199	5.431035	1.387050
H	-0.195540	-3.038983	0.633926
C	-2.254729	-1.649516	-0.020611
C	-2.719039	-2.915826	-0.392841
N	-3.037896	-0.727976	0.558176
C	-4.052868	-3.234697	-0.156084
H	-2.051979	-3.624460	-0.872910
C	-4.316192	-1.047304	0.771985
C	-4.873450	-2.281689	0.437947
H	-4.442512	-4.207516	-0.437429
H	-4.926498	-0.280158	1.241685
H	-5.919016	-2.482118	0.642397
C	3.329722	-0.624398	-0.047078
C	3.967526	0.361265	-0.809785
N	3.985259	-1.349615	0.874273
C	5.326599	0.595206	-0.613561
H	3.421677	0.928974	-1.554418
C	5.283882	-1.114307	1.051988
C	6.005648	-0.155167	0.337964
H	5.841032	1.350983	-1.198095
H	5.779286	-1.718058	1.808634
H	7.062231	-0.008690	0.531040

Structure **int3_h**

C	-0.304397	0.011290	-0.335160
C	-0.651311	-1.217929	0.067173
C	2.016259	-0.593621	0.176479
C	1.068486	0.561277	-0.078610
H	1.394240	1.135008	-0.954768
H	1.072175	1.259739	0.773160
N	1.640121	-1.760908	0.559610
N	0.315173	-2.008121	0.712932
O	-1.189045	0.819188	-1.012784
C	-1.587516	1.978813	-0.380167
C	-1.609331	2.104354	1.008402
C	-2.024159	3.018062	-1.201626
C	-2.066126	3.295835	1.573691
H	-1.284197	1.280038	1.636148
C	-2.484586	4.197365	-0.622696
H	-1.997333	2.886708	-2.278439
C	-2.504400	4.344147	0.766308
H	-2.081937	3.397053	2.654319
H	-2.825428	5.006637	-1.260722
H	-2.859533	5.266496	1.213407
H	0.127392	-3.001807	0.747379
C	-2.003194	-1.820176	-0.041328
C	-2.569699	-2.433910	1.081674
N	-2.620326	-1.762390	-1.229377
C	-3.833447	-3.004116	0.965532
H	-2.033287	-2.445913	2.025013
C	-3.835734	-2.308632	-1.322198
C	-4.485166	-2.939937	-0.261936
H	-4.300656	-3.482861	1.819867
H	-4.312758	-2.245535	-2.296821
H	-5.471053	-3.368045	-0.403160
C	3.478509	-0.358512	0.025799
C	3.973498	0.949963	-0.031042
N	4.285667	-1.430524	-0.040244
C	5.346402	1.149530	-0.155864
H	3.306055	1.801373	0.034877
C	5.595881	-1.224361	-0.159777
C	6.181835	0.041806	-0.223568
H	5.750843	2.155641	-0.196918

H	6.216266	-2.115826	-0.213432
H	7.256310	0.143909	-0.324597

Structure **int4_a**

C	-0.751296	-0.421060	1.367783
C	-1.362600	-0.757335	0.035537
C	1.350349	-0.853769	0.195902
C	0.680463	-0.816841	1.368099
H	1.187539	-0.991258	2.309040
N	0.653916	-0.734054	-0.985625
N	-0.677218	-0.837424	-1.059473
O	-0.931591	1.011576	1.661697
C	-0.368384	1.892527	0.766008
C	0.939960	2.343859	0.956736
C	-1.125569	2.362048	-0.309574
C	1.494554	3.252751	0.056519
H	1.506831	1.978901	1.807463
C	-0.564411	3.271714	-1.205149
H	-2.146251	2.010761	-0.431544
C	0.747367	3.714325	-1.028628
H	2.511812	3.601892	0.204858
H	-1.154543	3.635941	-2.040415
H	1.182273	4.421457	-1.727648
H	-1.316028	-0.885136	2.173905
H	1.154326	-0.907868	-1.848183
C	-2.835910	-0.914845	-0.075415
C	-3.463194	-1.044394	-1.323354
N	-3.521331	-0.920341	1.076258
C	-4.843069	-1.186640	-1.365511
H	-2.867370	-1.030467	-2.228019
C	-4.849293	-1.064014	1.020212
C	-5.559517	-1.199456	-0.168903
H	-5.354376	-1.285902	-2.317738
H	-5.369345	-1.067549	1.974783
H	-6.637995	-1.308301	-0.153990
C	2.815255	-1.046020	0.037241
C	3.695668	-0.856283	1.105984
N	3.230247	-1.382233	-1.191601
C	5.053898	-1.060797	0.890474
H	3.329063	-0.543256	2.076858
C	4.538030	-1.564887	-1.389348
C	5.489223	-1.426302	-0.380962
H	5.762336	-0.925462	1.700968
H	4.836941	-1.834705	-2.398461
H	6.539101	-1.590776	-0.594281

Structure **int4_b**

C	0.544209	0.035912	-0.437931
C	0.860381	-1.347425	0.059979
C	-1.764879	-0.653962	-0.033625
C	-0.914459	0.130572	-0.730474
H	-1.284957	0.844700	-1.454925
N	-1.261003	-1.646000	0.777833
N	0.001561	-2.083700	0.684605
O	0.939482	0.921068	0.643779
C	1.127742	2.243979	0.374297
C	0.928774	2.839354	-0.875451
C	1.555678	3.025356	1.456463
C	1.166688	4.208467	-1.028768
H	0.590401	2.264835	-1.729224
C	1.786103	4.385008	1.287984
H	1.703621	2.543785	2.417823
C	1.593773	4.988548	0.041494
H	1.009069	4.660776	-2.003131
H	2.119373	4.977067	2.134833
H	1.773435	6.050286	-0.089183
H	1.169993	0.264766	-1.302479
H	-1.919946	-2.306856	1.170433
C	2.239426	-1.875717	-0.093394
C	2.579270	-3.166247	0.336764
N	3.127860	-1.050182	-0.663991
C	3.886796	-3.601074	0.171348
H	1.823953	-3.800145	0.785459
C	4.381956	-1.485074	-0.822171
C	4.815203	-2.745586	-0.421687
H	4.178693	-4.594713	0.496023
H	5.073064	-0.788735	-1.290225
H	5.846340	-3.044130	-0.572643
C	-3.249858	-0.590031	-0.087857
C	-3.916259	0.513954	-0.626840
N	-3.896573	-1.644123	0.427186
C	-5.305989	0.499442	-0.657534
H	-3.363765	1.368721	-0.999111
C	-5.231598	-1.641829	0.403166
C	-5.983054	-0.600100	-0.135500
H	-5.851803	1.339920	-1.073190
H	-5.719901	-2.512110	0.832775
H	-7.065767	-0.649935	-0.134477

Structure **int4_c**

C	-0.745460	-0.330797	1.382026
C	-1.358288	-0.761010	0.074539
C	1.356081	-0.842317	0.245470
C	0.685208	-0.725086	1.411891
H	1.189635	-0.830159	2.364257
N	0.659820	-0.808035	-0.939867
N	-0.670886	-0.914717	-1.011540
O	-0.927326	1.116247	1.597764
C	-0.373794	1.946046	0.647474
C	0.934969	2.409264	0.801264
C	-1.141879	2.349239	-0.446561
C	1.479125	3.265701	-0.154945
H	1.509637	2.095148	1.666872
C	-0.590937	3.207173	-1.397776
H	-2.162782	1.989344	-0.538301
C	0.721086	3.661950	-1.258209
H	2.496672	3.625045	-0.036201
H	-1.188877	3.521060	-2.247706
H	1.148001	4.328505	-2.000645
H	-1.298041	-0.748385	2.225638
H	1.162869	-1.024603	-1.791149
C	-2.832179	-0.933708	-0.017726
C	-3.670367	-0.311591	0.917330
N	-3.311674	-1.694779	-1.014776
C	-5.046768	-0.491377	0.813178
H	-3.251264	0.320542	1.692369
C	-4.632520	-1.851244	-1.105518
C	-5.545459	-1.277994	-0.219204
H	-5.715680	-0.017295	1.524184
H	-4.983883	-2.474928	-1.924034
H	-6.608957	-1.448546	-0.342165
C	2.822083	-1.039419	0.102154
C	3.699627	-0.783443	1.159271
N	3.240425	-1.446429	-1.103812
C	5.059061	-0.994757	0.958302
H	3.330444	-0.414990	2.109482
C	4.549239	-1.634932	-1.288518

C	5.498107	-1.432735	-0.288702
H	5.765390	-0.808986	1.760588
H	4.851002	-1.962767	-2.279427
H	6.549043	-1.605031	-0.490347

Structure **int4_d**

C	0.539901	0.026997	-0.439646
C	0.860394	-1.355045	0.067011
C	-1.767544	-0.657759	-0.022446
C	-0.919400	0.126953	-0.721665
H	-1.290633	0.844265	-1.442156
N	-1.261457	-1.650379	0.783971
N	0.001343	-2.087633	0.695225
O	0.959687	0.926956	0.621051
C	1.130790	2.251333	0.336049
C	0.911955	2.831580	-0.917018
C	1.569104	3.044257	1.405042
C	1.139053	4.200845	-1.086487
H	0.565485	2.247515	-1.761029
C	1.788460	4.403546	1.220382
H	1.732982	2.572300	2.368538
C	1.575260	4.993314	-0.029350
H	0.965530	4.642761	-2.062820
H	2.129357	5.006012	2.056708
H	1.746093	6.054873	-0.172525
H	1.142994	0.250059	-1.325733
H	-1.919123	-2.305803	1.187588
C	2.244740	-1.877358	-0.079681
C	3.309036	-0.994599	-0.305726
N	2.423165	-3.204633	0.012794
C	4.594682	-1.509621	-0.443319
H	3.140222	0.075873	-0.347936
C	3.661283	-3.682438	-0.113539
C	4.781716	-2.883898	-0.346502
H	5.434483	-0.844496	-0.616206
H	3.766591	-4.761663	-0.033555
H	5.763125	-3.333111	-0.447303
C	-3.252766	-0.590400	-0.069971
C	-3.919617	0.512594	-0.610028
N	-3.898557	-1.641020	0.453006
C	-5.309605	0.500995	-0.632397
H	-3.367834	1.364561	-0.989671
C	-5.233598	-1.636139	0.436615
C	-5.985901	-0.594824	-0.101808
H	-5.856013	1.340857	-1.048466
H	-5.721193	-2.503722	0.872397
H	-7.068689	-0.642273	-0.094289

Structure **int4_e**

C	0.742146	-0.651121	-1.265896
C	1.380316	-0.763129	0.090890
C	-1.334213	-0.917267	-0.003343
C	-0.683851	-1.055777	-1.175912
H	-1.235449	-1.329954	-2.066762
N	-0.621499	-0.600632	1.139038
N	0.715864	-0.680513	1.197143
O	0.886822	0.723170	-1.778102
C	0.331772	1.718428	-1.004551
C	-0.994591	2.107964	-1.207143
C	1.114284	2.358286	-0.040958
C	-1.541476	3.125497	-0.426190
H	-1.581847	1.609469	-1.971630
C	0.561232	3.376818	0.734314
H	2.148019	2.050931	0.089993
C	-0.768653	3.757753	0.549415
H	-2.573318	3.425230	-0.582122
H	1.171091	3.873226	1.482767
H	-1.197761	4.549132	1.155563
H	1.303331	-1.224393	-2.001363
H	-1.063055	-0.689518	2.043911
C	2.858767	-0.873165	0.197558
C	3.507863	-0.799987	1.438568
N	3.525303	-1.038572	-0.953146
C	4.891195	-0.906474	1.475344
H	2.926558	-0.660766	2.342066
C	4.856911	-1.145320	-0.901521
C	5.588564	-1.085153	0.280824
H	5.419664	-0.850233	2.421660
H	5.361817	-1.282109	-1.854400
H	6.668895	-1.172894	0.261961
C	-2.806064	-1.048034	0.148109
C	-3.500245	-0.263853	1.074800
N	-3.415640	-1.916079	-0.670825
C	-4.882702	-0.403749	1.163517
H	-2.979337	0.461415	1.691766
C	-4.741054	-2.035406	-0.577165
C	-5.520746	-1.310484	0.324652
H	-5.448625	0.193805	1.870473
H	-5.204942	-2.748395	-1.253694
H	-6.594538	-1.454710	0.357301

Structure **int4_f**

C	0.489128	0.005852	-0.427815
C	0.826129	-1.386427	0.030798
C	-1.815339	-0.733190	-0.033036
C	-0.978221	0.113943	-0.663302
H	-1.392416	0.911491	-1.267450
N	-1.302117	-1.787838	0.700748
N	-0.021794	-2.177787	0.597010
O	0.920137	0.873525	0.653736
C	1.160409	2.188362	0.385320
C	0.936939	2.802594	-0.851018
C	1.665010	2.940781	1.454765
C	1.228327	4.161178	-1.004599
H	0.536725	2.251007	-1.693202
C	1.946893	4.290814	1.286560
H	1.830420	2.444910	2.405953
C	1.731780	4.912678	0.052928
H	1.051021	4.628470	-1.968443
H	2.338888	4.860769	2.123379
H	1.952237	5.966725	-0.077554
H	1.087340	0.251964	-1.307592
H	-1.915917	-2.533953	0.996355
C	2.225021	-1.868129	-0.107806
C	2.608991	-3.138491	0.344167
N	3.085444	-1.020658	-0.688723
C	3.930861	-3.530530	0.185982
H	1.876497	-3.790164	0.804944
C	4.354096	-1.414146	-0.839204
C	4.829898	-2.652850	-0.419594
H	4.256805	-4.507663	0.527885
H	5.021555	-0.701169	-1.316376
H	5.871176	-2.917434	-0.564090
C	-3.296941	-0.609508	-0.068794
C	-4.085119	-1.063670	0.993009
N	-3.814973	-0.020141	-1.155133

C	-5.466831	-0.912575	0.911039
H	-3.634905	-1.497530	1.879661
C	-5.139469	0.122690	-1.217235
C	-6.011101	-0.310956	-0.217790
H	-6.102459	-1.252740	1.721810
H	-5.526221	0.603804	-2.111744
H	-7.080522	-0.172255	-0.326999

Structure **int4_g**

C	-0.736771	-0.551163	1.303260
C	-1.377220	-0.766576	-0.043077
C	1.338483	-0.909904	0.067070
C	0.686914	-0.965021	1.246087
H	1.234715	-1.175817	2.156114
N	0.626303	-0.675488	-1.093071
N	-0.710525	-0.759535	-1.151191
O	-0.880039	0.853475	1.728649
C	-0.333676	1.796188	0.884450
C	0.994891	2.196467	1.045942
C	-1.127427	2.368580	-0.111297
C	1.533071	3.157424	0.190849
H	1.590408	1.751208	1.836393
C	-0.582835	3.331171	-0.960433
H	-2.162651	2.054063	-0.208982
C	0.749274	3.721906	-0.817050
H	2.566727	3.465944	0.313915
H	-1.200806	3.775507	-1.734438
H	1.171854	4.469072	-1.481079
H	-1.286359	-1.077017	2.086130
H	1.071907	-0.809025	-1.990426
C	-2.856011	-0.897521	-0.135644
C	-3.666657	-0.414888	0.900219
N	-3.365325	-1.483066	-1.231395
C	-5.047986	-0.550762	0.793756
H	-3.223120	0.080011	1.757116
C	-4.690278	-1.599654	-1.321638
C	-5.577819	-1.156152	-0.340109
H	-5.696140	-0.181888	1.582101
H	-5.066698	-2.080130	-2.221505
H	-6.646345	-1.285508	-0.469467
C	2.810320	-1.052850	-0.073067
C	3.506786	-0.332651	-1.048676
N	3.416891	-1.865956	0.802194
C	4.888938	-0.481324	-1.126765
H	2.988100	0.351911	-1.712230
C	4.742144	-1.994363	0.717772
C	5.524054	-1.332490	-0.229361
H	5.456866	0.067020	-1.871029
H	5.203773	-2.662173	1.440329
H	6.597502	-1.481158	-0.251621

Structure **int4_h**

C	0.492754	0.001543	-0.422131
C	0.842261	-1.383531	0.057136
C	-1.804623	-0.741319	-0.011176
C	-0.973833	0.098322	-0.659667
H	-1.391640	0.880013	-1.281239
N	-1.282263	-1.776016	0.740727
N	-0.001329	-2.164737	0.643530
O	0.928881	0.895446	0.636831
C	1.139482	2.213099	0.345706
C	0.893648	2.802647	-0.897877
C	1.645111	2.987955	1.397991
C	1.162195	4.163253	-1.075313
H	0.492483	2.232683	-1.727247
C	1.903802	4.339346	1.205952
H	1.828815	2.508763	2.354314
C	1.665207	4.938087	-0.034836
H	0.967216	4.612822	-2.044054
H	2.296205	4.928305	2.029216
H	1.867546	5.993275	-0.183897
H	1.073385	0.242019	-1.319352
H	-1.891525	-2.513084	1.066661
C	2.244351	-1.862004	-0.084024
C	3.282871	-0.947788	-0.304323
N	2.461972	-3.183895	0.002163
C	4.583288	-1.424809	-0.442788
H	3.084720	0.117586	-0.342590
C	3.713223	-3.625011	-0.124794
C	4.810525	-2.793128	-0.352043
H	5.403209	-0.734357	-0.611708
H	3.849520	-4.701137	-0.049743
H	5.804839	-3.212933	-0.453128
C	-3.287052	-0.627460	-0.048781
C	-4.072613	-1.070448	1.019454
N	-3.807936	-0.057793	-1.144126
C	-5.455284	-0.929809	0.934284
H	-3.620745	-1.487498	1.913275
C	-5.133255	0.075078	-1.209411
C	-6.002652	-0.349301	-0.203980
H	-6.089160	-1.261776	1.749791
H	-5.522550	0.540076	-2.111288
H	-7.072905	-0.219564	-0.315997

Structure **TS3_a**

C	-0.893653	-1.109524	1.129795
C	-1.504439	-0.795998	-0.129271
C	1.198099	-1.010097	-0.012235
C	0.483520	-1.354693	1.119715
H	0.989191	-1.712291	2.006593
N	0.516055	-0.625189	-1.107402
N	-0.801366	-0.536674	-1.204239
O	-1.046567	0.820098	1.965659
C	-0.259847	1.572790	1.242056
C	1.138988	1.637535	1.488514
C	-0.749991	2.283112	0.111466
C	1.993544	2.317592	0.624464
H	1.529234	1.121682	2.362654
C	0.111881	2.960154	-0.741681
H	-1.820132	2.258238	-0.081650
C	1.493253	2.966289	-0.507356
H	3.062205	2.327588	0.826734
H	-0.290148	3.479909	-1.607752
H	2.163316	3.486198	-1.184826
H	-1.522910	-1.450142	1.938164
H	1.047941	-0.395562	-1.943777
C	-2.981552	-0.682726	-0.249725
C	-3.569486	-0.062413	-1.356682
N	-3.692968	-1.198751	0.759914
C	-4.956128	0.017314	-1.411852
H	-2.945888	0.348302	-2.142022
C	-5.023931	-1.117490	0.692499
C	-5.704270	-0.521212	-0.367786
H	-5.444360	0.495916	-2.254440
H	-5.572619	-1.548503	1.525649
H	-6.787553	-0.480964	-0.367690

C	2.674766	-1.024332	-0.158334
C	3.518677	-1.276160	0.923039
N	3.124431	-0.740635	-1.387381
C	4.893019	-1.236722	0.706178
H	3.123507	-1.484918	1.910044
C	4.442088	-0.702401	-1.583298
C	5.368088	-0.944744	-0.568373
H	5.579640	-1.426008	1.524171
H	4.771988	-0.468580	-2.591289
H	6.430308	-0.900568	-0.778875

Structure **TS3_b**

C	-0.891523	-0.700653	1.401834
C	-1.512679	-0.784081	0.110660
C	1.191889	-0.965066	0.272853
C	0.484143	-0.948523	1.458948
H	0.991668	-1.032120	2.410476
N	0.501540	-0.925822	-0.882856
N	-0.815418	-0.867058	-0.996908
O	-0.998458	1.386806	1.690010
C	-0.225302	1.890518	0.762748
C	1.177614	2.015125	0.953322
C	-0.737730	2.238675	-0.517046
C	2.015522	2.407485	-0.087569
H	1.584593	1.774867	1.932733
C	0.107681	2.630166	-1.547265
H	-1.811474	2.163531	-0.673902
C	1.493068	2.698511	-1.350184
H	3.087879	2.469927	0.082967
H	-0.310688	2.873554	-2.520624
H	2.149657	2.993423	-2.162672
H	-1.501607	-0.795933	2.289706
H	1.028817	-0.956028	-1.752777
C	-2.993217	-0.728593	-0.030881
C	-3.748371	0.017665	0.878639
N	-3.536123	-1.411220	-1.048081
C	-5.131423	0.049264	0.722574
H	-3.245616	0.585033	1.654422
C	-4.862575	-1.368680	-1.184765
C	-5.704966	-0.658880	-0.328130
H	-5.747207	0.624125	1.406519
H	-5.275859	-1.933973	-2.016089
H	-6.777071	-0.665424	-0.489069
C	2.667285	-1.031366	0.130130
C	3.517019	-0.950896	1.232978
N	3.109526	-1.133754	-1.129575
C	4.889930	-0.990382	1.007701
H	3.127493	-0.848531	2.238836
C	4.426074	-1.166088	-1.334378
C	5.357617	-1.099784	-0.297997
H	5.581235	-0.930072	1.841267
H	4.750300	-1.248332	-2.367638
H	6.418545	-1.130013	-0.517331

Structure **TS3_c**

C	0.839238	-1.192629	-1.000579
C	1.483234	-0.786351	0.214221
C	-1.225943	-0.946880	0.172036
C	-0.543009	-1.389331	-0.945564
H	-1.106247	-1.782079	-1.781689
N	-0.512313	-0.494805	1.223098
N	0.812261	-0.423741	1.278700
O	1.052612	0.670757	-1.994713
C	0.298500	1.504189	-1.329691
C	-1.097889	1.605458	-1.580217
C	0.820453	2.282962	-0.259367
C	-1.918020	2.399939	-0.783585
H	-1.512073	1.034575	-2.407665
C	-0.009253	3.067723	0.531466
H	1.889089	2.229701	-0.063827
C	-1.388186	3.120225	0.290391
H	-2.984475	2.444627	-0.991068
H	0.416694	3.639873	1.351903
H	-2.033159	3.728795	0.916646
H	1.444124	-1.608245	-1.792291
H	-0.972020	-0.226104	2.085616
C	2.965257	-0.699242	0.295461
C	3.590880	0.008262	1.326427
N	3.641608	-1.328565	-0.672978
C	4.980108	0.056064	1.347844
H	2.994247	0.508983	2.079910
C	4.975197	-1.277023	-0.638351
C	5.692175	-0.599954	0.346824
H	5.497860	0.600134	2.130955
H	5.495468	-1.800684	-1.435852
H	6.775881	-0.588729	0.322618
C	-2.704099	-0.963272	0.287278
C	-3.380782	-0.049425	1.097539
N	-3.324907	-1.886957	-0.457548
C	-4.770627	-0.120538	1.152879
H	-2.850284	0.726514	1.640163
C	-4.655030	-1.939956	-0.397936
C	-5.423548	-1.085824	0.395936
H	-5.328960	0.576000	1.768955
H	-5.132049	-2.699730	-1.010603
H	-6.503486	-1.177590	0.406948

Structure **TS3_d**

C	-0.828188	-0.959842	1.215131
C	-1.482443	-0.803469	-0.051785
C	1.229224	-0.956886	0.005734
C	0.552941	-1.168393	1.191590
H	1.118566	-1.396552	2.085261
N	0.506929	-0.722065	-1.109166
N	-0.816487	-0.662912	-1.171889
O	-0.985049	1.050400	1.902710
C	-0.268777	1.744702	1.059334
C	1.134818	1.898784	1.227006
C	-0.843650	2.297983	-0.118488
C	1.911401	2.531357	0.259995
H	1.589297	1.498297	2.129883
C	-0.056910	2.921350	-1.078932
H	-1.918376	2.200297	-0.255088
C	1.329257	3.029292	-0.908843
H	2.984540	2.621395	0.410434
H	-0.522607	3.321017	-1.976243
H	1.940286	3.510811	-1.665990
H	-1.412326	-1.227431	2.084976
H	0.961228	-0.626266	-2.010743
C	-2.967523	-0.753060	-0.146928
C	-3.712834	-0.218044	0.907654
N	-3.523050	-1.236098	-1.266503
C	-5.099938	-0.189916	0.790447
H	-3.201620	0.198958	1.768551
C	-4.853064	-1.200302	-1.363410
C	-5.686599	-0.692496	-0.365967

H	-5.708851	0.224723	1.587152
H	-5.276600	-1.602201	-2.280180
H	-6.762206	-0.693480	-0.501740
C	2.706693	-0.999610	-0.110905
C	3.382548	-0.249560	-1.075279
N	3.327300	-1.775562	0.786856
C	4.771993	-0.335044	-1.121418
H	2.852385	0.418441	-1.746686
C	4.657024	-1.843692	0.733178
C	5.424900	-1.150250	-0.204794
H	5.330115	0.236492	-1.855126
H	5.134007	-2.481953	1.471605
H	6.504484	-1.246701	-0.202759

Structure **int5_a**

C	-0.924291	-1.460357	0.899762
C	-1.519790	-0.830416	-0.220629
C	1.185169	-1.020230	-0.109699
C	0.445521	-1.572483	0.940945
H	0.945463	-2.046611	1.775346
N	0.508129	-0.475364	-1.124053
N	-0.800774	-0.351990	-1.217555
O	-1.092191	0.930735	2.220494
C	-0.267162	1.530322	1.440136
C	1.155037	1.456066	1.622540
C	-0.704605	2.274428	0.293745
C	2.041669	2.019252	0.711225
H	1.525925	0.920980	2.494930
C	0.194398	2.819165	-0.611037
H	-1.776928	2.368860	0.134315
C	1.579129	2.680575	-0.431385
H	3.112918	1.915998	0.877261
H	-0.183120	3.350381	-1.482291
H	2.276550	3.101158	-1.148875
H	-1.558641	-1.854827	1.681465
H	1.054952	-0.076730	-1.889516
C	-2.992268	-0.655321	-0.316866
C	-3.552903	0.202871	-1.265928
N	-3.719989	-1.354843	0.561973
C	-4.937545	0.331107	-1.301206
H	-2.913715	0.753840	-1.945825
C	-5.046746	-1.223626	0.515503
C	-5.703893	-0.395888	-0.394899
H	-5.408583	0.991007	-2.022092
H	-5.611824	-1.805578	1.238355
H	-6.785630	-0.326520	-0.385487
C	2.660235	-0.985486	-0.228380
C	3.491640	-1.378702	0.818139
N	3.115182	-0.511247	-1.394878
C	4.867697	-1.265322	0.635761
H	3.089242	-1.745791	1.754745
C	4.432145	-0.406850	-1.558538
C	5.350119	-0.769336	-0.570398
H	5.548413	-1.555878	1.428368
H	4.770009	-0.018740	-2.514841
H	6.413318	-0.661934	-0.750958

Structure **int5_b**

C	-0.907796	-0.932964	1.412135
C	-1.515475	-0.859315	0.134070
C	1.191104	-0.974666	0.296435
C	0.463516	-1.021035	1.487548
H	0.970163	-1.093592	2.440901
N	0.502515	-0.928035	-0.848828
N	-0.807612	-0.866091	-0.978868
O	-1.039195	1.734148	1.742329
C	-0.250004	1.980360	0.757547
C	1.176903	1.998818	0.904500
C	-0.737645	2.190892	-0.574626
C	2.024852	2.159935	-0.186258
H	1.583607	1.859988	1.904635
C	0.122806	2.338792	-1.652588
H	-1.816103	2.197007	-0.721008
C	1.514862	2.304794	-1.480455
H	3.102328	2.143067	-0.030055
H	-0.291193	2.468953	-2.650196
H	2.181412	2.412334	-2.330282
H	-1.522222	-0.966495	2.302548
H	1.042843	-0.898688	-1.715592
C	-2.990807	-0.744665	-0.015550
C	-3.703968	0.061430	0.874249
N	-3.556515	-1.424120	-1.020552
C	-5.083777	0.155809	0.711479
H	-3.166563	0.628019	1.629072
C	-4.879978	-1.324330	-1.159931
C	-5.687154	-0.553662	-0.321631
H	-5.673819	0.778683	1.375670
H	-5.318913	-1.888805	-1.978229
H	-6.758112	-0.513277	-0.485285
C	2.665485	-0.986241	0.161673
C	3.505452	-0.900998	1.269972
N	3.110288	-1.045019	-1.099612
C	4.879609	-0.872353	1.046122
H	3.110516	-0.842143	2.277194
C	4.425869	-1.016105	-1.302421
C	5.351697	-0.928448	-0.260713
H	5.566896	-0.801930	1.882003
H	4.755585	-1.064695	-2.335891
H	6.413105	-0.905037	-0.478440

Structure **int5_c**

C	-0.892048	-1.491584	0.809855
C	-1.528851	-0.807496	-0.255194
C	1.184743	-0.958675	-0.231697
C	0.478266	-1.583826	0.803723
H	1.038228	-2.087753	1.580275
N	0.472695	-0.348470	-1.183299
N	-0.845789	-0.245891	-1.231398
O	-1.097448	0.921589	2.172170
C	-0.266480	1.533612	1.409632
C	1.155229	1.436967	1.588797
C	-0.695103	2.318689	0.286374
C	2.047961	2.008199	0.689538
H	1.520334	0.870921	2.443221
C	0.209807	2.869609	-0.608440
H	-1.766135	2.435468	0.133478
C	1.593491	2.700513	-0.439074
H	3.117800	1.886091	0.851987
H	-0.161153	3.430121	-1.463991
H	2.295714	3.131537	-1.145746
H	-1.498468	-1.937396	1.586586
H	0.938841	0.184625	-1.912772
C	-3.006811	-0.658876	-0.307514
C	-3.608189	0.233908	-1.197722
N	-3.697580	-1.417799	0.551461
C	-4.995593	0.335074	-1.191809

H	-2.997748	0.830985	-1.864889
C	-5.027369	-1.312245	0.544629
C	-5.723802	-0.452732	-0.305180
H	-5.497813	1.020458	-1.866385
H	-5.562196	-1.942182	1.250034
H	-6.806089	-0.406618	-0.265432
C	2.663331	-0.951958	-0.314353
C	3.345967	-0.606415	-1.481157
N	3.280057	-1.284623	0.826777
C	4.738245	-0.571278	-1.443237
H	2.831966	-0.387822	-2.410938
C	4.609939	-1.253373	0.847745
C	5.385816	-0.891379	-0.257045
H	5.300599	-0.303059	-2.330787
H	5.081890	-1.526709	1.787135
H	6.466825	-0.874313	-0.181153

Structure **int5_d**

C	-0.880356	-1.021798	1.316494
C	-1.528855	-0.840160	0.069121
C	1.185560	-0.966065	0.133195
C	0.490994	-1.117584	1.337486
H	1.056477	-1.272463	2.246676
N	0.462823	-0.795458	-0.979370
N	-0.856873	-0.727064	-1.057847
O	-1.046530	1.646318	1.743876
C	-0.243955	1.945290	0.785909
C	1.181532	1.937496	0.949541
C	-0.713790	2.246157	-0.536015
C	2.043836	2.144864	-0.121312
H	1.575089	1.730292	1.942453
C	0.160503	2.438048	-1.595373
H	-1.790295	2.278075	-0.692597
C	1.550654	2.365673	-1.412168
H	3.119040	2.102454	0.045432
H	-0.239954	2.633130	-2.587918
H	2.228187	2.512635	-2.247400
H	-1.466219	-1.132008	2.220294
H	0.922021	-0.606722	-1.866296
C	-3.009567	-0.733687	-0.029064
C	-3.710933	-0.031245	0.953036
N	-3.592272	-1.322157	-1.080661
C	-5.096011	0.052922	0.836100
H	-3.163697	0.470488	1.744968
C	-4.920348	-1.233242	-1.176027
C	-5.716315	-0.563196	-0.245745
H	-5.677056	0.597100	1.573321
H	-5.372456	-1.722967	-2.034328
H	-6.791847	-0.526602	-0.377142
C	2.663721	-0.996164	0.041212
C	3.334507	-1.149277	-1.172452
N	3.292322	-0.848057	1.214373
C	4.727210	-1.107123	-1.164828
H	2.812199	-1.320535	-2.107404
C	4.622308	-0.817725	1.208838
C	5.386967	-0.930063	0.044192
H	5.280310	-1.218245	-2.090936
H	5.103753	-0.695884	2.174907
H	6.468709	-0.888864	0.096665

Structure **3a**

C	1.854801	0.030136	0.000032
C	1.172576	-1.188594	-0.000053
C	1.126949	1.219423	-0.000029
C	-0.219351	-1.222870	0.000055
H	1.727030	-2.121954	-0.000074
C	-0.267778	1.199460	-0.000011
H	1.642655	2.174692	-0.000062
C	-0.937708	-0.025256	0.000140
H	-0.760802	-2.163385	0.000067
H	-0.832703	2.127715	-0.000138
H	2.939307	0.051390	0.000032
O	-2.299808	-0.113800	-0.000174
H	-2.693959	0.768150	0.00070

Structure **4a_a**

C	0.687375	-1.333290	0.044917
C	1.337842	-0.089470	0.003360
C	-1.337839	-0.089469	0.003339
C	-0.687372	-1.333290	0.044908
H	-1.269372	-2.246092	0.077008
N	-0.660074	1.059488	-0.034766
N	0.660080	1.059488	-0.034769
H	1.269376	-2.246092	0.077020
C	2.824964	0.009900	-0.000049
C	3.467535	1.251780	0.018822
N	3.492726	-1.151668	-0.020626
C	4.857882	1.280329	0.014795
H	2.880608	2.161526	0.037073
C	4.826444	-1.109721	-0.024519
C	5.557696	0.077124	-0.007635
H	5.385935	2.228207	0.029805
H	5.335901	-2.069588	-0.042194
H	6.641440	0.051277	-0.011622
C	-2.824964	0.009903	-0.000075
C	-3.467535	1.251799	0.017707
N	-3.492730	-1.151680	-0.019586
C	-4.857882	1.280344	0.013734
H	-2.880612	2.161564	0.035083
C	-4.826448	-1.109738	-0.023434
C	-5.557699	0.077121	-0.007566
H	-5.385934	2.228236	0.027898
H	-5.335906	-2.069621	-0.040206
H	-6.641444	0.051271	-0.011462

Structure **4a_b**

C	-0.692852	1.309741	-0.269731
C	-1.338027	0.089782	-0.017596
C	1.337972	0.092731	-0.025127
C	0.682816	1.309463	-0.273473
H	1.246985	2.212256	-0.481730
N	0.664300	-1.036078	0.208409
N	-0.656071	-1.034676	0.210948
H	-1.276000	2.202093	-0.460650
C	-2.825044	-0.012440	0.005115
C	-3.462330	-1.231873	0.253415
N	-3.496746	1.123977	-0.224145
C	-4.852645	-1.264646	0.261799
H	-2.871515	-2.121320	0.433235
C	-4.830299	1.078336	-0.213149
C	-5.556946	-0.087675	0.024076
H	-5.377175	-2.195511	0.451255
H	-5.343468	2.017402	-0.402799
H	-6.640784	-0.066124	0.020995
C	2.825636	0.001983	-0.006482
C	3.584126	1.052429	0.519152

N	3.376644	-1.112217	-0.506656
C	4.971255	0.940025	0.522385
H	3.098964	1.928402	0.936109
C	4.707970	-1.205265	-0.499365
C	5.550080	-0.211958	0.000775
H	5.585172	1.735958	0.930801
H	5.125087	-2.117814	-0.917336
H	6.625584	-0.346179	-0.020544

Structure **4a_c**

C	-0.688290	1.337976	0.003817
C	-1.337971	0.095107	0.007040
C	1.337953	0.095148	-0.006840
C	0.688226	1.337995	-0.003599
H	1.253433	2.263527	-0.018990
N	0.660387	-1.056038	-0.002983
N	-0.660377	-1.056054	0.003458
H	-1.253560	2.263468	0.019267
C	-2.825544	-0.000844	0.008120
C	-3.586179	0.912098	-0.728505
N	-3.373380	-0.984009	0.734512
C	-4.973093	0.798549	-0.706180
H	-3.103135	1.680184	-1.322970
C	-4.704509	-1.079546	0.748633
C	-5.549029	-0.217055	0.049571
H	-5.589061	1.488034	-1.273926
H	-5.119098	-1.883203	1.351704
H	-6.624203	-0.345784	0.100297
C	2.825557	-0.000834	-0.008105
C	3.586340	0.912106	0.728467
N	3.373247	-0.983959	-0.734568
C	4.973200	0.798502	0.705980
H	3.103357	1.680192	1.322976
C	4.704436	-1.079524	-0.748889
C	5.549024	-0.217143	-0.049886
H	5.589335	1.487880	1.273675
H	5.118859	-1.883202	-1.352038
H	6.624194	-0.345882	-0.100671

9. NMR SPECTRA

Figure S6: ^{1}H NMR ($CDCl_3$, 400 MHz) of **1b**

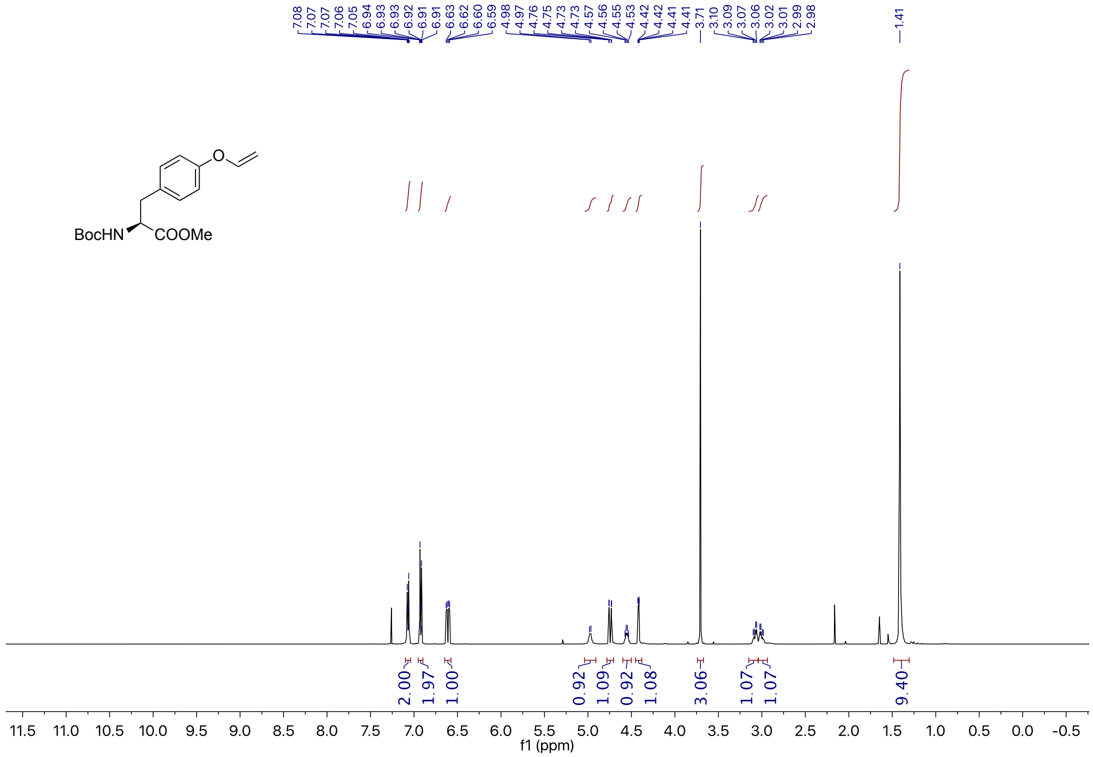

Figure S7: ^{13}C NMR ($CDCl_3$, 126 MHz) of **1b**

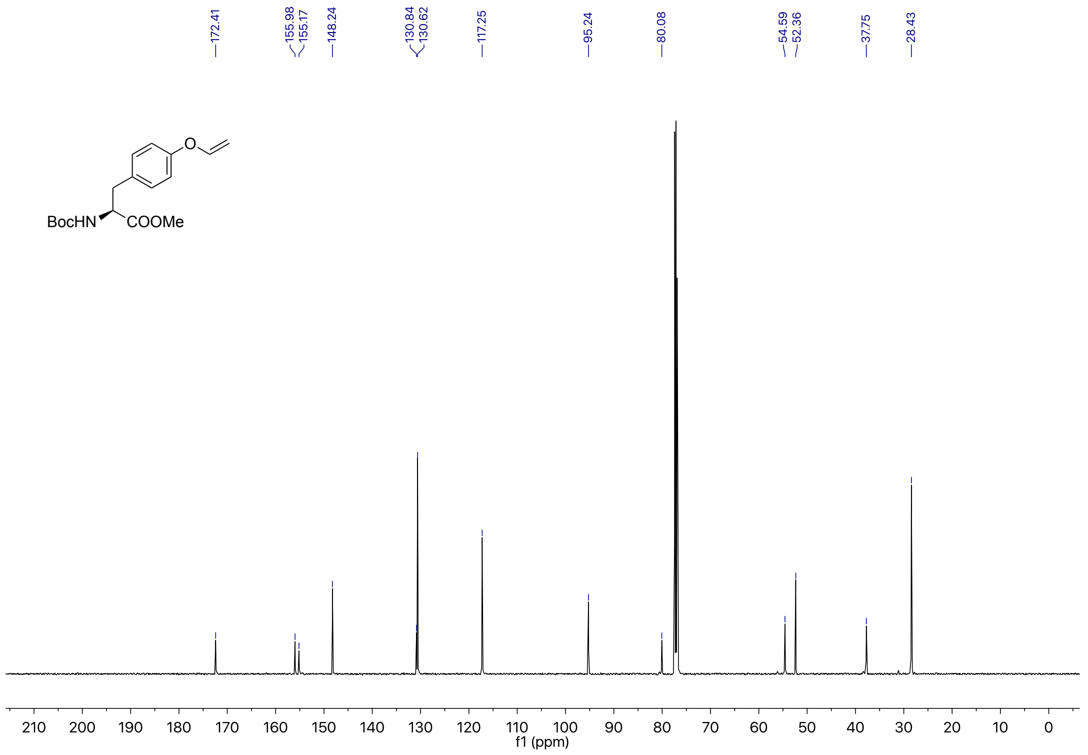

Figure S8: ^{1}H NMR ($CDCl_3$, 500 MHz) of **1c**

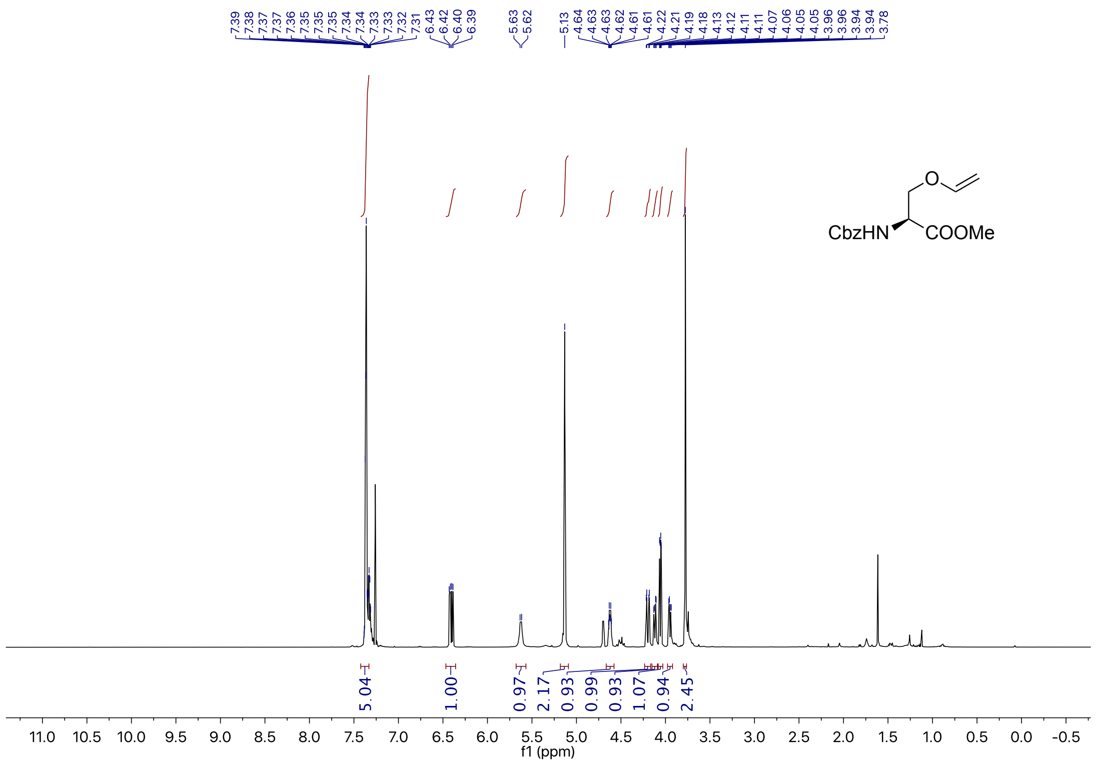

Figure S9: ^{13}C NMR ($CDCl_3$, 126 MHz) of **1c**

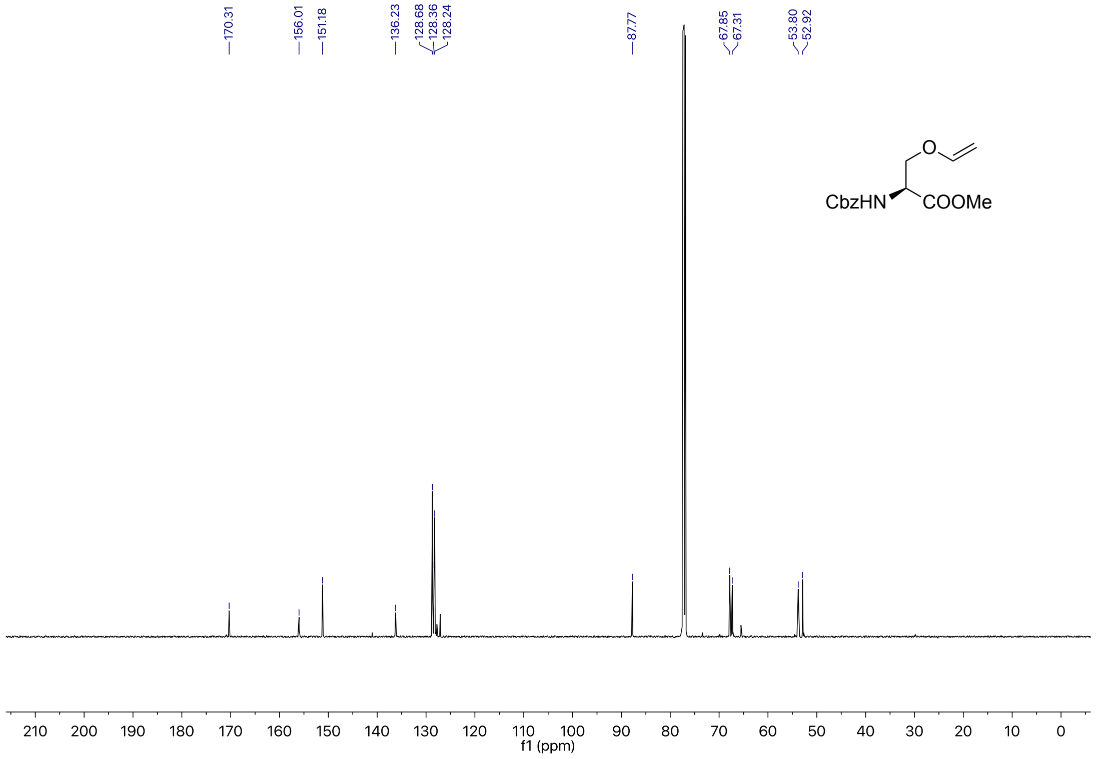

Figure S10: ^{1}H NMR ($CDCl_3$, 400 MHz) of **1d**

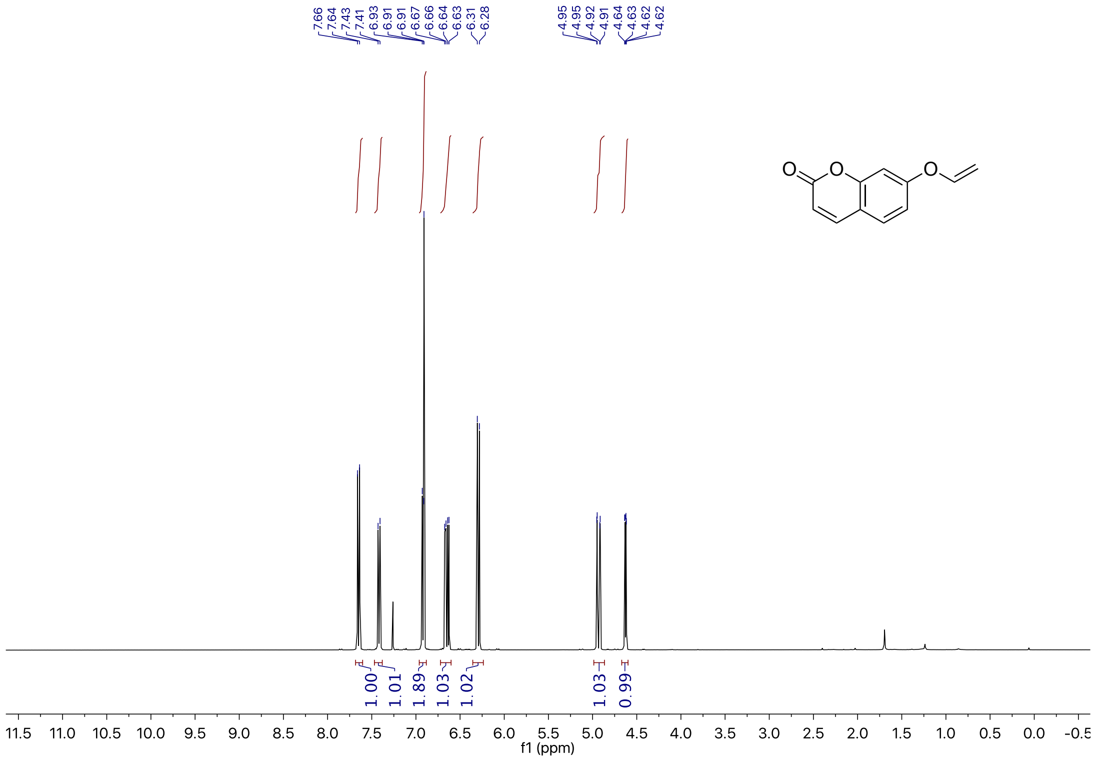

Figure S11: ^{13}C NMR ($CDCl_3$, 101 MHz) of **1d**

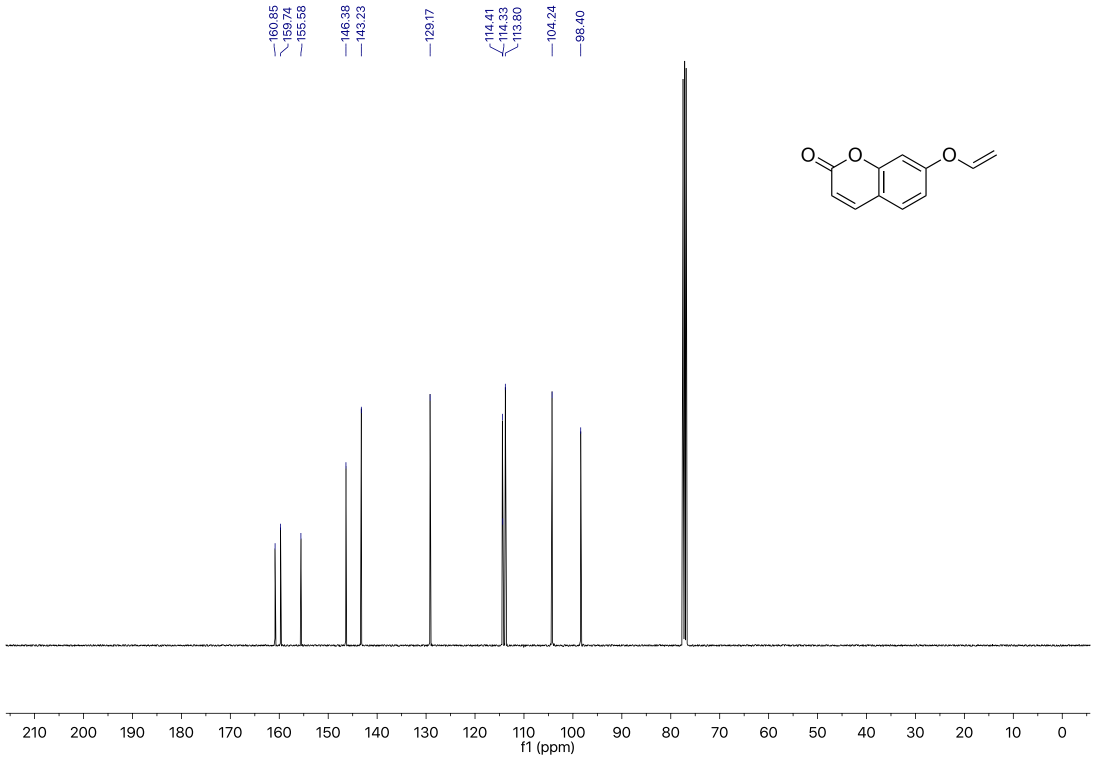

Figure S12: ^{1}H NMR ($CDCl_3$, 500 MHz) of **1e**

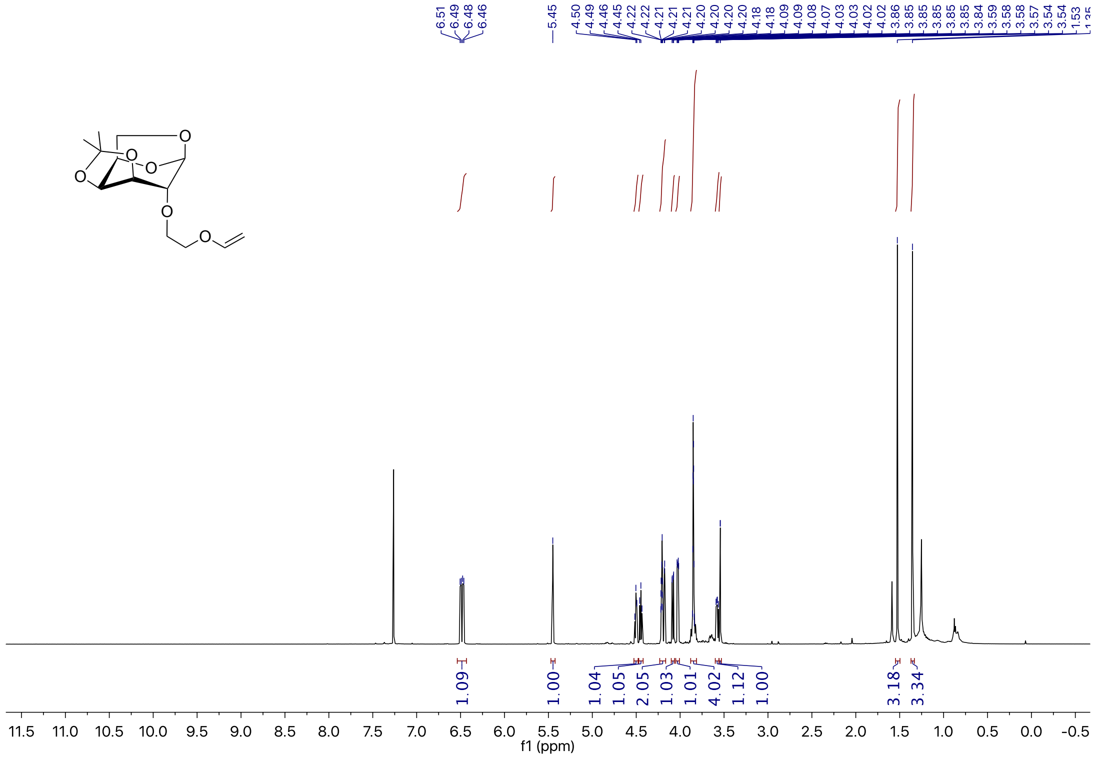

Figure S13: ^{13}C NMR ($CDCl_3$, 126 MHz) of **13**

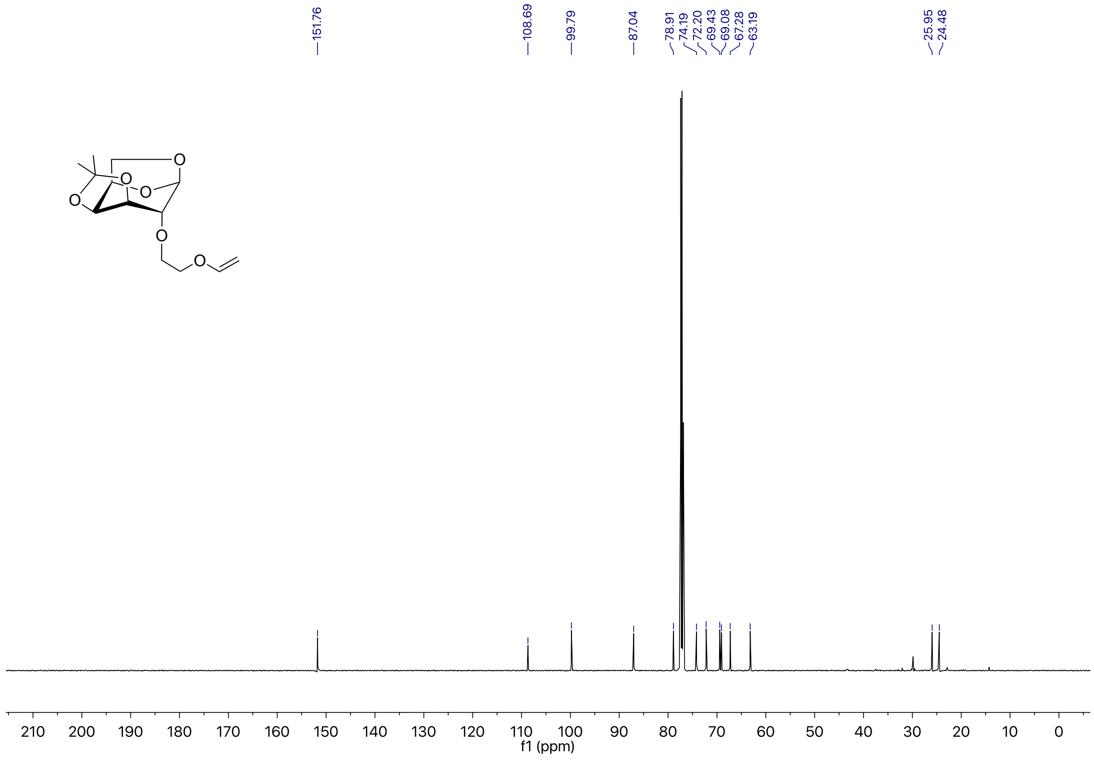

Figure S14: ^{1}H NMR ($CDCl_3$, 400 MHz) of **5**

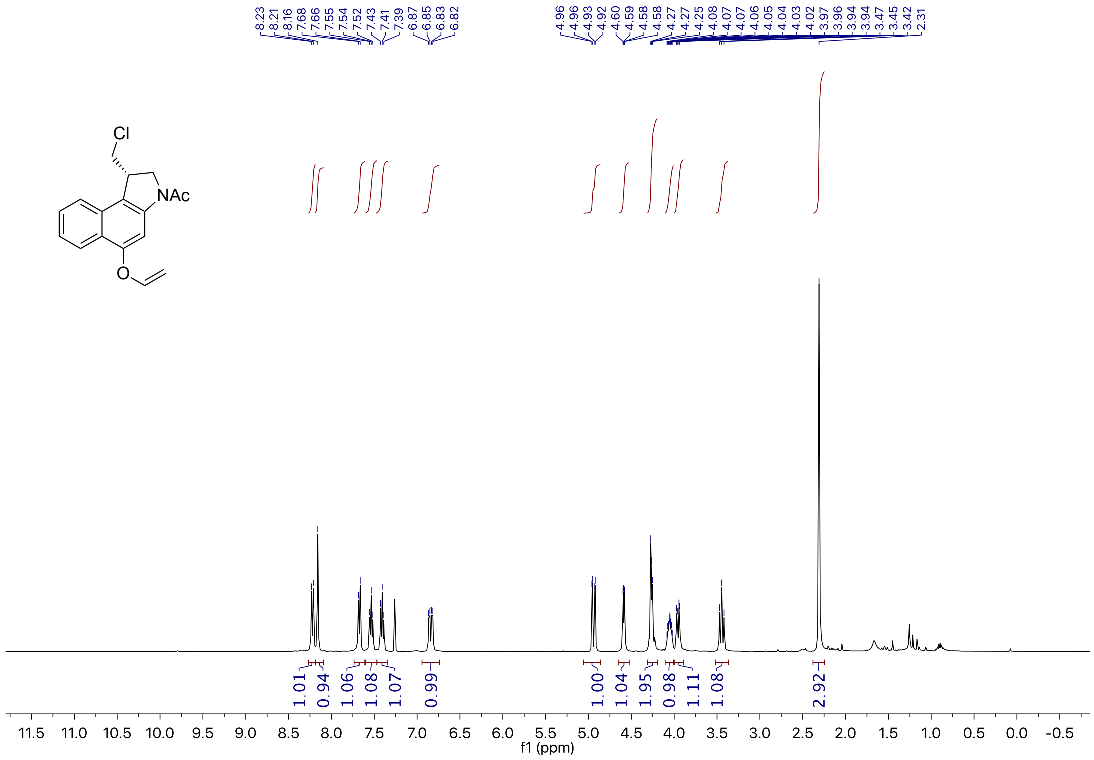

Figure S15: ^{13}C NMR ($CDCl_3$, 101 MHz) of **5**

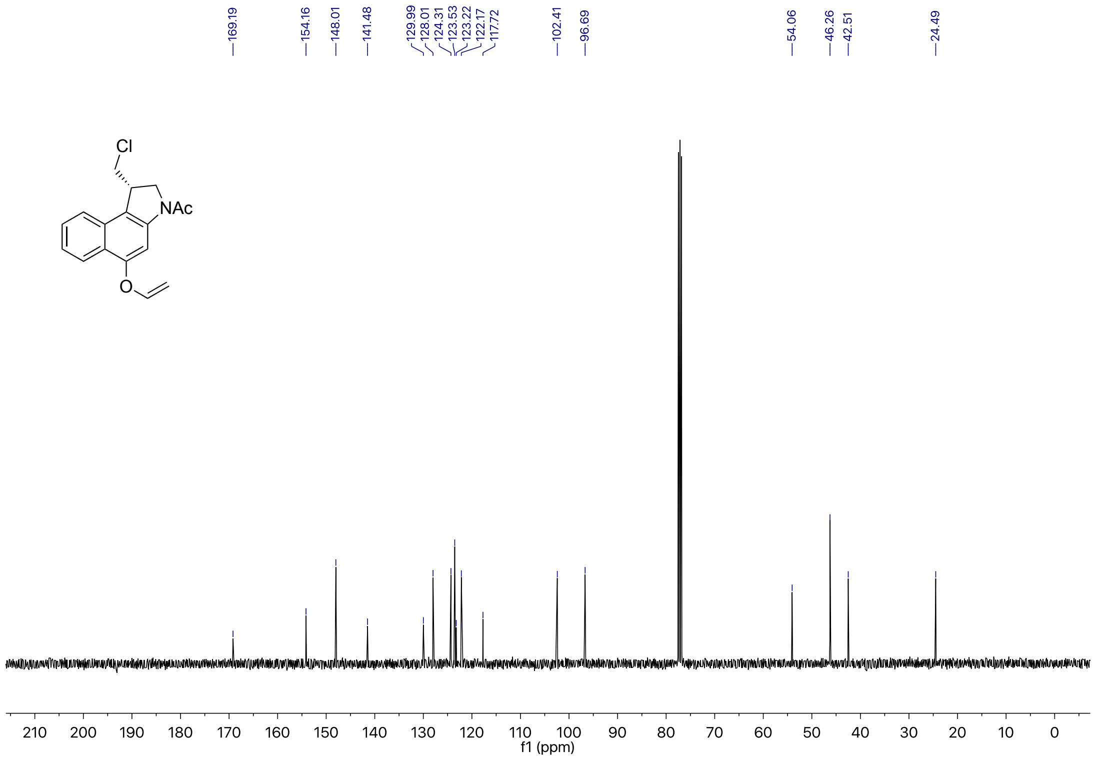

Figure S16: ^{1}H NMR ($CDCl_3$, 500 MHz) of **4a**

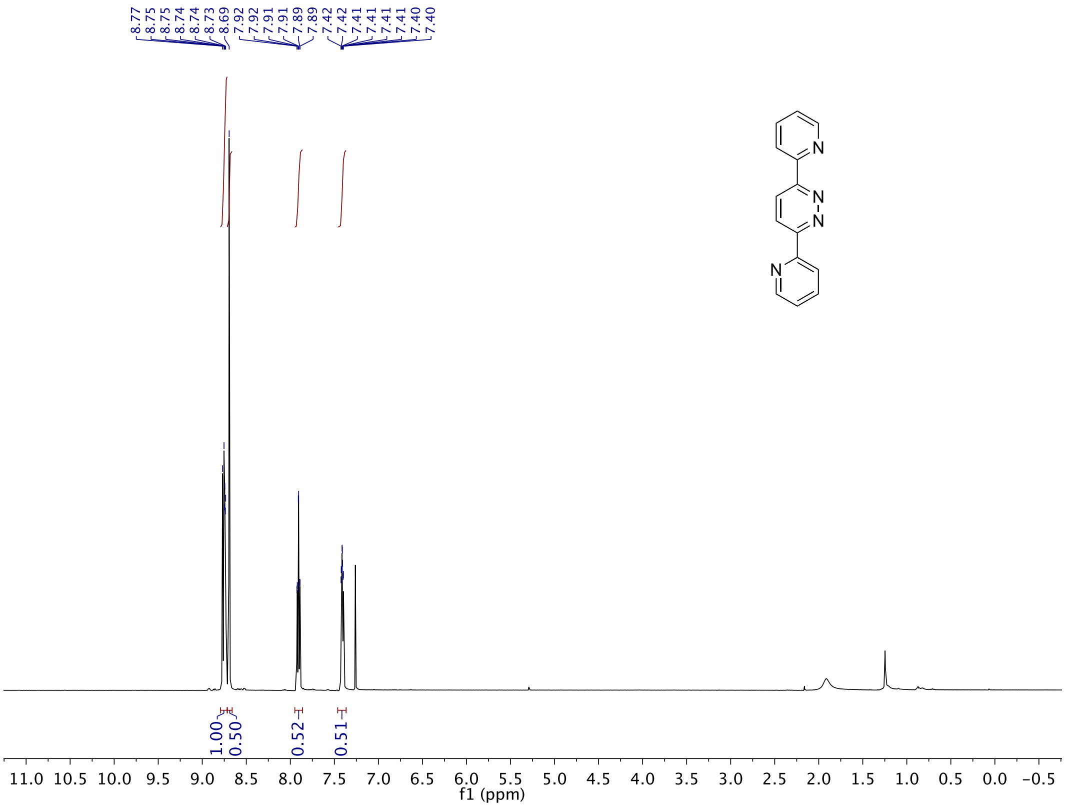

Figure S17: ^{13}C NMR ($CDCl_3$, 126 MHz) of **4a**

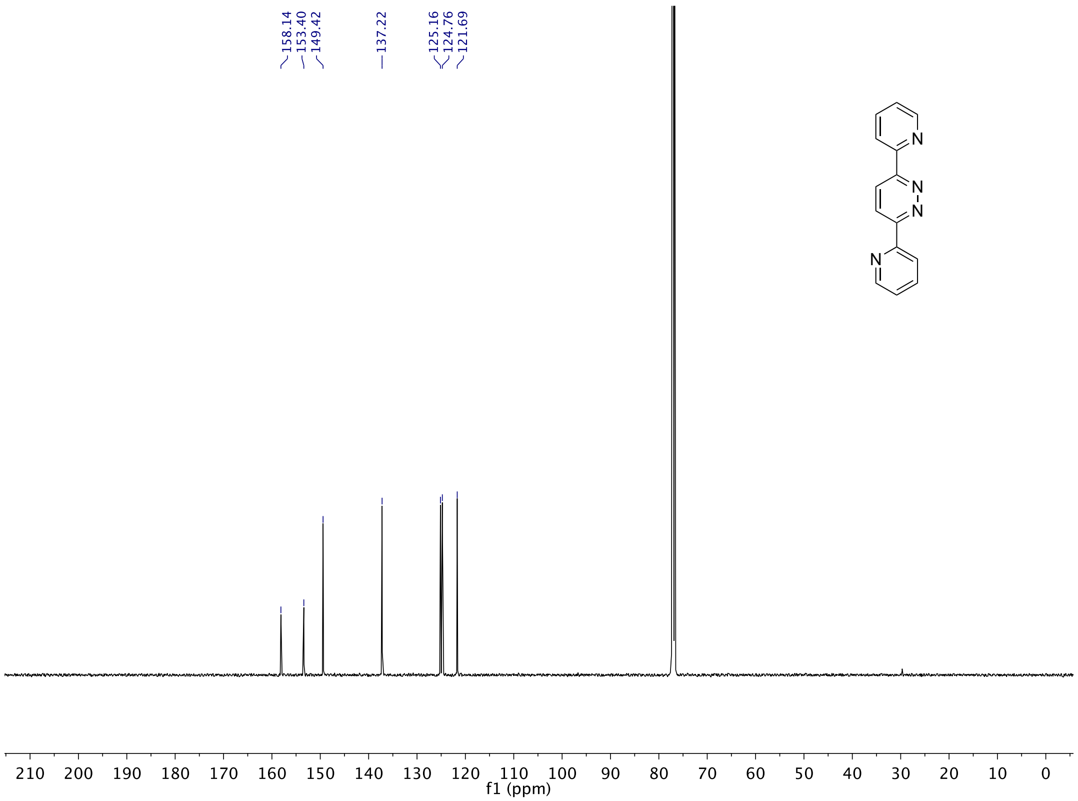

Figure S18: ^{1}H NMR ($CDCl_3$, 400 MHz) of **3b**

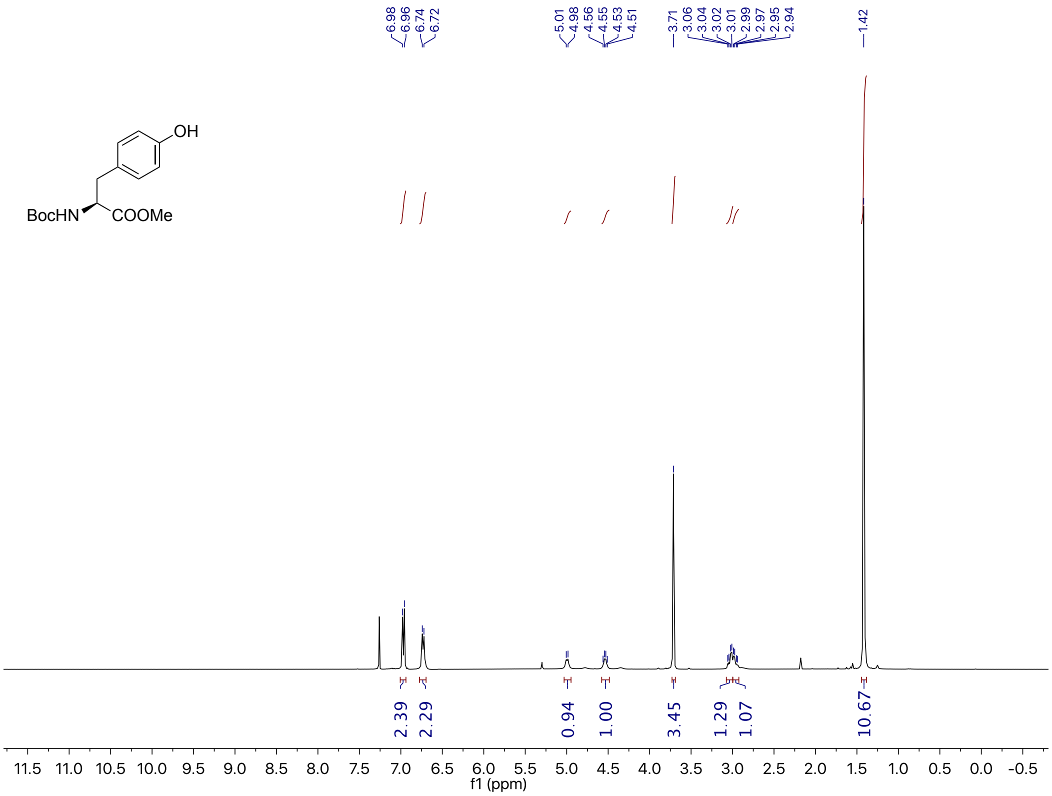

Figure S19: ^{13}C NMR ($CDCl_3$, 101 MHz) of **3b**

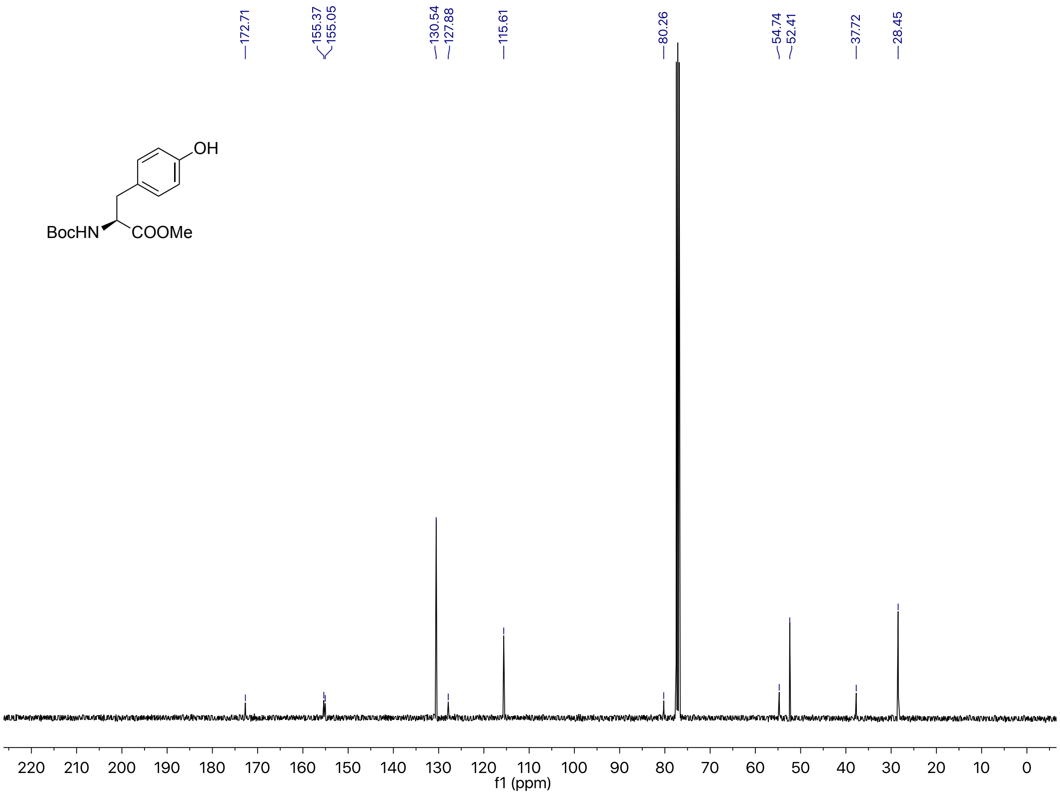

Figure S20: ^{1}H NMR ($CDCl_3$, 400 MHz) of **3c**

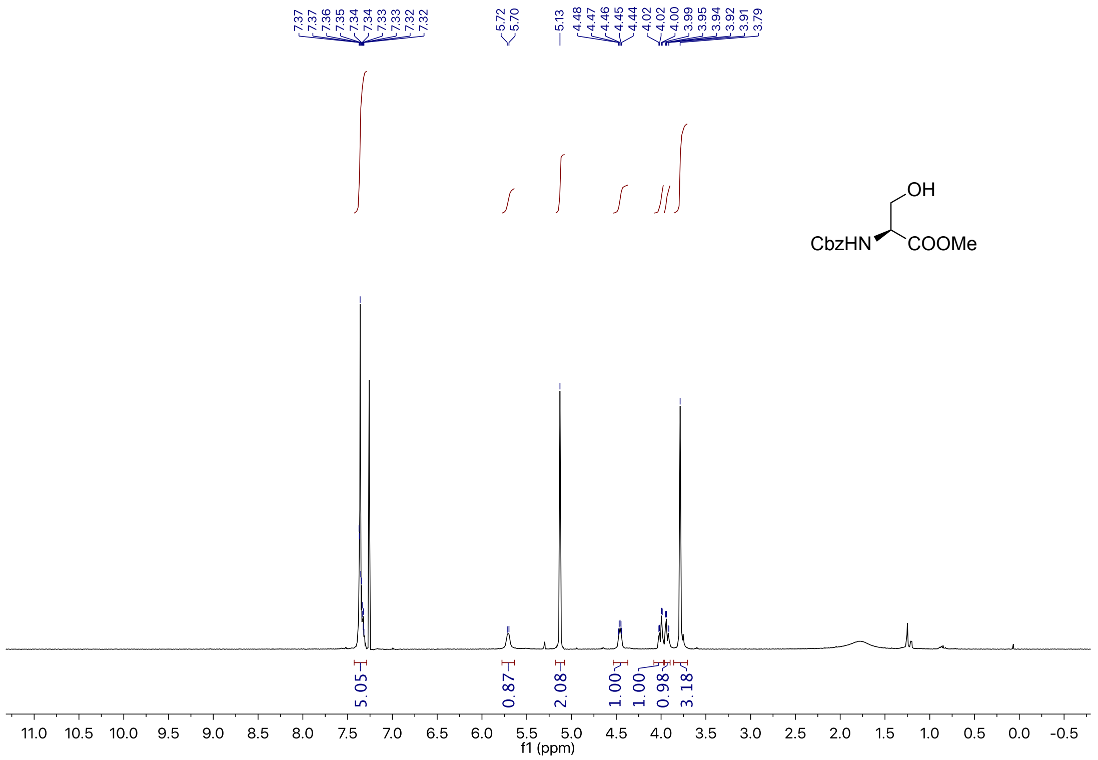

Figure S21: ^{13}C NMR ($CDCl_3$, 101 MHz) of **3c**

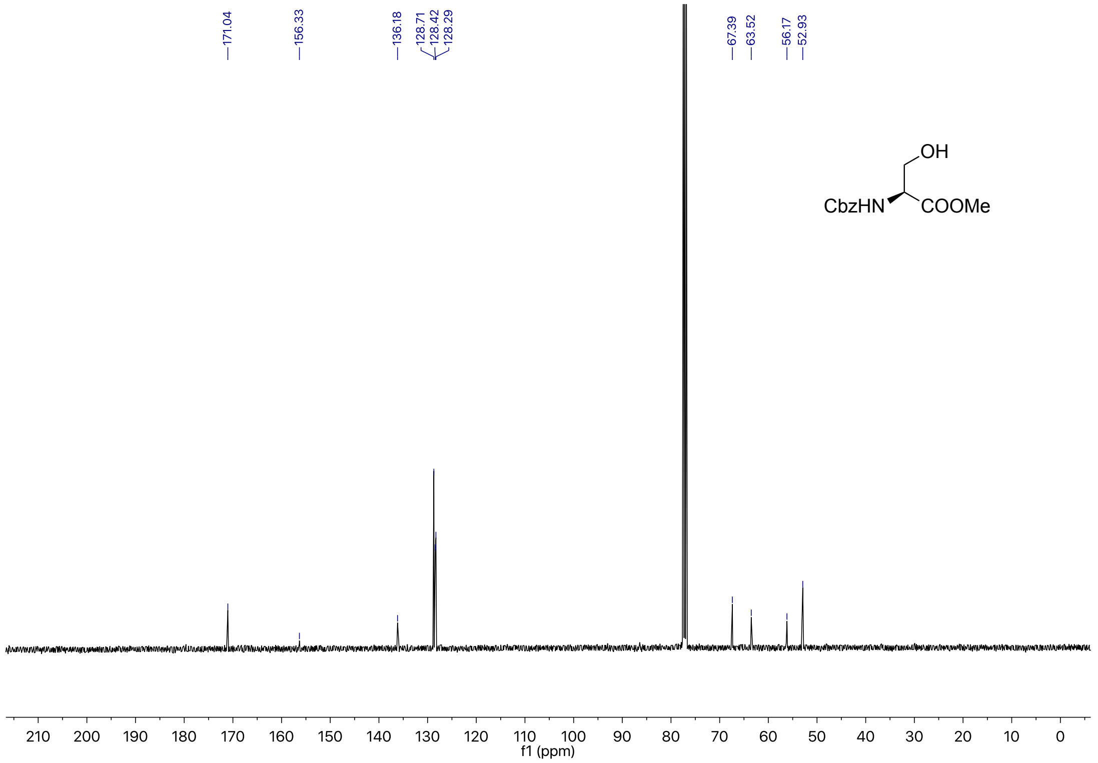

Figure S22: ^{1}H NMR (MeOD, 500 MHz) of **3d**

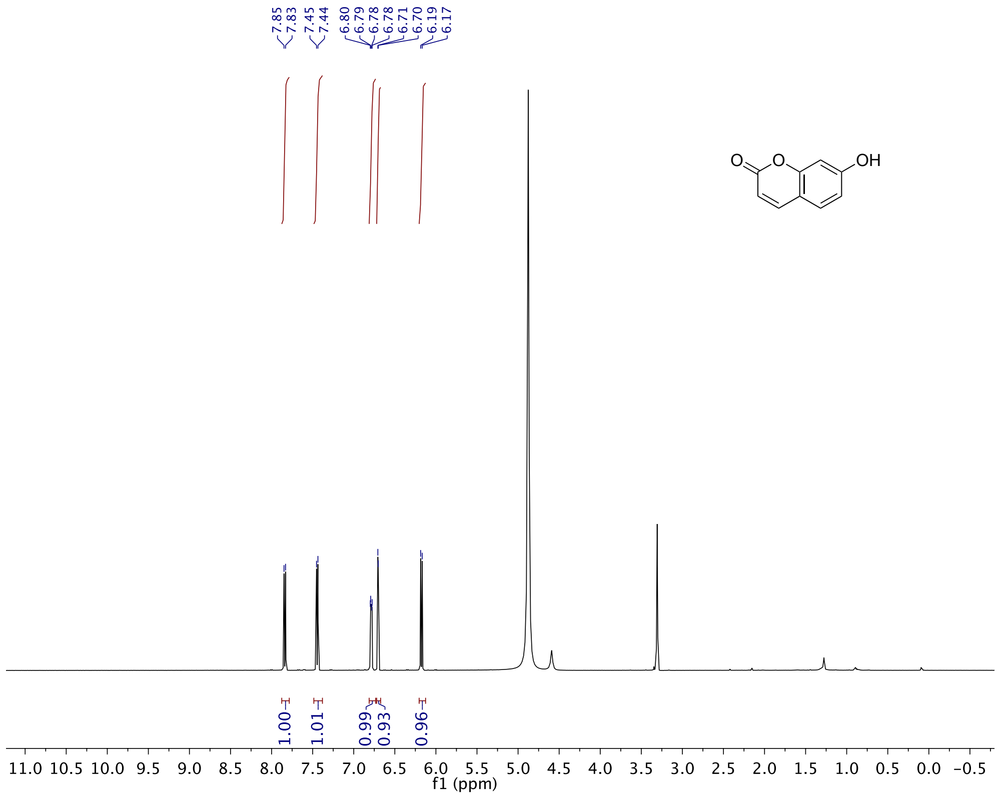

Figure S23: ^{13}C NMR (MeOD, 126 MHz) of **3d**

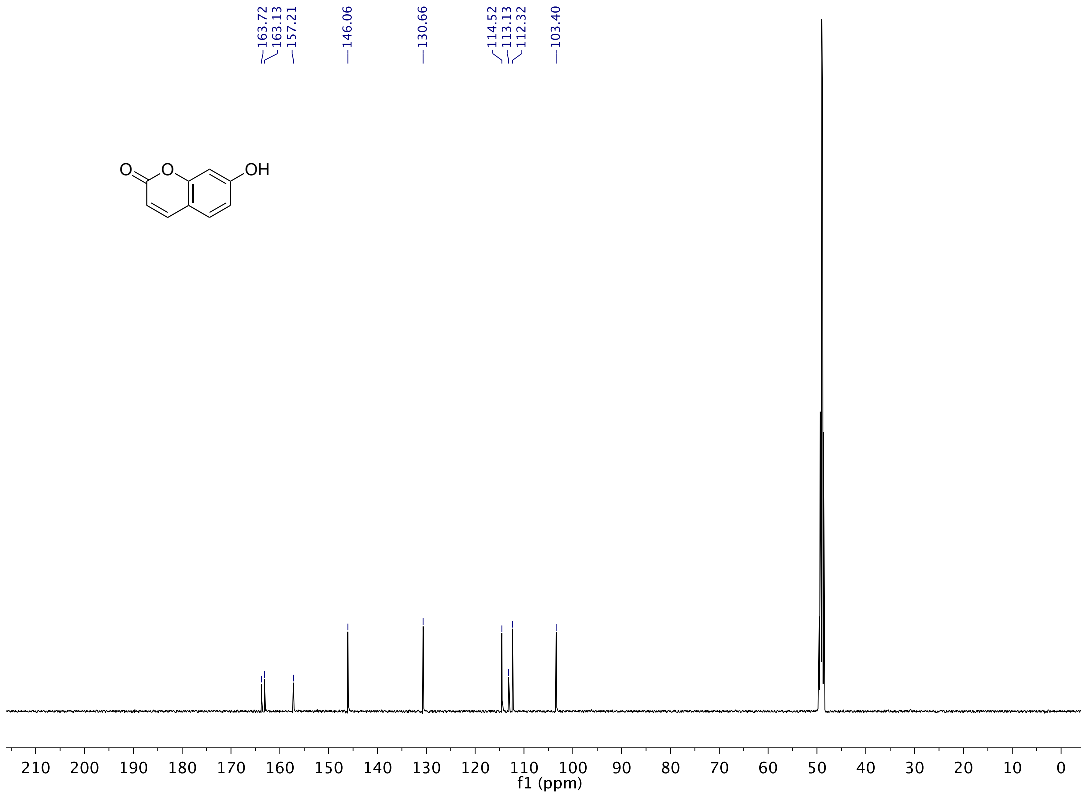

Figure S24: ^{1}H NMR ($CDCl_3$, 400 MHz) of **3e**

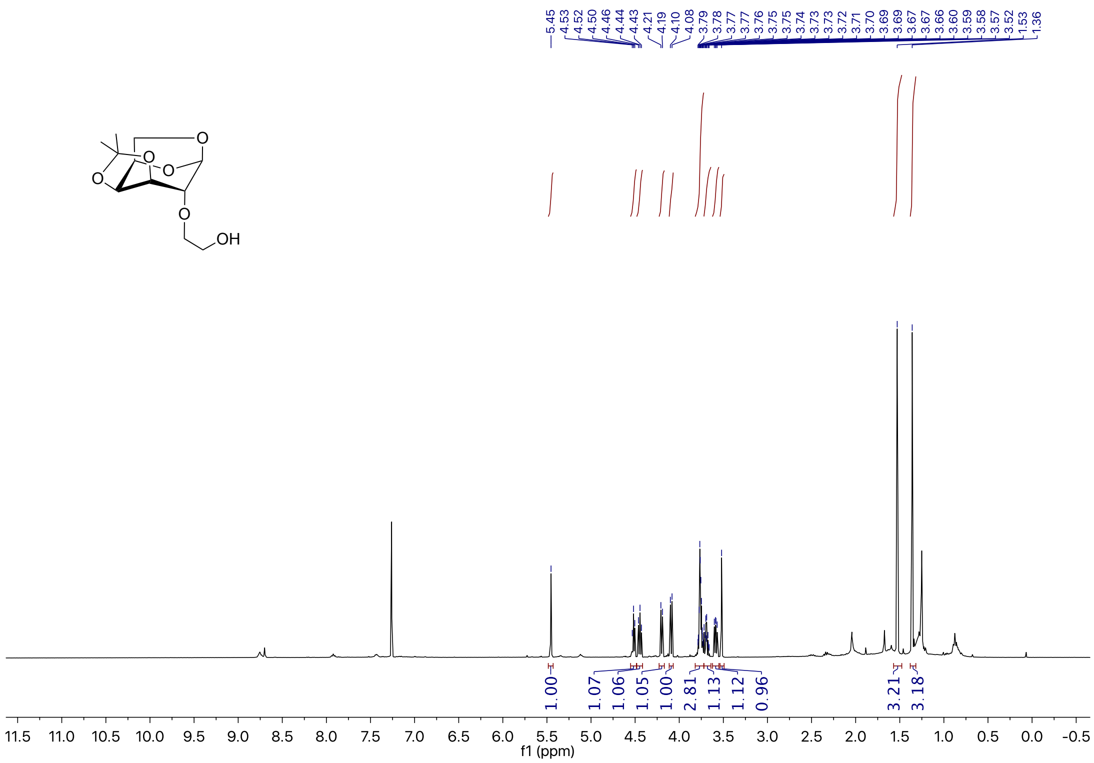

Figure S25: ^{13}C NMR ($CDCl_3$, 101 MHz) of **3e**

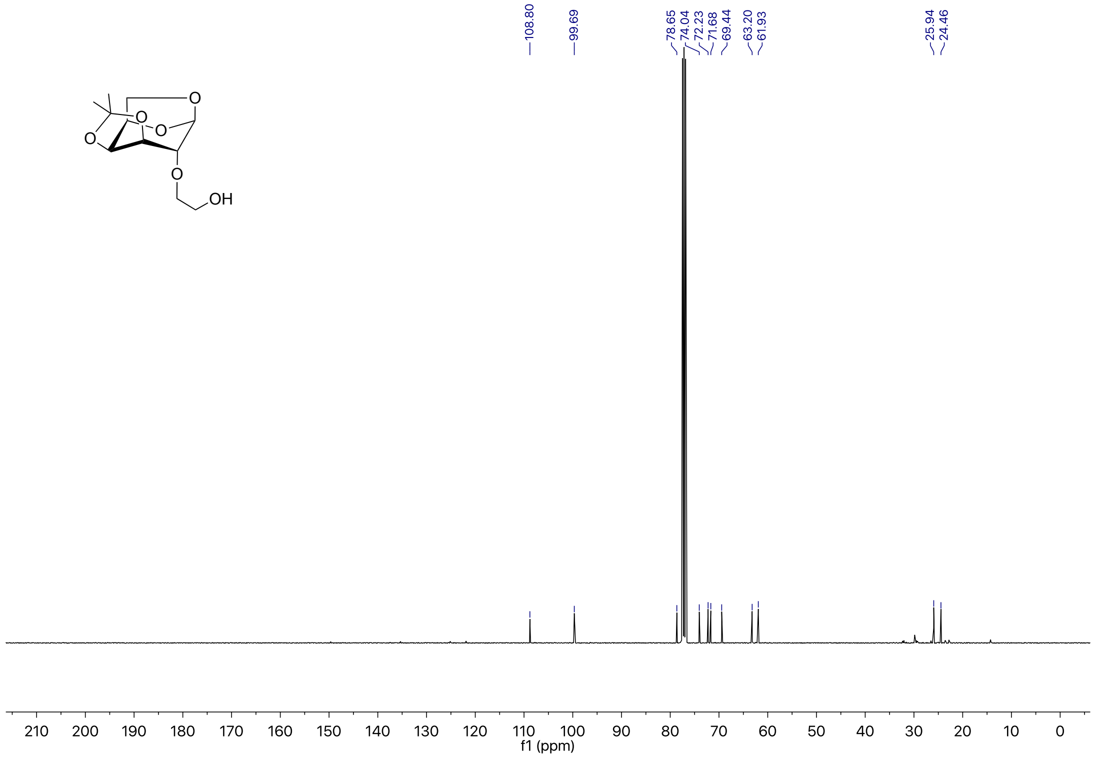

Figure S26: ^{1}H NMR (DMSO-d_6, 400 MHz) of **2c**

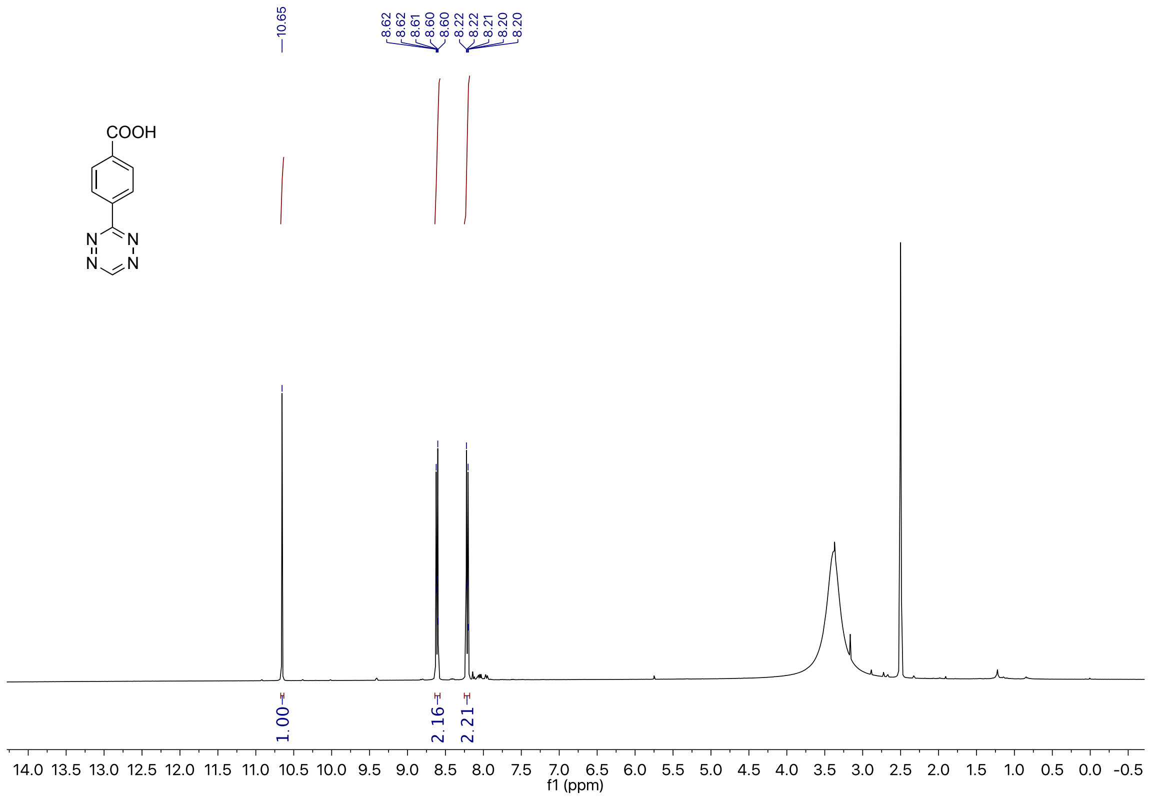

Figure S27: ^{13}C NMR (DMSO-d_6, 101 MHz) of **2c**

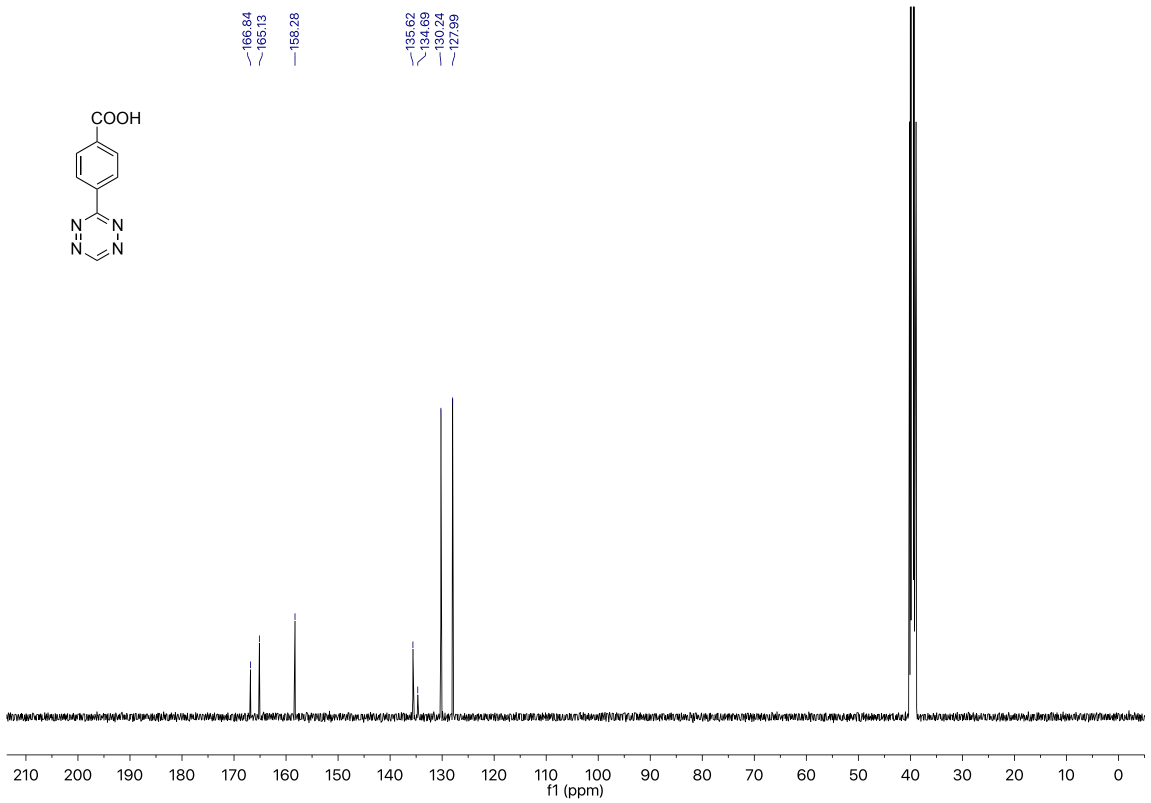

10. REFERENCES

[1] M. Blouin, R. Frenette, *J. Org. Chem.* **2001**, *66*, 9043–9045.

[2] M. Bosch, M. Schlaf, *J. Org. Chem.* **2003**, *68*, 5225–5227.

[3] M. Tercel, S. M. Stribbling, H. Sheppard, B. G. Siim, K. Wu, S. M. Pullen, K. J. Botting, W. R. Wilson, W. A. Denny, *J. Med. Chem.* **2003**, *46*, 2132–2151.

[4] a) R. Hoogenboom, B. C. Moore, U. S. Schubert, *J. Org. Chem.* **2006**, *71*, 4903–4909; b) Y. Xiao, Y. Xu, H.-S. Cheon, J. Chae, *J. Org. Chem.* **2013**, *78*, 5804–5809.

[5] M. J. Frisch, G. W. Trucks, H. B. Schlegel, G. E. Scuseria, M. A. Robb, J. R. Cheeseman, G. Scalmani, V. Barone, B. Mennucci, G. A. Petersson, H. Nakatsuji, M. Caricato, X. Li, H. P. Hratchian, A. F. Izmaylov, J. Bloino, G. Zheng, J. L. Sonnenberg, M. Hada, M. Ehara, K. Toyota, R. Fukuda, J. Hasegawa, M. Ishida, T. Nakajima, Y. Honda, O. Kitao, H. Nakai, T. Vreven, J. A. Montgomery Jr., J. E. Peralta, F. Ogliaro, M. J. Bearpark, J. Heyd, E. N. Brothers, K. N. Kudin, V. N. Staroverov, R. Kobayashi, J. Normand, K. Raghavachari, A. P. Rendell, J. C. Burant, S. S. Iyengar, J. Tomasi, M. Cossi, N. Rega, N. J. Millam, M. Klene, J. E. Knox, J. B. Cross, V. Bakken, C. Adamo, J. Jaramillo, R. Gomperts, R. E. Stratmann, O. Yazyev, A. J. Austin, R. Cammi, C. Pomelli, J. W. Ochterski, R. L. Martin, K. Morokuma, V. G. Zakrzewski, G. A. Voth, P. Salvador, J. J. Dannenberg, S. Dapprich, A. D. Daniels, Ö. Farkas, J. B. Foresman, J. V. Ortiz, J. Cioslowski, D. J. Fox, Gaussian, Inc., Wallingford, CT, USA, **2009**.

[6] Y. Zhao, D. G. Truhlar, *Theor. Chem. Acc.* **2008**, *120*, 215–241.

[7] G. Scalmani, M. J. Frisch, *J. Chem. Phys.* **2010**, *132*, 114110.

[8] R. F. Ribeiro, A. V. Marenich, C. J. Cramer, D. G. Truhlar, *J. Phys. Chem. B* **2011**, *115*, 14556–14562.

[9] a) C. Gonzalez, H. B. Schlegel, *J. Chem. Phys.* **1989**, *90*, 2154–2161; b) C. Gonzalez, H. B. Schlegel, *J. Phys. Chem.* **1990**, *94*, 5523–5527.
